# Fragment-Based Development of Small Molecule Inhibitors Targeting *Mycobacterium tuberculosis* Cholesterol Metabolism

**DOI:** 10.1021/acs.jmedchem.5c00478

**Published:** 2025-07-14

**Authors:** Madeline E. Kavanagh, Kirsty J. McLean, Sophie H. Gilbert, Cecilia N. Amadi, Matthew Snee, Richard B. Tunnicliffe, Kriti Arora, Helena I. M. Boshoff, Alexander Fanourakis, Maria Jose Rebollo-Lopez, Fatima Ortega, Colin W. Levy, Andrew W. Munro, David Leys, Chris Abell, Anthony G. Coyne

**Affiliations:** † Yusuf Hamied Department of Chemistry, 2152 University of Cambridge, Lensfield Road, Cambridge CB2 1EW, U.K.; ‡ Centre for Synthetic Biology of Fine and Specialty Chemicals (SYNBIOCHEM), Manchester Institute of Biotechnology, 5292 University of Manchester, 131 Princess Street, Manchester M1 7DN, U.K.; § Tuberculosis Research Section, Laboratory of Clinical Immunology and Microbiology, National Institutes of Health, Bethesda, Maryland 20892, United States; ∥ Global Health R&D, GSK, Severo Ochoa 2, Tres Cantos 28760, Spain; ⊥ Manchester Protein Structure Facility (MPSF), Manchester Institute of Biotechnology, 5292 University of Manchester, Manchester M1 7DN, U.K.; # Department of Chemistry, Manchester Institute of Biotechnology, 5292 University of Manchester, 131 Princess Street, Manchester M1 7DN, U.K.

## Abstract

Tuberculosis is the deadliest infectious disease in history and new drugs are urgently required to combat multidrug-resistant (MDR) strains of (*Mtb*). Here, we exploit the relience of *Mtb* on host-derived cholesterol to develop a novel class of antitubercular compounds that target *Mtb* CYP125 and CYP142; the enzymes that catalyze the first step of cholesterol metabolism. A combination of fragment screening and structure-based drug design was used to identify a hit compound and guide synthetic optimization of a dual CYP125/142 ligand **5m** (*K*_D_ 40–160 nM), which potently inhibits enzyme activity in vitro (*K*_I_ < 100 nM), and the growth of *Mtb* in extracellular (MIC_99_ 0.4–1.5 μM) and intracellular assays (IC_50_ 1.7 μM). The structural data and lead compounds reported here will help study *Mtb* cholesterol metabolism and guide the development of novel antibiotics to combat MDR *Mtb.*

## Graphical abstract

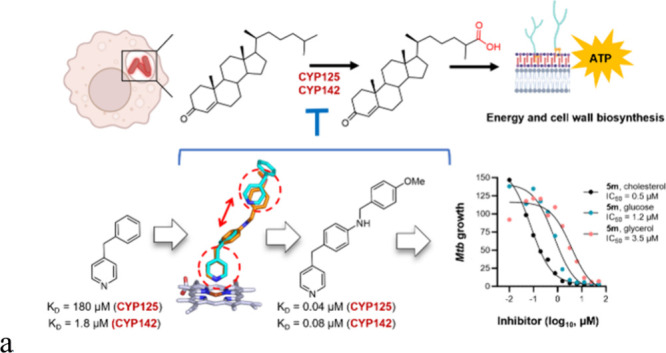

## Introduction

Tuberculosis (TB) is the world’s most deadly infectious disease, killing more than 1.3 million people every year.^1^ Although global TB deaths are declining, there has been an alarming increase in the number and distribution of cases caused by multi- (MDR) or extensively- (XDR) drug resistant strains of the causal bacterium (*Mtb*). Despite this impending threat, only two drugs (bedaquiline and pretomanid) with new mechanisms of action (MoA) have been approved for the treatment of TB in more than 50 years. Consequently, there is now an urgent need to develop new antitubercular drugs, in particular, compounds with activity against recalcitrant *Mtb* populations, such as nonreplicating bacteria and MDR-TB.^2^

*Mtb* is a facultative intracellular pathogen with unique metabolic adaptations that enable the bacteria to survive long-term in the harsh, nutrient-poor environment of the host macrophage.^3–6^ The development of drugs that specifically target bioenergetic pathways required for intracellular growth has recently emerged as a promising approach that could help address limitations of first and second line drugs.^2,7^ For example, bedaquiline, a diarylquinoline that targets the *Mtb* ATP synthase,^8^ is active against both replicating and dormant *Mtb,* ^9^ and has improved efficacy against intracellular bacteria, which are typically less sensitive to standard TB drugs.^10,11^ Numerous studies have also demonstrated that the ability of bedaquiline to modulate *Mtb* metabolism helps counteract drug resistance mechanisms,^12^ synergizes with existing drugs,^13^ and may enhance the antibacterial activity of host macrophages.^2,7,9,13,14^ Since the approval of bedaquiline in 2012, several other compounds targeting bacterial respiration or bioenergetic pathways, including clofazimine,^15^ and the cytochrome bc1 complex inhibitor telacebec (Q203),^15,16^ have entered clinical trials, and are showing promising efficacy against recalcitrant *Mtb* populations, including nonreplicating bacteria and MDR-TB.^2,9,17^

Unlike other bacteria, *Mtb* is able to simultaneously utilize diverse carbon sources to support growth in vivo.^18^ For example, during infection *Mtb* relies on the metabolism of host-derived fatty acids and cholesterol for energy and biosynthetic building blocks.^19–22^ Specifically, cholesterol metabolites such as acetyl CoA and propionyl CoA are shuttled into the TCA cycle to produce ATP or incorporated into virulence-associated cell wall lipids, repectively.^19^ *Mtb’s* ability to dysregulate sterol homeostasis also modulates the host immune response, producing a more permissive intracellular environment that enables chronic infection.^19,23–25^ Consequently, the development of drugs that inhibit *Mtb* cholesterol metabolism could both decrease *Mtb* fitness by targeting bioenergetic pathways that are required for long-term persistence and support the host immune response.^26,27^

The first step of cholesterol degradation in *Mtb*—C27-oxidation of the cholesterol/enone side chain―is catalyzed by a 48 kDa cytochrome p450 enzyme (P450), CYP125.^28–31^ *Cyp125* (*Rv3545c*) is encoded in the *Mtb i*ntracellular *gr*owth (*igr*) operon,^32^ which is widely conserved across actinomycetes and is essential for *Mtb* survival in macrophages^33^ and mice.^5^ The expression of CYP125 is upregulated during infection or when *Mtb* is cultured in the presence of cholesterol,^25,29^ and *Mtb* CYP125 knockout (Δ*Cyp125)* are unable to grow on cholesterol as the sole source of carbon.^25^ Furthermore, Δ*Cyp125 Mtb* mutants are unable to grow on rich media supplemented with cholesterol, because of the accumulation of the toxic CYP125 substrate cholestenone.^21,25,29,31^ Interestingly, certain strains of *Mtb*, including the common laboratory model *Mtb* H37Rv, express an second P450 enzyme (CYP142) that can oxidize cholesterol and rescues the growth of Δ*CYP125 Mtb*.^25,28,34^ Although the catalytic efficiency CYP125 and CYP142 is similar, they are only distantly related and synthesize the opposite stereoisomers of 26-hydroxycholes-4-en-3-one.^29,34,35^ This partial functional redundancy in the *Mtb* genome highlights the importance of maintaining the integrity of the cholesterol metabolic pathway, and also presents challenges for the development of CYP125/142 inhibitors.

Despite the role of cholesterol metabolism for *Mtb* virulence being identified more than 15 years ago,^19^ there has been little progress in the development of drugs to inhibit this pathway.^36^ Imidazole-containing antifungal drugs (e.g., econazole, clotrimazole), which target the fungal P450 CYP51, bind tightly to the heme-cofactor of several *Mtb* P450s,^28,35–40^ and inhibit the growth of *Mtb.* ^41^ However, the antitubercular activity of these drugs is not dependent on cholesterol, and they have comparatively weak binding affinity to CYP125/142 (*K*_D_ values >1 μM) compared to other essential *Mtb* P450s, which is likely due to the relatively narrow active site channel of the cholesterol oxidases.^28,35^ In addition, the imidazole antifungals are generally not considered suitable for treating TB because of their susceptibility to *Mtb* azole efflux transporters,^42^ and potential to cause adverse side effects and drug–drug interactions.^43^ Our lab previously reported preliminary results from a fragment-based screening campaign targeting CYP125, which yielded several hit fragments that were validated to bind CYP125 by differential scanning fluorimetry (DSF) and ligand-observed NMR.^44^ However, no further optimization of the compounds was attempted because we could not obtain a high quality X-ray crystal structures of ligand-bound CYP125.

Here, we report the development of dual CYP125/142 inhibitors, which inhibit the growth of *Mtb* in extra- and intracellular assays. We initially leverage an efficient biophysical screening strategy to characterize the CYP125/142 fragment binding profile and identify a non-imidazole hit **1a**, which might be more potent, selective, and less susceptible to azole efflux transporters than the antifungal drugs. We subsequently employ CYP142 as a structural proxy to guide hit-to-lead optimization of dual CYP125/142 inhibitors that have low nanomolar binding affinity and inhibit CYP125/142 catalytic activity in vitro. Finally, we demonstrate that these novel CYP125/142 inhibitors have antimicrobial activity against extracellular *Mtb* (including MDR-TB), and *Mtb* in human macrophages. The combination of small molecule inhibitors and structural data reported here, provides a promising step toward the development of chemical probes to study the role of cholesterol metabolism for *Mtb* virulence in vivo, and the development of novel antibiotics to combat MDR-TB. This research has also supported the development of a subsequent series of CYP125 inhibitors with antitubercular activity.^45^

## Results

### Fragment Screening Identifies Preferred CYP125/142 Ligand

A focused library of 80 fragments was assembled in order to characterize the preferred heme-binding chemotype of CYP125 and CYP142, and to identify a common chemical scaffold that could be used for the development of a dual CYP125/142 inhibitor (Table S1).^39^ Each fragment in the library contained an aliphatic or aromatic amine, however, imidazole-containing fragments were specifically deprioritized (≤10%), because of the promiscuity of this functional group for binding to both human and microbial P450s, and sensitivity to azole efflux transporters.^42,46,47^ This library was screened against a panel of purified *Mtb* P450s (Figure 1a), including CYP125 and CYP142, by UV–vis spectroscopy to identify fragments that induced a red-shift in the λ_max_ of the enzymes absorbance spectrum. All P450s have a unique absorbance spectrum that reflects the co-ordination environment of heme iron, and small molecules that coordinate directly to ferric heme using a strong field ligand (e.g., nitrogen) cause a red shift in the λ_max_, which typically indicates stabilization of the inactive, low spin state of the enzyme.^48^ This spectral property makes UV–vis a highly efficient method to identify small molecules with the potential to inhibit P450 activity, and to infer their binding orientation, in the absence of structural data.

**Figure 1:**
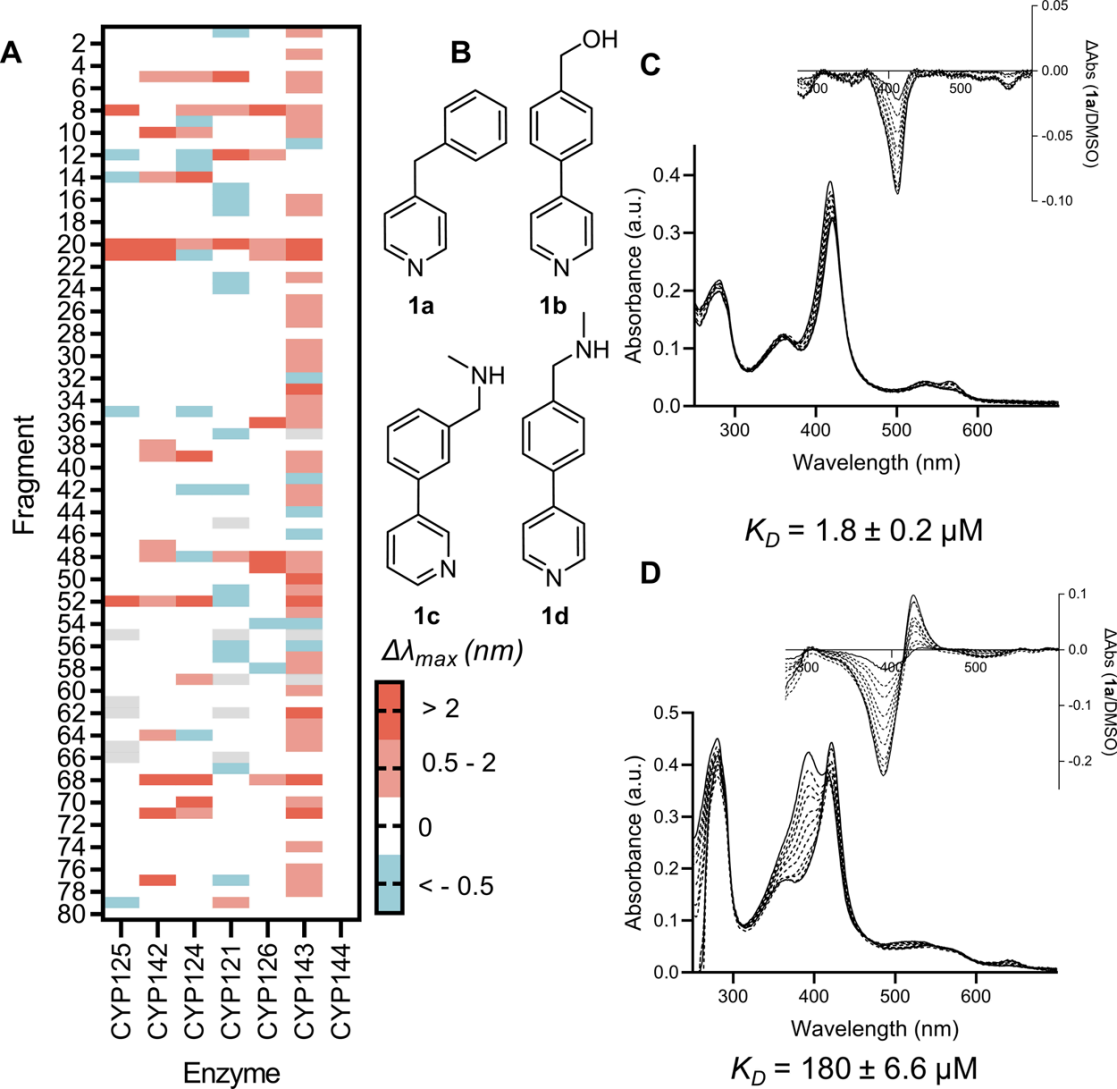
Identification of fragment hit **1a**. (A) Heat map from screening focused fragment library (1 mM) against 7 *Mtb* P450s (4–6 μM) by UV–vis spectroscopy. Color indicates the shift in the maximum wavelength of each enzyme’s absorbance spectrum (Δλ_max_, nm) relative to DMSO. Gray = not tested. (B) Structures of CYP125 hits. (C, D) Absorbance spectra from dose–response titration of fragment **1a** binding to CYP125 (C) or CYP142 (D) (each 5 μM). Insets: Difference spectra verse enzyme-DMSO complex. Data are mean of *n* = 3 titrations. *K*_D_ values were by fitted data to a hyperbolic model.

Only 5% of fragments in the focused library produced a red shift in the λ_max_ of the CYP125 absorbance spectrum, which was surprisingly few compared to other *Mtb* and bacterial P450s that we have previously analyzed.^39,44,49^ All 4 of the CYP125 hit compounds contained a pyridine ring as the putative heme binding motif and a biphenyl or benzylpyridine structure (Figure 1b). In contrast, 15% of fragments in the library produced a red shift in the λ_max_ of the CYP142 spectrum, including 3 of the 4 fragments that were identified as hits for CYP125 (**1a, c, d**). The binding affinity (*K*_D_ value) of fragments **1a**–**d** to CYP125 and CYP142 was determined by optical titration^50^ (Figure 1c,d). Fragment **1a** was found to have low micromolar binding affinity to both CYP125 (*K*_D_ = 180 ± 7 μM) and CYP142 (*K*_D_ = 1.8 ± 2.0 μM); which in addtion to good ligand efficiency (LE = 0.4–0.6), a synthetically tractable chemical structure, and non-azole heme binding group, made **1a** a good candidate for structural characterization and hit-to-lead optimization.

### Structural Characterization of CYP142 Bound to 1a

As CYP125 and CYP142 have a similar biochemical function,^25,28,34,35^ fragment-binding profile,^39^ and binding mode to **1a** (inferred from UV–vis spectroscopy) (Figure 1c,d), we hypothesized that a structure of **1a** in complex with CYP142 might provide a suitable surrogate for CYP125, and help guide the development of dual CYP125/CYP142 inhibitors. A 1.7 Å X-ray crystal structure of **1a** in complex with CYP142 (Figure 2a) was obtained by soaking fragment solutions into CYP142 crystals that were prepared by sitting-drop vapor diffusion. Interestingly, the structure revealed two molecules of **1a** bound per enzyme active site: one coordinated directly to the heme iron via the pyridine-N (**1a**-**i**), and the second located near the entrance of the active site channel (**1a**-**ii**). This CYP142-**1a** structure was aligned with that of CYP125 in complex with econazole (PDB 3IW2),^28^ as this was the only structure available of CYP125 bound to a type II, “inhibitor-like” ligand (Figure 2b). Like **1a**, econazole increases the λ_max_ of the CYP125 (and CYP142) optical spectrum and induces a shift in EPR g-values that is consistent with direct co-ordination of the imidazole ring to ferric heme (as observed for CYP142-**1a**–**i**).^28^ However, as for CYP142-**1a-ii**, electron density for econazole in complex with CYP125 was only resolved near the near the entrance of the active site channel. These similarities made the CYP125-econazole structure suitable for comparison with CYP142-**1a**, and suggested that both heme co-ordination and hydrophobic interactions near the entrance of the P450 active site channel might constitute binding “hotspots”, which could be exploited to optimize the affinity of dual CYP125/142 inhibitors.^51,52^

**Figure 2:**
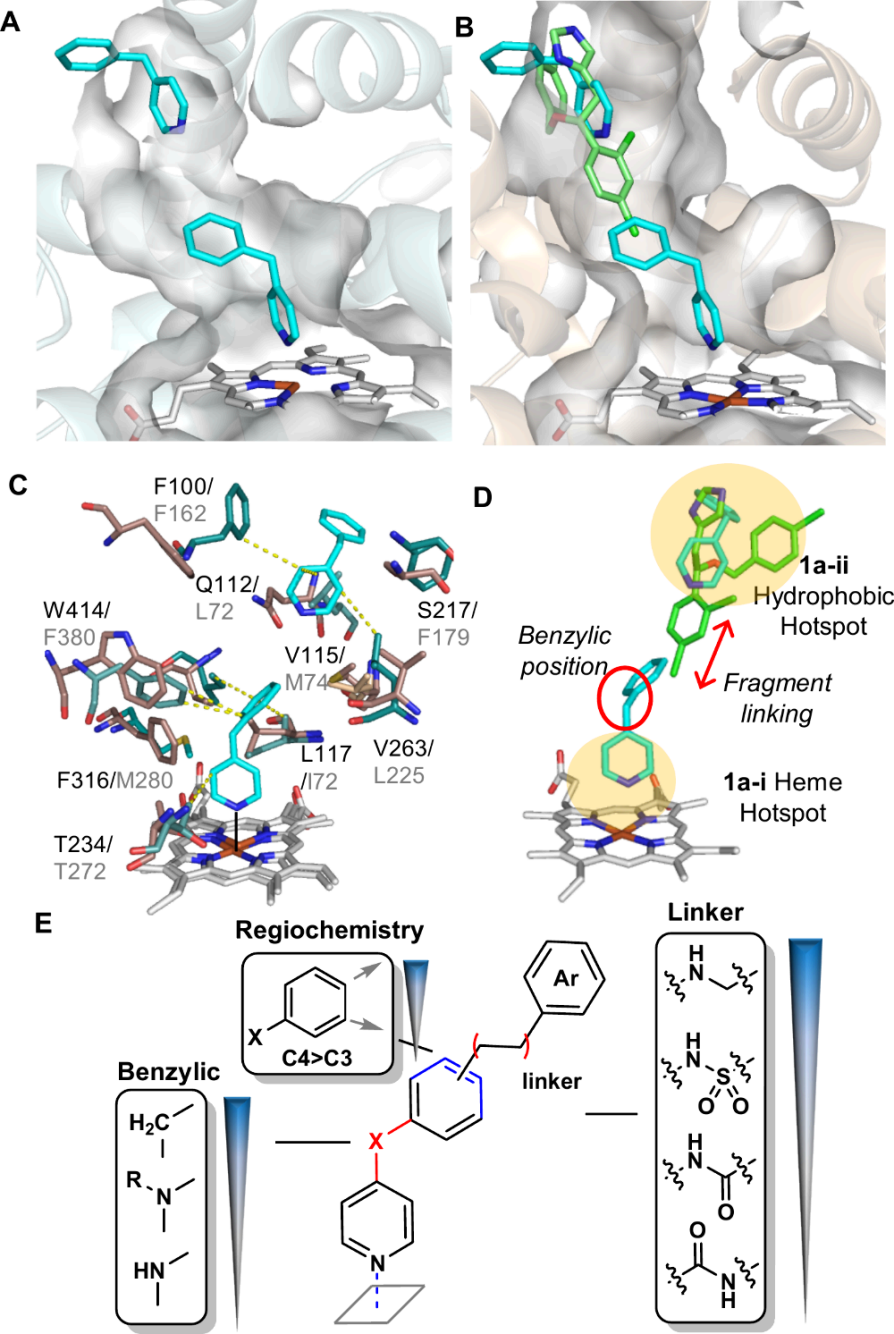
Structure-based design of dual CYP125/142 inhibitors. (A) X-ray crystal structure of the CYP142-**1a** (cyan sticks) complex. Active site surface (gray) (PDB 8S53). (B) Overlay of CYP142-**1a** (cyan sticks) and CYP125-econazole (green sticks) (PDB 3IW2) structures. CYP125 cartoon (wheat) and active site surface (gray). (C) Key interactions (hydrophobic, yellow dashed lines, metal binding, solid gray) between CYP142 (green residues) and **1a**, and comparison with CYP125 active site residues (wheat sticks) Labels reflect CYP125/142 number. Fragment **1a** (cyan). (D) Ligand design strategy, highlighting binding hotspots, and key motifs for SAR exploration. (E) CYP125 SARs established from screening a library of **1a** analogues that contained diverse functional groups at the benzylic position and varied the functional group and substitution pattern of the **1a**-**i**–**1a-ii** “linker” (see Table S2).

The aligned structures indicated that the binding mode of **1a-i/ii** to CYP142 could be accommodated within the conformation of the econazole-bound CYP125 active site (Figure 2b), and enabled the identification of key active site residues that differed between CYP125 and CYP142 (Figure 2c). The most notable of these included replacement of several aromatic residues near the CYP125 heme cofactor and **1a**-**i** benzylic-CH_2_ group (aka “*benzylic position*”), with smaller and/or aliphatic amino acids in CYP142 (e.g., ^125^F316 > ^142^ M280, ^125^W414 > ^142^F380, and ^125^L117 > ^142^I76), and extensive differences in the F/G helices and B–C loop; including substitution of several residues located between the phenyl ring of **1a**-**i** and pyridine of **1a-ii** (aka *“linker”* region) (e.g., ^125^Q112 > ^142^L72, ^125^S217 > ^142^F179, ^125^V115 > ^142^ Met74).^35^ As these variations could produce different SARs, the initial synthetic optimization of a dual CYP125/142 inhibitor focused on generating a library of analogues with diverse substituents at the “*benzylic*” and “*linker*” positions of the **1a** scaffold (Figure 2d).

### Synthetic Optimization of Dual CYP125/142 Inhibitors

A library of **1a** analogues was synthesized and screened by UV–vis spectroscopy to determine the SAR contributing to CYP125/142 binding affinity and selectivity. In the first iteration of compounds (**2a**–**h**), the effect of the “*benzylic*” functional group of **1a**–**i** was analyzed, and in the second iteration (**3a-g, 4a-i**), the functional group and substitution pattern of the “*linker”* used to join the **1a**–**i** phenyl ring with the **1a-ii** hydrophobic hotspot was varied (Figure 2d,e, Table S2). In brief, compounds containing different functional groups at the benzylic position were synthesized by either acid or copper-catalyzed arylation of aniline, phenol, or benzene sulfinic acid with a halopyridine (**2a**–**e**); or the condensation of 4-picoline with benzaldehyde (**2g**–**h**) (Scheme S1). Compounds synthesized to study the SAR of the “linker” were based on the scaffold of either **1a**, or the benzylic amine analogue **2a** (which showed improved binding to CYP142), and incorporated a wide range of functional groups at either C3- or C4- of the phenyl ring (Schemes S2 and S3).

As observed in the original fragment screen (Figure 1a, Table S1), CYP125 SAR were more stringent than CYP142 (Figure 2e, Table S2). For example, only compounds with a tertiary amine (**2b**, **c**) or aliphatic group (**2g**, **h**) at the *benzylic* position caused a significant red shift in the CYP125 λ_max_, while CYP142 additionally bound to benzylic secondary amines (**2a**) or ethers (**2d**). Neither enzyme tolerated a polar group, such as a sulfone (**2e**) or carbonyl (**2f**), at the benzylic position. The addition of a “*linker*” substituent to the phenyl ring of either **1a**–**i**, or the 4-aminophenylpyridine analogue **2a**, typically improved binding, however, SAR were again more stringent for CYP125 than CYP142. Amines (**3d**-**e, 4a**-**b**), amides (**3f**), or sulfonamides (**3g**) derivatives, substituted at C4 of the **1a**–**i** phenyl ring were preferred by CYP125, while CYP142 broadly tolerated amine, ether (**3a**–**b**), and ester (**4c**–**d**) substituents at either C3 or C4, but bound to sulfonamides comparatively weakly (**3g**). Both enzymes disfavored carboxylic acids (**4e**–**f**) or alcohol (**4g**) substituents, but bound more strongly to fragments containing a 3- or 4-bromo substituent (**4h**, **i**), highlighting the potential to significantly improve binding affinity by elaborating the **1a**–**i** scaffold to increase hydrophobic interactions with the **1a-ii** hotspot in the upper active site channel (Figure 2d).

These SAR guided the synthesis of a second generation of **1a** analogues (**5a**–**p**), which were designed to optimize binding affinity for CYP125 and CYP142 by linking together the heme-binding and hydrophobic hotspots accommodated by **1a**–**i** and **1a-ii**, respectively. Each compound contained a methylene or amine at the *benzylic* position, and either an anilide, carboxamide, or sulfonamide *linker* of 3–4 bond lengths. The general synthesis of key compounds in this library is described in Scheme 1. In brief, Suzuki-Miyaura cross-coupling of pyridine boronic acid with a functionalized benzyl chloride (e.g., **7a**–**d**) afforded compounds with a methylene group at the benzylic position and either a carboxamide-linked aromatic group (**5e**, **5f**, **5h**, **5i**) or nitro substituent on the phenyl ring (**3c**). Reduction of the nitro group with tin­(II) chloride yielded primary amines (**3d**, **3e**), which were subsequently coupled with carboxylic acids (**5a**, **5d**) or sulfonyl chlorides (**5j**, **5k**) using carbodiimide chemistry or base, respectively, or functionalized with aromatic substituents by reductive amination with a benzylaldehyde (**5l**, **5m**, **5p**) or acetophenone (**5o**). Compounds with a secondary amine at the benzylic position were synthesized by acid catalyzed coupling of 4-chloropyridine with a functionalized aniline to yield **4c** or **6b**, followed by alkylation with methyl iodide to yield the tertiary amine **8a**. Ester and nitro groups were hydrolyzed or reduced, respectively (**4b**, **4e**, **8b**), and then used to synthesize anilide (**5b**, **5c**), carboxamide (**5g**), or benzylamine (**5n**)-linked aromatic substituents.

**Scheme 1:**
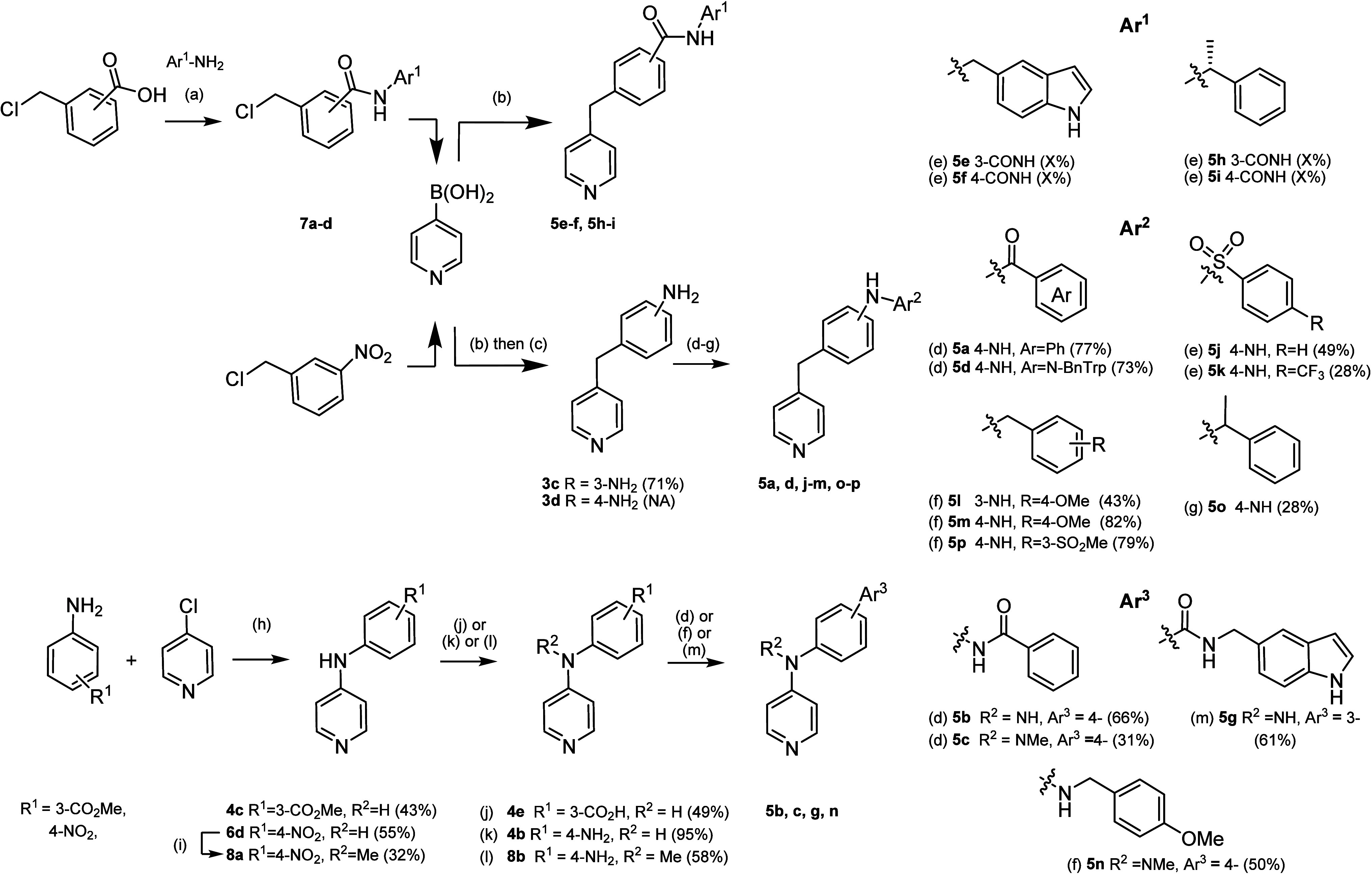
(a) HBTU, Et_3_N, DMC:DMF (7:1), r.t., 24 h; (b) Pd­(PPh_3_)_4_, Na_2_CO_3_, DME:H_2_O (2:1), 100 °C, 4 h; (c) Pd/C, N_2_H_4_.xH_2_O, EtOH, 90 °C, 2 h; (d) ArCO_2_H, HATU, DIPEA, DCM, 0 °C–r.t., 24 h (**5a**, **5b**, **5c**); or PyBOP, NMM, DCM, DMF, r.t., 5 h (**5d**); (e) ArSO_2_Cl, pyridine, r.t., 20 h (**5j**); or ArSO_2_Cl, Et_3_N, DCM, r.t., 20 h (**5k**); (f) RCOH, AcOH, NaCNBH_3_, MeOH, r.t., 20 h (**5l**, **5m**, **5p**, **5n**); (g) RCOMe, TiCl_4_, DCM, 0 °C, 3 h; then Na­(CN)­BH_3_, MeOH, r.t., 24 h (**5o**); (h) HCl (37%), EtOH, 90 °C, 20 h; (i) NaH, DMF, MeI, 0 °C- r.t., 8 h; (j) **4c**, LiOH.H_2_O, MeOH:H_2_O:THF, r.t., 4 h; (k) **6b**, SnCl_2_.2H_2_O, HCl (37%), EtOH, 0-80 °C, 1 h; (l) **8a**, Zn(s), NH_4_Cl, DMF, r.t., 24 h; (m) EDC.HCl, HOAt, DIPEA, DMF:DCM (1:10)

The binding affinity (*K*_D_ values) of the resulting compounds (**2a**, **3f**, **3g**, **5a**–**p**) to CYP125 and CYP142 validated the SARs established during the initial iterations of fragment optimization (Table 1). For example, replacing the benzylic methylene group with a secondary or tertiary amine translated to an approximate 20-fold loss in CYP125 binding affinity (e.g., **5a** vs **5c**, **5e** vs **5g**, **5m** vs **5n**), while varying the C4/C3- substitution pattern on the **1a**–**i** phenyl ring resulted in 10–100-fold difference in *K*_D_ value (e.g., **5e** vs **5f**, **5h** vs **5i**, **5l** vs **5m**). In contrast, the binding affinity of most compounds to CYP142 was similar (*K*_D_ ∼ 1 μM). However, a 20-fold improvement was achieved by replacing either the amide (e.g., **5a**) or sulfonamide (e.g., **5j**) linker with a methyl amine (e.g., **5m**). Combining the SAR favored by CYP125 and CYP142 yielded a potent dual inhibitor **5m**, which had K_D_ values of 40–160 nM for both enzymes, and good ligand efficiency (LE) (>0.4) due to the high group efficiency (GE) of the benzylamine (GE = 0.20–0.50).

**Table 1:**
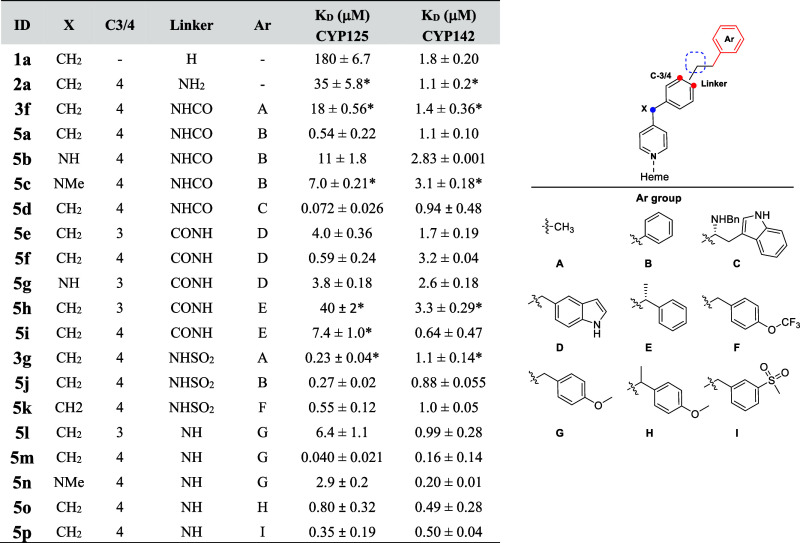
Structure and Binding Affinity of Dual CYP125-142 Inhibitors^a^

a *K*_D_ values were determined by optical titration and are mean values ± SD of *n* = 2–4 titrations, except for values marked (*) where *K*_D_ value ± SD is estimated from fitting *n* = 1 titration.

### Structural Characterization of Dual CYP125/142 Inhibitors

A combination of improvements made to CYP125 expression and crystallization conditions during the synthetic optimization of **1a**, in addition to the tight binding affinity of the dual CYP125/142 inhibitors, enabled us to obtain high resolution X-ray crystal structures of compounds **5j** and **5m** in complex with CYP125 (Figure 3a,c,h) and CYP142 (Figure 3b,d,h) (and compound **5g** in complex with CYP125 (Figures S1 and S2, Table S3)). In all 4 structures, the pyridine-nitrogen of the inhibitor directly coordinated to the P450 heme iron, consistent with their type II optical spectra and the binding mode of **1a**–**i** to CYP142 (Figure 2a). CYP125-**5m** (Figure 3a) and CYP142-**5m** (Figure 3b) structures also illustrate that the 4-methoxybenzylamine substituent accurately recapitulates the binding mode of **1a-ii** in the hydrophobic hotspot (Figure 3e), validating the fragment-linking strategy used to optimize inhibitor binding affinity. In contrast, the conformation of the sulfonamide linker in compound **5j** directs the phenyl substituent away from binding pocket of **1a** **-ii** and introduced disfavorable interactions with CYP142_Met74 (Figure 3c,d,f). This conformation might account for the weaker binding affinity of compounds that contain a sulfonamide (e.g., **3g**, **5k**) or amide linker (e.g., **5a**–**d**) relative to their benzylamine analogues (e.g., **5l**–**p**), and is consistent with the comparatively weak affinity of CYP142 to fragments containing a sulfonamide substituent, which was noted in the original SAR screen (e.g., **3g**, Table S2). Binding interactions between CYP142 and **5m** or **5j** were exclusively hydrophobic (and metal co-ordination), while CYP125 also formed key hydrogen bonding interactions with the amine/sulfonamide linker (**5m**/**5j**) and methoxy group (**5m**) of the ligands via Glu112 and Ser217, respectively. These additional interactions likely contribute to tighter binding affinity and improved inhibition of CYP125 verse CYP142 by **5m** and **5j** (Table 1, Figure 4b, S.I. Table S4).

**Figure 3:**
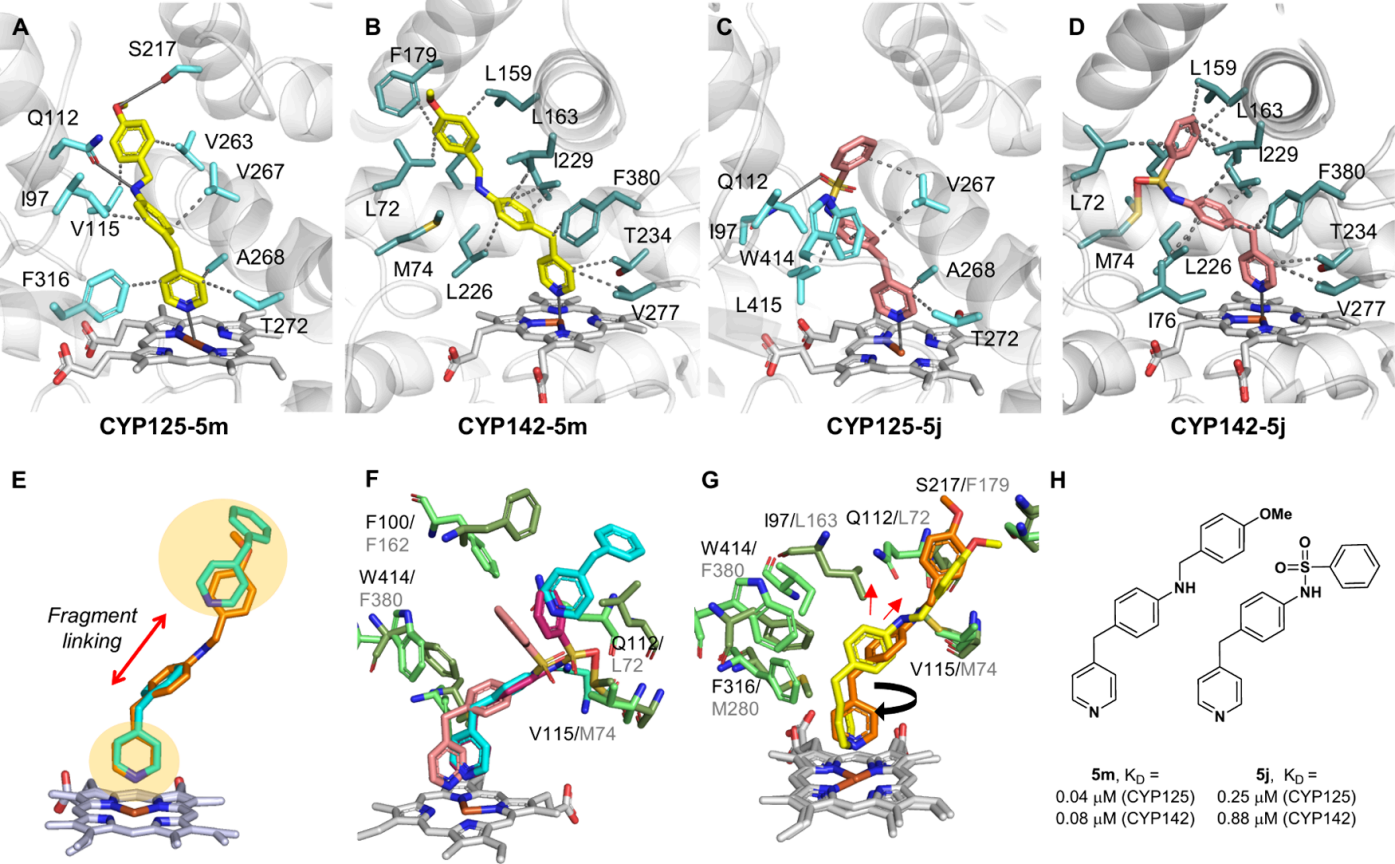
Structural characterization of CYP125–142 inhibitors**.** X-ray crystal structures of (A) CYP125-**5m** (PBD 7ZIC); (B) CYP142-**5m** (PDB 7P5T); (C) CYP125-**5j** (PDB 7ZGL), and (D) CYP142-**5j** (PDB 7QQ7). The heme cofactor (gray sticks), protein (gray cartoon), and key residues (blue/green sticks), hydrophobic (dashes), and hydrogen bonds/metal binding (solid line), are indicated. (E) Overlaid structures of CYP142**-5m** (orange) and CYP142-**1a** (blue) (PDB 8S53), highlighting heme and hydrophobic hotspots (yellow). (F) Overlaid structure of CYP125-**5j** (salmon), CYP142-**5j** (magenta), and CYP142-**1a** (blue), highlighting key active site residues of CYP125 (pale green) and CYP142 (dark green). (G) Overlaid structures of CYP125-**5m** (yellow) and CYP142-**5m** (orange). Black arrow indicates rotated orientation of CYP125-bound pyridine relative to CYP142. Red arrows indicate C3/C4 substitution on phenyl ring. Residues colored as for (E). (H) Chemical structure and K_D_ values of compound **5m** and **5j** binding to CYP125 or CYP142, as determined by optical titration.

**Figure 4:**
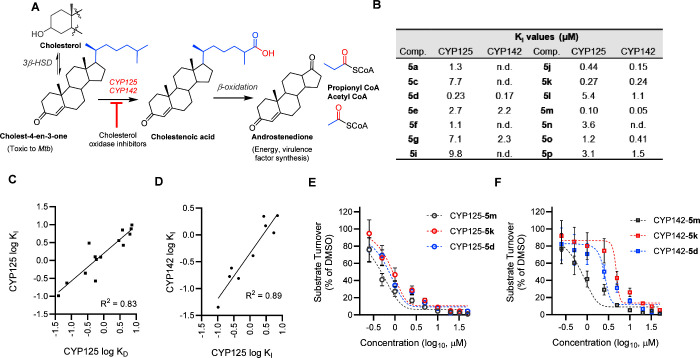
Inhibition of CYP125–142 cholestenone metabolism in vitro. (A) *Mtb* cholesterol metabolism: CYP125 and CYP142 catalyze C27-oxidation of cholest-4-en-3-one to yield cholestenoic acid, which is subsequently degraded to androstendione, acetyl CoA, and propionyl CoA. 3β-HSD—3β-hydroxysteroid dehydrogenase; (B) inhibition equilibrium constants (*K*_I_) values for the turnover of cholest-4-en3-one (5 μM) by CYP125 (0.5 μM) or CYP142 (1 μM). K_I_ values estimated by the Cheng–Prusoff equation, cholest-4–3-one *K*_m_ CYP125 (2.1 μM), K_m_ CYP142 (0.36 μM); (C) binding affinity (*K*_D_) and inhibition constants (K_I_) of **1a** analogues for CYP125 correlates (*R* ^2^ = 0.83, *P* < 0.0001); (D) inhibition (*K*_I_ values) of CYP125 and CYP142 by **1a** analogues correlates (*R* ^2^ = 0.89, *P* = 0.001); (E, F) inhibition of CYP125 (0.5 μM) (E) or CYP142 (1 μM) (F) catalyzed turnover of cholest-4-en-3-one (5 μM) by **5d**, **5k**, and **5m**. Data are mean ± SD of *n* = 3 replicates.

In all CYP125 co-crystal structures, the pyridine ring of the inhibitor was rotated 90 degrees relative to that observed for the same inhibitor in complex with CYP142 (Figure 3g), and the orientation of the **1a**–**i** pyridine ring in the original CYP142-**1a** structure (Figure 2a). This orientation likely optimizes interactions with aromatic residues in proximity to the heme cofactor (e.g., Phe316/M280, Trp414/Phe280 in CYP125/142, repectively, Figure 3g) and contributes to the sensitivity of CYP125 to bulky or polar substituents at the *benzylic* position of **1a**–**i** (Figure 2e, Tables 1 and S2). Rotation of the pyridine ring could also contribute to CYP125’s preference for a C4 substitution pattern on the **1a**–**i** phenyl ring, as unlike CYP142, only elaboration from C4 provides direct alignment with the hydrophobic hotspot.

The structure of compound **5g** in complex for CYP125 revealed a surprising “substrate-like” shift in the enzyme active site, in which residues of the F-, G- and I- helices moved inward toward the heme relative to the apoenzyme (Figure S1a), which is also observed for CYP125 in complex with **cholestenone** (Figure S1b). However, **5g** binding also induced an unusual kink in the I-helix that pushes residues between Val261–267 away, and caused Glu271 to attain a previously unobserved orientation that extends across the heme to accept a hydrogen bond from the benzylic amine of **5g**. In contrast, the previously reported inhibitor- (**econazole**)-bound structure of CYP125 show minimal structural perturbations versus the apoenzyme (Figure S1c).^31^ These unusual structural characteristics could account for the weak type II optical spectra generated by **5g**, and other compounds containing an amine at the benzylic position and/or C3 phenyl substitution pattern.

### Inhibition of CYP125/142 Catalytic Activity In Vitro

The ability of the elaborated **1a** analogues to inhibit CYP125/142 catalytic activity was assessed in vitro using an LC–MS-based substrate turnover assay to monitor the conversion of cholest-4-en-3-one (Figure 4a). Experiments were performed as previously described,^28,35^ using recombinantly expressed and purified CYP125 or CYP142, and an exogenous electron transport chain consisting of spinach ferrodoxin/ferrodoxin reductase coupled to a glucose-6-phosphate/glucose-6-phosphate dehydrogenase-NADP­(H) regenerating system. All compounds that were tested in this assay inhibited CYP125 catalytic activity at concentrations that correlated with the *K*_D_ values determined from optical titrations (*R* ^2^ = 0.82) (Figure 4b,c), and 4 compounds (**5d**, **5j**, **5k**, **5m**) were calculated to have inhibition constants (*K*_I_ values) less than 1 μM (Figure 4b,d, Table S4). A subset of the most potent CYP125 inhibitors was subsequently tested against CYP142 (K_I_ values between 0.05 and 1.1 μM) and found to correlate with the relative potency against CYP125 (*R* ^2^ = 0.89) (Figure 4b–d). The most potent dual CYP125/142 inhibitor **5m** (*K*_I_ = 0.10 μM (CYP125), 0.05 μM (CYP142)), was subsequently selected as the lead candidate for biological profiling. No obvious oxidation of the CYP125/142 inhibitors themselves could be detected in the biochemical assays. However, a detailed analysis of all reaction products was not performed.

### Antimicrobial Activity of Dual CYP125/142 Inhibitors against Extracellular *Mtb*

We initially assessed the antimicrobial activity of the CYP125/142 inhibitors against two different strains of *Mtb* (H37Rv and CDC1551) that were cultured extracellularly on media containing cholesterol as the sole source of carbon, as the genetic disruption of CYP125, or CYP125 and CYP142, inhibits the growth of *Mtb* under these conditions.^21,25,29,35^ The concentration of compound required to completely inhibit *Mtb* growth (MIC_99_) was calculated 2-weeks post-compound treatment from the reduction of resazurin (MABA)^53^ relative to DMSO-treated controls (Tables 2 and S5). The most potent dual CYP125/142 inhibitor **5m**, was found to also have the strongest antimicrobial activity (MIC_99_ = 1.5 μM, ∼0.46 μg/mL, H37Rv *Mtb*). Several other compounds also inhibited *Mtb* growth with modest MIC_99_ values of between 12.5 and 25 μM, including amides **5d**, **5e**, **5g**, sulfonamide **5k**, and benzylamines structurally related to **5m** (**5l**, **5o**, **5p**).

**Table 2:** Antitubercular Activity of CYP125/142 Inhibitors

	cholesterol			mixed media	intracellular growth		MIC_90_(μM)^a^	
	[ATP] IC_50_(μM)^b^		MIC_99_(μM)^c^					
	W1	W2	W2	IC_50_(μM)^d^	*Mtb* IC_50_ (μM)^e^	THP-1 LD_50_(μM)^f^	H37Rv	MDR-TB
**5d**	4.7	4.7	25	18 ± 3	2.8 ± 0.2	45	n.d.	n.d.
**5k**	2.3	2.3	25	22 ± 2	13 ± 0.18	32	n.d.	n.d.
**5m**	0.15	1.1	1.5	2.9 ± 0.19	1.7 ± 0.18	50	0.78	0.39
**Inh.**	<0.1	<0.1	<0.1		0.10 ± 0.01		0.19–0.39	13–25
**pAS**	0.04	0.29	0.19		2.4 ± 0.12			
Moxi.				0.33 ± 0.01				

a The minimum concentration of **5m** to inhibit the growth of multidrug resistant *Mtb* (MDR-TB) (MIC_90_) was determined 1-week post-compound treatment by MABA. Inh—isoniazid, pAS—para-amino salicylic acid; Moxi.—moxifloxacin.

b H37Rv *Mtb* cultured on media containing cholesterol as the sole source of carbon. Inhibition constants (IC_50_) values were estimated 1- and 2-weeks post-compound treatment from the reduction in ATP-dependent luminescence relative to DMSO-treated controls.

c Culture conditions as for (a). The minimum concentration of compound to inhibit 99% *Mtb* growth (MIC_99_) was estimated 2-weeks post-compound treatment using the MABA.

d Inhibition of *Mtb* (Erdman) growth on cholesterol-supplemented (0.01%) media were determined by MABA 1-week post-compound treatment. IC_50_ values were estimated by nonlinear regression, and are mean values ± SD of *n* = 3 replicates. Moxifloxacin IC_50_ < 0.1 μM.

e Inhibition of luciferase-expressing *Mtb* growth in THP-1 macrophages 5-days post-compound treatment. RLU was normalized relative to DMSO treated controls. IC_50_ values were estimated by nonlinear regression and mean values ± SD of *n* = 2 replicates.

f Cytotoxicity of compounds to uninfected THP-1 macrophages. LD_50_ values were estimated by nonlinear regression from the percent reduction in ATP-dependent luminescence relative to DMSO-controls.

Encouraged by these results, we repeated this experiment and used an ATP luminescence assay to provide a more direct measure of the effect of the CYP125/142 inhibitors on *Mtb* metabolism over the 2-week treatment period (Tables 2 and S5). These independent experiments confirmed that **5m** (IC_50_ = 0.15 μM, H37Rv *Mtb*), and benzylamines **5l**, **5o**, **5p** (IC_50_ values 0.15–1.5 μM, H37Rv *Mtb*) potently depleted intracellular ATP concentrations 1-week post-compound treatment (Figure 5a, Table S5). Several other compounds including **5d**, **5g**, and **5k**, also had IC_50_ values <5 μM, which is consistent with the reliance of *Mtb* on cholesterol metabolites to drive ATP generation under these growth conditions,^19^ and parallels the activity of other compounds with target *Mtb* metabolism.^8,9^ The IC_50_ values of all compounds increased between 1-week and 2-week measurements, (e.g., 2-week IC_50_ **5m** = 1.2 μM), suggesting that MIC_99_ values recorded in initial experiments might improve with repeated compound dosing, or measurement 1-week post-compound treatment. Similar SARs were observed for the growth inhibitory effects of the CYP125/142 inhibitors against both H37Rv and CDC1551 strains of *Mtb*, increasing confidence in these experiments. However, MIC_99_ and IC_50_ values were slightly higher for both the CYP125 and FDA-approved inhibitors against *Mtb* CDC1551 (Table S5).

**Figure 5:**
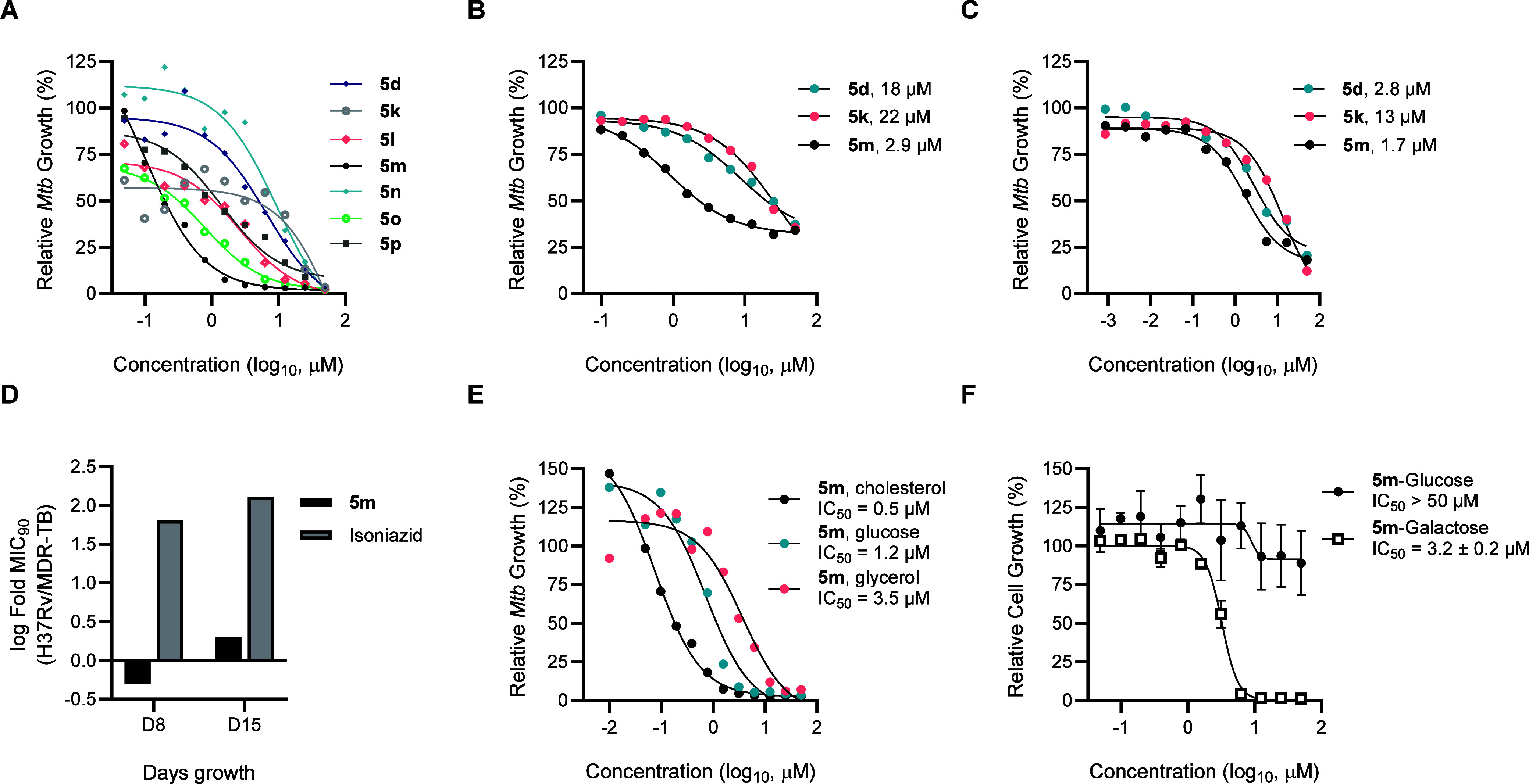
Antitubercular activity of CYP125/142 inhibitors (A) CYP125/142 inhibitors deplete intracellular ATP when *Mtb* (H37Rv) is cultured on cholesterol as the sole carbon source. ATP concentration was assessed 1-week post-compound treatment, and RLU was normalized as a percent of the DMSO-treated control. (B) Inhibition of extracellular *Mtb* (Erdman) growth on media supplemented with 0.01% cholesterol, determined by MABA 1-week post-compound treatment. RFU was normalized as a percent of DMSO-treated controls, and data are mean values ± SD of *n* = 3 replicates. (C) Inhibition of *Mtb* (H37Rv:pATB45luc) growth in THP-1 macrophages, quantified from RLU 5-days post-compound treatment. Data are represented as a percent of the DMSO-treated control are mean values ± SD of *n* = 2 replicates. (D) Lead compound **5m** retains inhibitory activity against multidrug-resistant *Mtb* (MDR-TB). MIC_90_ (μM) values of **5m** and isoniazid were determined against extracellular H37Rv and MDR *Mtb* cultured on standard media 1- and 2-weeks post-compound treatment, and used to calculate log-fold change (MIC_90_ H37Rv/MIC_90_ MDR-TB). (E) Comparison of **5m** potency against extracellular *Mtb* (H37Rv) cultured on media containing either cholesterol, glucose, or glycerol as the sole source of carbon. Inhibition of *Mtb* growth relative to DMSO-treated controls was determined from ATP concentration (cholesterol and glucose) or MABA (glycerol) after 7- or 10-days post-compound treatment, respectively. (F) Selective cytotoxicity of **5m** against HepG2 cells cultured on galactose-containing media. Growth inhibition was determined from ATP concentration (RLU). Data are represented as a percent of the DMSO-treated controls and are mean values ± SD of *n* = 2 replicates.

As the accumulation of the toxic CYP125/142 substrate cholestenone has been shown to inhibit the growth of ΔCyp125/142 *Mtb*,^21^ we also assessed the antimicrobial activity of a representative subset of the CYP125/142 inhibitors (**5d**, **5k**, **5m**) against bacteria that were cultured on standard media supplemented with low concentration cholesterol (0.01% w/v) (Table 2, Figure 5b). For these assays, inhibition of *Mtb* (Erdman) growth was quantified ∼ 1-week post-compound treatment using the MABA. All compounds retained inhibitory activity, with compound **5m** estimated to have an IC_50_ value of 2.9 μM. These data demonstrate that the antimicrobial activity of the CYP125/142 inhibitors could extend to an environment with more diverse nutrient availability, as found in vivo.

### Antimicrobial Activity of CYP125/142 against *Mtb* in Human Macrophages

Encouraged by the activity of the CYP125/142 inhibitors against extracellular *Mtb*, we proceeded to assess the ability of a representative subset of compounds to inhibit the growth of luciferase-expressing *Mtb* (*H37Rv pATB45luc*) in human macrophage-like THP-1 cells.^54^ *Mtb* growth inhibition was determined 5-days post-compound treatment from the reduction in luminescence signal intensity relative to DMSO-treated controls. The potential cytotoxicity of the CYP125/142 inhibitors to uninfected THP-1 macrophages was assessed in parallel experiments using an ATP glow assay. Both benzylamine **5m** (IC_50_ = 1.7 μM) and amide **5d** (IC_50_ = 2.8 μM) potently inhibited the growth of intracellular *Mtb*, while sulfonamide **5k** was considerably less active (13 μM) (Figure 5c, Table 2). Compound **5m** also showed good selectivity over mammalian cytotoxicity, with no effect on THP-1 cell viability at concentrations up to 50 μM. These results illustrate that **5m**, and related benzylamine compounds, have suitable properties for use in cell-based assays, which could help better understand the role of CYP125/142 in *Mtb* pathology.

### CYP125/142 Inhibitor Activity Against MDR-TB

Finally, we assessed the ability of benzylamine **5m** to inhibit the growth of a MDR strain of *Mtb* (K26b00MR 113), which is insensitive to the first line anti-TB drugs isoniazid and rifampicin.^55^ The antimicrobial activity of **5m** and isoniazid were assessed in parallel against drug sensitive H37Rv *Mtb* and MDR-TB by MABA, 1- and 2-weeks post-compound treatment. While **5m** retained a similar MIC_90_ value against both strains of *Mtb* (H37Rv = 0.78 μM, MDR-TB = 0.39 μM), the IC_50_ value of isoniazid was >50–150-fold higher against MDR-TB (Table 2, Figure 5d, Table S6). Considering the increasing prevalence of MDR-TB across the globe, and limited pipeline of novel anti-TB drugs, the potency of **5m** in these experiments supports further exploration of the benzylpyridine chemotype as anti-TB agents.

### Biological Mechanism and Safety Profiling

Confident that **5m** inhibits the growth of *Mtb* on cholesterol, we subsequently explored the scope of the compound’s antimicrobial activity by testing a subset of the CYP125/142 inhibitors against extracellular *Mtb* cultured on other defined carbon sources. These experiments revealed a notable 8-fold decrease in the potency of **5m** against *Mtb* grown on glucose (IC_50_ = 1.2 μM) compared to cholesterol (IC_50_ = 0.15 μM), and >20-fold decrease in potency against *Mtb* grown on minimal media supplemented with glycerol (IC_50_ = 3.5 μM) (Figure 5e, Table S7). Similarly, there was a 2–16-fold increase in the IC_50_ value of benzylamines **5l**, **5o**, and **5p**, and sulfonamide **5k** against *Mtb* grown on glucose verse cholesterol, while in contrast, compound **5d** had a similar IC_50_ value regardless of media composition (Table S7).

The carbon-source dependent antimicrobial activity of **5m** is consistent with a mechanism of action that is, at least in part, dependent on inhibiting cholesterol metabolism. However, these results also indicated that the compound may have an additional mechanism(s) of action. A panel of reporter assays were subsequently used to detect whether the compound interacted with biological pathways targeted by existing TB drugs (Figure S3). As treatment of *Mtb* with compound **5m** did not induce the upregulation of *iniB*, or *recA* and *radA* reporters, our results imply that the compound is unlikely to inhibit *Mtb* cell wall synthesis, or induce DNA damage, respectively.^56^ However, data from several other reporter assays were inconclusive due to the presence of BSA (Figure S4) and further mechanistic characterization should be performed in future.

The good selectivity of **5m** for bacterial cytotoxicity verse mammalian cells that was observed when the compound was tested against THP-1 macrophages (Table 2), was independently verified in experiments using HepG2 cells cultured on standard (glucose-containing) media (LD_50_ > 50 μM). However, notably, **5m** showed significant cytotoxicity against HepG2 cells cultured on media containing galactose (LD_50_ = 3.2 μM), indicating that the compound may inhibit oxidative metabolism (Figure 5f).^57^

Finally, to provide insight into whether CYP125/142 inhibitors based on the benzylpyridine scaffold might cause drug–drug interactions or off-target activity when used in vivo, **5m** was screened against a panel of human drug metabolizing P450s (Table S8). As several P450 isoforms were inhibited as concentrations <5 μM, the compound has potential to cause drug–drug interactions and further optimization of the benzylpyridine scaffold to improve CYP125/142 selectivity would be required for in vivo applications. Despite this, the good activity of **5m** against intracellular *Mtb* and MDR-TB, and low mammalian cytotoxicity, should make the compound a useful tool to study CYP125/142 in cell-based assays, and promising that further optimization could yield a novel class of anti-TB compounds.

## Discussion and Conclusions

The reliance of intracellular *Mtb* on host-derived cholesterol for long-term survival and virulence, makes cholesterol metabolism a compelling biological target for the development of novel antibiotics.^19–22,26,27,58^ Despite this, few compounds have been developed to specifically inhibit key enzymes involved in *Mtb* cholesterol metabolism.^59–61^ Here, we have described an efficient fragment- and structure-guided approach to develop small molecule inhibitors of the P450 enzymes CYP125 and CYP142, which catalyze the first committed step of cholesterol degradation in *Mtb*.^28–31,34,35^ The lead compounds developed in this study have activity against both intracellular *Mtb* and MDR-TB, low toxicity to human macrophages, and drug-like chemical properties, which should make them useful tools to study *Mtb* sterol metabolism, and are promising step toward the development of novel drugs that could help combat the global TB pandemic.

Our earlier attempts to develop CYP125 inhibitors were hindered by a lack of structural data to guide hit-to-lead optimization,^44^ and further complicated by the discovery that CYP142—an enzyme with low sequence or structural similarity to CYP125—could rescue the growth of Δ*Cyp125 Mtb* on cholesterol.^25,34,35^ To overcome these technical and biological challenges, we leveraged fragmented screening by UV–vis spectroscopy to efficiently sample chemical space,^62^ characterize the SARs shared by CYP125 and CYP142, and identify a chemical scaffold with suitable drug-like properties that could be used for the development of a dual CYP125/142 inhibitor. By designing a tailored heme-binding fragment library and employing UV–vis spectroscopy, instead of more commonly used biophysical screening techniques,^63^ we were able to rapidly identify fragments that not only bound to CYP125 and CYP142, but also functionally stabilized the low-spin or inactive state of the enzymes (Figure 1a–d).^48^ UV–vis spectroscopy also provided detailed understanding of the binding site and orientation of the fragment hits, which helped guide hit-to-lead optimization chemistry, and enabled us to use the co-crystal structure of **1a** in complex with CYP142 as a structural proxy for CYP125 (Figure 2b,d).

The significant improvement in binding affinity that was achieved through synthetically linking together fragment **1a**-**i** and **1a-ii** to yield compound **5m** (*K*_D_ **1a**/**5m**: CYP125 > 1000-fold, CYP142 > 10-fold), illustrates how fragment screening can be used to identify energetic hotspots that may contribute disproportionately to binding affinity.^64^ Furthermore, as observed previously, our data highlights the importance of optimizing the properties of the chemical linker to ensure that the original fragments can maintain an optimal binding orientation (Figure 3a–d, and Table 1).^65,66^ The lead compound development through this fragment-linking approach (**5m**) binds to both CYP125 and CYP142 with comparable affinity to the enzyme’s endogenous substrates (*K*_D_ ∼ 100 nM),^34,35^ has excellent ligand efficiency (LE > 0.4), and potently inhibits the enzyme’s catalytic activity in vitro (*K*_I_ ∼ 0.05–0.10 μM).

Throughout the fragment screening and inhibitor optimization campaign we noted that SARs for CYP125 were significantly more sensitive than CYP142. For example, varying the substitution pattern or chemistry of the **1a**-**i**–**1a-ii** linker resulted in up to 100-fold difference in CYP125 binding affinity, while CYP142 *K*_D_ values varied <10-fold (Table 1, Figure 2e). These results reflect the structural differences in upper active site of CYP125 and CYP142,^35^ and support hypothesis that CYP142 may have evolved to metabolize a more diverse pool of sterol substrates than CYP125.^67^ While CYP125 is encoded within the conserved *igr* operon, and has been functionally characterized as a cholesterol oxidase in multiple species of actinobacteria,^27–30^ CYP142 is encoded within a cluster of lipid metabolizing genes and shares greater structural similarly with *Mtb* CYP124;^35^ an enzyme that is thought to primarily oxidize fatty acids and vitamin D.^68,69^ Building on the data reported here, could help develop isoform-selective CYP125 or CYP142 inhibitors that would facilitate research into the enzyme’s independent roles in *Mtb* sterol metabolism. For example, the significantly larger proportion of CYP125-**5m** binding affinity that can be attributed to forming interactions with the **1a-ii** hotspot (ΔΔG [**5m**-**1a**] = 0.5) compared to CYP142 (ΔΔG [**5m**-**1a**] = 0.2), suggests that hydrophobic interactions near the entrance of the P450 active site contribute disproportionality to CYP125 ligand recognition. In contrast, CYP142 binding affinity to the **1a** compound series appears to be more strongly driven by pyridine-heme co-ordination. Removing or attenuating the potency of heme binding pyridine might favor CYP125 selectivity or could be used to reduce off-target interactions with other P450s, while modifying the linker to exploit differences in the distal active site of CYP125/142 might yield CYP142-selective compounds. The ligands and SAR reported here may also help guide the development of inhibitors for the human cholesterol oxidases CYP27A1 and CYP46A1, both of which currently lack chemical probes. As CYP27A1 and CYP46A1 play important roles in bile acid biosynthesis and the elimination of cholesterol from the brain, respectively,^70–72^ profiling the activity of the dual CYP125/142 inhibitors against human CYP27A1 and CYP46A1, as well as a broad spectrum of other human P450s, will be an important consideration when assessing their further optimization as anti-TB compounds.

The antimicrobial activity of the dual CYP125/142 inhibitors against *Mtb* grown on cholesterol (Figure 5a, Tables 1 and S4), or cholesterol-supplemented rich media (Figure 5b, Table S5), is consistent with previous studies in which either *Cyp125*, or *Cyp125* and *Cyp142*, were genetically disrupted.^21,25,29,31^ In addition, the weaker activity of **5m**, and related benzylamine compounds, against *Mtb* that was cultured on either glucose or glycerol as a sole source of carbon supports a mechanism of action that is, at least in part, dependent on cholesterol utilization (Figure 5e, Table S7). In contrast, the potency of control compounds (e.g., 4-aminosalicylic acid, isoniazid) was similar regardless of media composition. Although nutrient availability can alter *Mtb* growth rate, we did not observe any intrinsic differences in fitness across experimental conditions, and the MIC values of control compounds (e.g., 4-aminosalicylic acid, isoniazid) was similar regardless of carbon source (Tables S5 and S7).

Despite this, the ability of **5m** to inhibit the growth of extracellular *Mtb* in the absence of cholesterol, suggests that either CYP125 and CYP142 have important, uncharacterized physiological functions, or that **5m**, and related analogues, have a secondary mechanism of action. Preliminary biological profiling indicated that **5m** is unlikely to induce DNA damage or inhibit *Mtb* cell wall synthesis, which are the mechanisms of some existing first-line TB drugs (e.g., isoniazid, fluoroquinolones), however, other reporter assays were inconclusive (Figure S3). The ability for both bedaquiline and **5m** to potently decrease intracellular ATP, and the enhanced potency of both compounds against *Mtb* grown on lipids compared to standard glucose or glycerol media, suggests an overlap in their mechanism(s) of action at the level of oxidative phosphorylation.^7,73^ However, further mechanistic characterization is required. Despite this, the potent activity of **5m** against both drug susceptible and MDR- *Mtb* cultured under a variety of conditions provides promise that further optimization of the benzylpyridine scaffold could yield compounds that retain antitubercular activity in vivo, where *Mtb* can access more heterogeneous carbon sources.

*Mtb’s* unique metabolic adaptions to survive in human macrophages contributes to the bacteria’s reduced sensitivity to first-line TB drugs, and the need to identify compounds which specifically have activity against intracellular *Mtb*.^7,11^ As the utilization of cholesterol is required to establish a long-term, chronic infection, and is one of the primary nutrients available to non-replicating *Mtb,* ^3,19,21,74^ we anticipate that drugs targeting CYP125/142 could help to specifically address recalcitrant bacterial populations. Our study demonstrates that compound **5m** inhibits the growth of *Mtb* in recently infected human macrophage-like cell lines, and we anticipate that, like other drugs targeting *Mtb* metabolism, **5m** may also have activity against dormant *Mtb.* The antitubercular activity of compound **5d** against intracellular *Mtb* reflects the relative difference in *K*_D_ values of **5m** verse **5d** (∼2 fold) for CYP125, and was significantly better than the antitubercular activity observed for **5d** against extracellular *Mtb*. This could be due to physicochemical properties or modulation of the host-cell environment. However, as **5d** was weakly cytotoxic to THP-1 cells, the significance of these data should be interpreted with caution.

The low cytotoxicity of compound **5m** to both THP-1 macrophages and HepG2 cells cultured on glucose is consistent with evidence that THP-1 cells are primarily glycolytic,^75^ and should enable the compound to be used as a chemical tool to help study the role of CYP125/142 during infection. In addition, as shifting macrophage metabolism toward aerobic glycolysis correlates with a more effective immune response,^76^ **5m** might synergistically decrease *Mtb* fitness by inhibiting CYP125/142 and modulating host-cell metabolism.^77^ For example, hydroxycholesterol metabolites, such as those synthesized by CYP125/142, have been reported to polarize macrophages toward a more tolerogenic M2 phenotype.^78,79^ As such, it would be intriguing in future studies to analyze whether CYP125/142 inhibition alters macrophage cytokine profiles. Furthermore, as carbon liberated from cholesterol metabolism is used to synthesize virulence-associated lipids such as phthiocerol dimycocerosate,^19,20^ future studies should evaluate the effect of CYP125/142 inhibition on *Mtb* cell wall integrity and immunogenicity.

In contrast, compound **5m** was selectively cytotoxic to HepG2 cells cultured on galactose media, suggesting that the compound might inhibit mammalian mitochondrial function.^57^ Interestingly, the FDA-approved *Mtb* ATP-synthase inhibitor bedaquiline has also been shown to inhibit the growth of tumor-initiating cancer stem cells by interfering with mitochondrial function.^80^ As such, determining the potential mammalian targets of the CYP125/142 inhibitors is also important for future research.

Many drug discovery campaigns that are initiated from a target-centric or in vitro approach fail due to a lack of cellular activity, often as a result of inadequate drug permeability or susceptibility to efflux.^81,82^ Our approach attempted to address these challenges from the outset by screening a tailored fragment library that was biased away from azoles, which are common efflux substrates,^42,43^ and by selecting a ligand efficient hit fragment with a distinct structure to existing drugs.^83^ The good antitubercular activity of **5m** against both extra- and intracellular *Mtb* suggests that the compound is able to adequately penetrate both mammalian cells and the complex mycobacterial cell wall, however, a direct analysis of intracellular exposure was not performed (Table 2, Figure 5). Furthermore, we anticipated that like bedaquiline, and other compounds that deplete cellular ATP, **5m** should inherently decrease *Mtb* efflux transporter activity, thus potentially increasing the efficacy of other antimicrobial drugs.^7,12^

In summary, we have reported an efficient fragment-based approach to develop the first cell active dual CYP125/142 inhibitors. The potency of these compounds against priority *Mtb* populations, including intracellular and MDR bacteria, low toxicity toward human macrophages, and distinct chemical scaffold from existing compounds are promising for their further optimization as chemical tools or antibiotics. The antitubercular activity of the CYP125/142 inhibitors exemplifies that expanding the scope of biological pathways considered for drug development offers potential to develop antibiotics with new mechanisms of action. In this respect, (host)-microbial metabolism is ripe with potentially druggable targets that await exploitation.

## Experimental Section

### Safety Statement

All experiments using strains H37Rv, Erdman, and CDC1551, and luciferase-modified variants, carry some risk of infection and were performed using appropriate safety protocols in BSL3 certified laboratories. The protocols described herein do not pose a high risk for aerosolization.

No other unexpected or unusually high safety hazards were encountered in chemical or biological methods.

### Protein Expression and Purification

All experiments, except for the crystal structures obtained of CYP125A1 in complex with inhibitors **5g**, **5j**, and **5m**, and CYP142A1 in complex with inhibitor **5j** and **5m**, were performed using *Mtb* CYP125A1 and *Mtb* CYP142A1 proteins that were expressed and purified as previously described.^28,35^ In brief, *Cyp125A1* (*Rv3545c*), encoding residues 1–433, and *Cyp142A1* (*Rv3518c*), encoding residues 1–398, were expressed as N-His_6_-tagged constructs from pET15b vectors in C41­(DE3) cells. Bacteria were cultured in 2xYT medium supplemented with ampicillin (100 mg/L) at 37 °C until an OD600 of 0.8. The temperature was then reduced to 23 °C and isopropyl ß-D-1-thiogalactopyranoside (150 μM) was added to induced protein expression, along with 5-aminolevulinic acid (100 μM) to enhance heme incorporation. Bacteria were cultured for a further 18–24 h and then harvested by centrifugation (9000 g, 4 °C, 20 min) and stored at −80 °C until purification. Cell pellets were thawed on ice, resuspended in 50 mM potassium phosphate buffer (pH 8.0), containing 250 mM KCl, 10% v/v glycerol, DNase, lysozyme, protease and phosphatase inhibitors (cOmplete EDTA-free protease inhibitor cocktail tablets, Roche (1 tablet/50 mL), PMSF (1 mM), and benzamidine hydrochloride (1 mM)), and lyzed by sonication. Supernatants were clarified by centrifugation (40,000 g, 4 °C, 30 min) and then purified by His-tag affinity chromatography (Ni-NTA (Qiagen) or HisTrap FF (GE Healthcare) eluting with up to 55 m imidazole (CYP142A1) or 200 mM imidazole (CYP125A1). Protein containing fractions were pooled and dialyzed overnight into 50 mM Tris–HCl, pH 7.2, containing 1 mM EDTA and 50 mM KCl, and then purified by anion exchange chromatography (Resource-Q or Q-sepharose, GE Healthcare), eluting with 50–500 mM KCl. Protein containing fractions were pooled and dialyzed into 50 mM Tris–HCl, pH 7.2, containing 1 mM EDTA, concentrated, and purified by gel filtration chromatography (Sephacryl S-200, GE Healthcare). Protein purity and concentration was determined by SDS–PAGE and UV–visible spectroscopy, using the previously established extinction coefficients for CYP125 (ε_449–490_ = 91 mM^–1^ cm^–1^)^29^ and CYP142 (ε_418_ = 140 mM^–1^ cm^–1^),^35^ then aliquots were snap frozen and stored at −80 °C until further use.

Crystal structures of CYP125A1 and CYP142A1 in complex with lead compounds **5g**, **5j**, and **5m** were generated using N-terminally truncated constructs. Cyp125A1 (Rv3545c), encoding residues 18–433, and Cyp142A1 (Rv3518c), encoding residues 2–398, were cloned into a pET21a vector downstream of an T7 leader sequence, and a TEV-cleavable Twin-Strep hexa-histidine dual affinity tag. Proteins were expressed as described above, except that media was supplemented with 250 μM 5-aminolevulinic acid and 200 μM IPTG. Harvested cells were lysed by sonication in 50 mM potassium phosphate pH 8.0, 200 mM KCl, 10% v/v glycerol, supplemented with protease and phosphatase inhibitors, DNase, and lysozyme, and then clarified by centrifugation (42,000 × g, 4 °C, 1 h). Proteins were purified from supernatants by gravity affinity chromatography using Strep-Tactin XT high capacity resin (IBA Lifesciences), eluting with buffer supplemented with 1x BXT Strep-Tactin elution buffer. The Twin-Strep His_6_-tag was removed by overnight incubation with Tobacco etch virus protease (TEV) (1:20, TEV:P450), followed by incubation with Nickel-NTA (Qiagen) or Nickel-EXCEL (GE Healthcare) resin for 1 h. The tag-free protein was collected by gravity filtration, concentrated to 1 mL, and purified by size exclusion chromatography, typically using a Superdex 200 (GE Healthcare) column equilibrated with 20 mM HEPES or Tris-HCl buffer, pH 7.5, containing 200 mM KCl, and 1 mM TCEP. Protein purity and concentration was determined as above (S.I. Figure 5), and either directly used in crystallography experiments, or flash frozen.

### Compound Screening by UV–Visible Spectroscopy

Compounds (1–100 mM) were prepared as stock solutions in *d*_6_-DMSO and added as a 2 μL aliquot to solutions of P450 proteins (4–6 μM, 198 μL), or to buffer alone, to achieve a final concentration 1–100 μM compound and 1% v/v *d*_6_-DMSO. Samples were either analyzed in quartz cuvettes with a 1 cm path length using a CARY400 UV–vis spectrophotometer (Varian, U.K.), or in UV-star 96-well microplates (Greiner Bio-one, U.K.) using a CLARIOstar microplate reader (BMG Labtech, Germany) in absorbance mode. Spectra were recorded continuously between 800 and 250 nm at 25 °C. Spectra of the compound in buffer alone were subtracted from protein-containing spectra to account for any inherent UV absorbance of the small molecule. Difference spectra were generated by subtracted the spectrum of an inhibitor-free protein sample from test samples. The magnitude of change in the maximum wavelength of the Soret band of the enzyme’s absolute absorbance spectrum (λ_max_) relative to a DMSO control (CYP125 λ_max_ = 392.5 nm, CYP142 λ_max_ = 418 nm), and the change in absorbance between the maximum and minimum wavelengths of the enzymes difference spectrum (ΔAbs), were used to identify P450 ligands. Spectral perturbations that caused a red-shift in the enzyme Soret band (λ_max_) was typically classified as Type-II, “inhibitor-like,” while those that caused a blue shift were classified as Type I, “substrate-like” interactions.^48,84,85^ As CYP125 is predominantly high spin (HS) at resting state,^28^ Δλ_max_ was calculated for both the HS and low spin (LS) enzyme populations represented in the spectra, and the LS/HS ratio was used to further evaluate the degree of LS stabilization, or “inhibition”. Perturbations of Δλ_max_ < ±1 nm using the CARY400 spectrophotometer, or <±1.5 nm using the CLARIOstar microplate reader were considered within experimental error. All UV–vis spectra were generated using Origin software (OriginLab, Northampton, MA) or MARS Data Analysis Software (BMG Labtech). Data were processed using Microsoft Excel (Microsoft Office, 2010).

### Optical Titrations to Determine Dissociation Constants

Optical titrations were performed using a Varian Cary 400 UV–vis spectrophotometer (Varian, CA, USA) according to a previously described procedure.^38^ Assays were performed in reduced volume (200 μL) quartz cuvettes with a path length of 1 cm (Starna, Essex, U.K.). Ligands were prepared as *d*_6_-DMSO stock solutions (0.25–500 mM) and proteins (4–6 μM) were prepared in the appropriate buffer. Aliquots (0.2 μL) of ligand stock solutions were added directly to cuvettes containing either protein solutions, or buffer alone. The final *d*_6_-DMSO concentration did not exceed 1% v/v of the assay solution. Spectra were recorded between 800 and 250 nm at 25 °C after the addition of each aliquot of ligand. Buffer control spectra were subtracted from protein spectra to account for any inherent absorbance of added ligands/solvent. Difference spectra were generated by subtracting the initial ligand-free protein spectrum from each successive titration spectrum. The maximum change in absorbance for each difference spectrum was then plotted against ligand concentration and fitted using a one-site binding model hyperbolic/Michaelis–Menten equation (eq 1), the Hill function for cooperative binding (eq 2) or a modified version of the Morrison equation (eq 3) for tight binding inhibitors.^86^

$$A_{\mathrm{obs}}=(A_{\max}\times L)/(K_{D}+L)$$ 1

$$A_{\mathrm{obs}}=(A_{\max}\times (L{)}^{n})/((K_{D}{)}^{n}+(L{)}^{n})$$ 2

$$A_{\mathrm{obs}}=(A_{\max}\times 2\mathrm{Et})\times ((L+\mathrm{Et}+K_{D})-(((L+\mathrm{Et}+K_{D}{)}^{2})-(4\times L\times Et){)}^{0.5})$$ 3

In eqs 1–3, *A*_obs_ is the observed change in absorbance, *A*_max_ is the maximum absorbance change at saturation, Et is the enzyme concentration, *L* is the concentration of ligand, *n* is the extent of cooperativity and *K*_D_ is the dissociation constant for the P450-ligand complex. Data were processed using Microsoft Excel (Microsoft Office, 2013). Data fitting and analysis were performed using Origin software (OriginLab, Northampton, MA) or GraphPad Prism 5.01 (GraphPad Software, San Diego, USA).

### X-ray Crystallography

The CYP142A1-**1a** structure was obtained using the N-His_6_-tagged construct. All other structures were obtained using the truncated, tag-free constructs of CYP125A1 and CYP142A1. Crystallization was performed using the sitting-drop vapor diffusion method, at 15–20 mg/mL protein, using a Mosquito nanolitre pipetting robot (TTP labtech). Crystals of CYP125 were obtained in 0.1 M MES buffer, pH 6.5, containing 1.5–2.1 M ammonium sulfate. Crystals of CYP142A1 used compound **5j** and **5m** were obtained in 0.1 M sodium acetate, pH 4.5, containing 0.1 M potassium bromide, 8% PEG 20,000, and 8% PEG 550 MME. Compounds were prepared as saturated DMSO stocks and diluted with crystallization mother liquor to 2.5% of total volume. These soaking solutions were pipetted onto drops containing crystals at 4 °C for at least 24 h, then crystals were harvested, cryoprotected using paratone oil, and frozen in liquid nitrogen for data collection. The structure of CYP142A1 in complex with fragment **1a** was obtained using CYP142A1 (15 mg/mL) crystallized in 0.1 M sodium acetate, pH 4.8, containing 0.1 M potassium thiocyanate, 8% PEG 200 and 10% PEG 550 MME. Crystals were back-soaked with 24% PEG 550 MME to remove PEG200 and then soaked with 4 mM fragment **1a**. Diffraction data sets were collected at Diamond light source in Oxfordshire at various beamlines. Data was integrated using the DIALS pipeline,^87^ with scaling and merging performed using aimless.^88^ Crystallographic models were solved using molecular replacement using the published ligand-free enzyme structures (3IW0 for CYP125 and 2XKR for CYP142). Model building was performed using COOT^89^ with ligand restraints generated using ACEdrg.^90^ Refinement was performed using PHENIX.refine.^91^ Data tables and statistics are provided in S.I. Table 3, and ligand density maps are in S.I. Figure 1. All structures have been deposited in the Protein Data Bank (http://www.rcsb.org/pdb/) under the accession codes: CYP142-**1a** (8S53), CYP142-**5j** (7QQ7), CYP142-**5m** (7P5T), CYP125-**5j** (7ZGL), CYP125-**5m** (7ZIC), CYP125-**5g** (8S4M). Images of crystal structures were generated using an academic version of the PyMOL Molecular Graphics System, Version 1.3, 2010, Schrödinger, LLC.

### Substrate Turnover Assay

Substrate turnover and inhibition assays were set up using either CYP125 (0.5 μM) or CYP142 (1 μM), 10 μM spinach ferredoxin and 1.5 μM spinach ferredoxin reductase in 50 mM potassium phosphate buffer, pH 7.5, containing 150 mM KCl, and 0.05% Tween-20 (potassium phosphate buffer). Cholest-4-en-3-one (10 mM) was prepared in 45% (v/v) HPCD, and compound stock solutions were diluted in DMSO to 25–100 mM. CYP-ferredoxin mixtures were preincubated with cholest-4-en-3-one (5 μM) and compounds (0–100 μM) for 30 min at 25 °C, and then substrate turnover was initiated by the addition of an NADPH regeneration system consisting of 1 mM NADPH, 10 mM glucose-6-phosphate and 2 U glucose-6-phosphate dehydrogenase in potassium phosphate buffer. Reactions were allowed to proceed for between 0 and 45 min with shaking at 750 rpm at 30 °C, then quenched by the addition of an equal volume of acetonitrile, followed by shaking at 900 rpm for 10 min. Samples were then filtered through protein precipitation plates (Phenomenex) under vacuum into mass spectrometry plates. Turnover of cholest-4-en-3-one was monitored by LC-MS using an Agilent 6545XT Advance Bio LC/Q-TOF, equipped with a 2.1 × 100 mm, 1.8 μM Agilent Eclipse Plus C18 column and an elution gradient of 0.1% formic acid in water to 0.1% formic acid in acetonitrile. Samples were quantified with reference to an androstenedione internal standard and a cholest-4-en-3-one calibration curve. Reactions were performed at a range of substrate concentrations, with 5 μM being selected as optimal for calculating IC_50_ values for this compound series. Control reactions were also performed in the absence of NADPH. Data (*n* = 3) were analyzed using Agilent MassHunter Quantification software and resulting IC_50_ curves were fitted in OriginLab graphing software. IC_50_ values were converted to K_I_ values using the Cheng-Prusoff Equation, (cholestenone *K*_m_ CYP125 = 2.1 μM, *K*_m_ CYP142 = 0.36 μM).

### Inhibition of Extracellular *Mtb* (H37Rv) Growth on Defined Carbon Sources

 (*Mtb*) H37Rv or CDC1551 strains were grown in Middlebrook 7H9 broth medium (Difco) supplemented with 0.3 g/L casitone, 0.81 g/L NaCl, 0.05% (v/v) tyloxapol, and either 97 mg/L cholesterol, 4 g/L glucose, or 0.2% glycerol. For inhibitor assays, a 10-fold serial dilution of the test compounds was made in the desired medium in duplicate rows of a 96-well plate. *Mtb* cells were then added to all the wells at the final concentration of 1 × 10^4^ CFU, and plates were incubated at 37 °C for up to 15 days. Minimum inhibitory concentrations (MIC_99_) were determined using the Microplate Alamar Blue Assay (MABA) on day 15. In brief, resazurin reagent (1:10 dilution of Alamar Blue reagent, Invitrogen) was added to the MIC plates and the cultures were incubated for 24 h at 37 °C. The concentration of compound required to completely inhibit resorufin fluorescence was determined visually.

Depletion of intracellular ATP concentration by 50% (IC_50_) values were determined as described above except that measurements were made on day 8 and day 15. BacTiter Glo reagent (Promega) was added to microtiter plates (1:10 dilution) and luminescence was recorded after 15 min of incubation at room temperature. IC_50_ values were determined to be the concentration of compound required to reduce luminescence by 50% relative to DMSO-treated controls. Isoniazid or *p*-amino salicylic acid were used as positive control compounds for all experiments. Reported MIC and IC_50_ values are the mean of duplicate treatments.

### Inhibition of Extracellular MDR-TB Growth

MDR-TB strain K26b00MR 113, which is resistant to isoniazid and rifampicin, was cultured on glucose-casitone media (as described above) and treated with DMSO, compound **5m** (50–0.05 μM), or isoniazid. *Mtb* growth was monitored on day 7 and day 14 post-compound treatment by MABA and is reported as the minimum concentration required to inhibit 90% growth (MIC_90_). Assays were performed in duplicate and data are mean values.

### Inhibition of Extracellular *Mtb* (Erdman) Growth

*Mtb* (Erdman) was maintained in Middlebrook 7H9 broth medium containing 2% v/v glycerol, 5% w/v BSA, 2 g/L dextrose, and 3 mg/L catalase; supplemented with 2% w/v glucose. Three days prior to the assay, the Erdman strain was pre-adapted to cholesterol by switching the glucose containing 7H9 media to that supplemented with 0.01% w/v cholesterol. Cultures were treated with DMSO or compounds (50–0.098 μM), and growth inhibition was assessed 7 days post-compound treatment by the addition of resazurin. Plates were incubated for 48 h and then fluorescence was recorded. Assays were performed in triplicate and all plates contained moxifloxacin (0.005–2.5 μM) treated controls, which corresponded to 100% inhibition of *Mtb* growth. Percent growth in each well was calculated relative to maximum signal intensity in uninhibited wells, and IC_50_ values were estimated by nonlinear regression (3-parameter), using GraphPad Prism v10.0.1.

### HepG2 Cytotoxicity Assay

Compounds were tested against HepG2 cells for their ability to inhibit ATP production as a measure of cytotoxicity. HepG2 cells were cultured in DMEM supplemented with 10% (v/v) FBS, Hepes, l-glutamine and glucose or galactose. Compounds were serial diluted in microtiter plates (as described above) and HepG2 cells were added at a final concentration of 20,000 cells per well. Inhibition of ATP levels was noted by adding CellTiter Glo (Promega) reagent at a 1:10 dilution and luminescence was noted after 10 min incubation at room temperature.

### Mechanisms of Action Reporter Assays

Assays to determine compound mechanisms of action were performed using bioluminescent transcriptional reporter *Mtb* strains as described previously.^56^ In brief, compounds were prepared as a 2-fold serial dilution in 96-well plates (50–0.05 μM) and *Mtb/iniB, Mtb/recA,* or *Mtb/radA* (27572410) was added to final concentration of 1 × 10^6^ cells per well. The plates were incubated at 37 °C for 1 week and luminescence was recorded on days 1, 2, 4, and 7 post-compound treatment. Signal intensity was normalized as a % of the maximum signal intensity induced by control compounds which inhibit cell wall synthesis (SQ109, top concentration = 100 μM), or induce DNA damage (moxifloxacin, top concentration = 25 μg/mL), respectively. Assays were performed in duplicate, and data are shown as mean values ± SD.

### Intracellular Growth Assay

Intracellular growth assays were performed as previously described,^54^ with minor modifications. *Compounds—*Compounds were prepared as DMSO stock solutions and 50 μL was dispensed into assay plates as an 11-point 3-fold serial dilution using a HP Dispenser D300e Control, V3.3.1, Device 2.69.0.0 (Tecan). The final DMSO concentration was 0.5% and all compounds were tested in duplicate.

#### THP-1 Cell Culture

THP-1 cells (ATCC TIB-202) were maintained in RPMI-1640 (Sigma R5886), supplemented with 10% FBS (FBS SOUTH AMERICAN (CE), Gibco #10270,), 1 mM sodium pyruvate (Sigma, #S8636) and 2 mM l-glutamine (Sigma, #G2150) at 37 °C, 5% CO_2_, and 95% humidity. Cells were handled according to GSK policies for management of human biological samples.

#### Preparation of Mtb Single Bacteria Suspensions

The luminescent strain *Mtb* H37Rv pATB45luc grown at 37 °C in Middlebrook 7H9 medium (Difco) supplemented with 0.2% glycerol, 0.5% bovine albumin fraction, 0.2% dextrose, 0.003% catalase (Becton Dickinson), 0.05% tyloxapol (Merck). Hygromycin B was added to the medium at a final concentration of 50 μg/mL. All experimental work with live *Mtb* H37Rv was carried out following standard operating procedures in compliance with Biosafety Level 3 regulations (BSL3). A single bacteria suspension of *Mtb* H37Rv pATB45luc was prepared prior to infection. Twenty-five mL of bacterial culture grown to OD_600_ = 0.6 (log phase in our conditions is between OD_600_ from 0.05 to 1) was centrifuged at 2230*g* for 10 min. After removal of the supernatant, bacteria were dispersed by vigorously shaking with sterile glass beads 4MM (201-0278 VWR) for 2 min. Dispersed bacteria were then resuspended in 35 mL of RPMI medium and left to decant for 5 min at room temperature. Thirty mL of the supernatant were centrifuged at 308*g* for 5 min. Supernatant was collected and its OD_600_ was measured. OD/mL was converted to CFU/mL (OD_600_ 0.125 ∼ 10^8^ CFUs/mL).

#### Infection of THP-1 Cells with Mtb

1 × 10^6^ THP-1 cells were simultaneously differentiated with phorbol myristate acetate (PMA, 40 ng/mL, Sigma, #P1585) and infected with a single cell suspension of *Mtb* H37Rv pATB45luc in a roller bottle at a MOI of 1:10. Cells were incubated for 4 h at 37 °C at 1.5 rpm. After incubation, infected cells were washed five times by centrifugation at 308*g* for 5 min to remove extracellular bacilli and resuspended in fresh RPMI medium. In the last wash, a Falcon cell strainer 40 μm (Corning) was used to remove cells clumps. The infected cells were resuspended in RPMI medium supplemented with 10% FBS, 2 mM l-glutamine and 1 mM sodium pyruvate at a concentration of 2 × 10^5^ cells/mL. 50 μL of this cell suspension (10,000 cells) were dispensed into 384-well plates containing compounds. Plates were incubated at 37 °C, 5% CO_2_ and 90% relative humidity for 5 days. On day 5, luminescence, which is proportional to bacterial load, was determined by using the BrightGlo Luciferase Assay System (Promega, # E2650) according to the manufacturer’s protocol, except that 20 μL of Bight-Glo mix was used instead of 50 μL. Plates were read using an Envision Multilabel Plate Reader (PerkinElmer) using the 384-plate Ultra-Sensitive luminescence mode, with a measurement time of 200 ms per well. All plates were assayed in duplicate.

#### Data Analysis

All assay plates contained a DMSO-treated column which correspond to 100% bacterial growth, and a Rifampicin (5 μM) treated column, which corresponds to 100% inhibition of *Mtb* growth, which were used to assess assay quality (*Z′* ≥ 0.4) and to normalize data on a per-plate basis. Growth (%) for each well was calculated relative to the maximum signal intensity in the uninhibited samples. IC_50_ values for each compound was estimated by nonlinear regression (3-parameter) using GraphPad Prism 10.0.01

### THP-1 Cytotoxicity

THP-1 cells (ATCC TIB-202) were maintained in RPMI-1640 (Sigma R5886), supplemented with 10% FBS (FBS SOUTH AMERICAN (CE), Gibco #10270,), 1 mM sodium pyruvate (Sigma, #S8636) and 2 mM l-glutamine (Sigma, #G2150) at 37 °C, 5% CO_2_, and 95% humidity. For cytotoxicity assays, THP-1 monocytes were seeded at 5 × 10^5^ cells/mL and treated with 40 ng/mL phorbol myristate acetate (PMA) (Sigma, # P1585) to differentiate for 4 h. The cells were then harvested, washed with complete medium, adjusted to 2 × 10^5^ cells/mL, and 50 μL (10,000 cells) was transferred to each well of clear-bottomed, sterile 384-well plates (Greiner, #781095), which already contained 250 μL/well of DMSO/compounds diluted in media. Diluted compounds were prepared as a 12-point serial dilution (1:3) from a top concentration 50 μM. Compounds were assayed in duplicate in each assay plate. Each assay plate contained a column of DMSO negative controls which correspond to 100% growth, and a column of doxorubicin (Sigma, #D1515) positive controls which correspond to 100% inhibition of growth, which were used to monitor assay quality (*Z*′ > 4). Hygromycin (Sigma) was then added at the final concentration of 0.1 mg/mL, and the plates were incubated for 5 days at 37 °C, 5% CO2 and 95% humidity. Luminescence was measured on day 5 using the ATPLite 1-step kit (PerkinElmer, #6016739). Briefly, 25 μL of reconstituted substrate solution was added to each well, the plate was shaken for 1 min in the dark, and then luminescence was measured using EnVision Multilabel Reader (PerkinElmer), using measurement time 0.1 s. Percent growth inhibition was calculated as %Inhibition = 100 × [(data – DMSO)/(doxorubicin – DMSO)]. The concentration of the compound necessary to inhibit 50% of THP-1 cell growth (LD_50_) and was calculated by fitting %inhibition data by nonlinear regression (GraphPad Prism).

## Chemical Synthesis and Characterization

### General Methods

All reagents were commercially sourced unless otherwise specified. All reactions were conducted under the positive pressure of a dry nitrogen atmosphere. Anhydrous solvents were either freshly distilled (DCM and MeOH over CaH_2_, THF over CaH_2_ and LiAlH_4_) or purchased from commercial sources. Reactions were monitored by liquid chromatography mass spectrometry (LCMS) or thin layer chromatography (TLC), using Merck glass-backed silica (Kieselgel 60 F254 0.25 mm) plates. TLC plates were visualized under UV (254/365 nm) and retention factors (*R*_f_) are provided for the noted solvent system. Flash column chromatography was performed using an IsoleraTM Spektra One/Four purification system and either a GraceResolv LOK flash cartridge containing silica gel (40 μm) (Grace Discovery Sciences, USA) or Biotage SNAP column containing KP-silica gel (50 μm). Solvents are reported as volume/volume (v/v) eluent mixture. Proton (^1^H) and carbon (^13^C) nuclear magnetic resonance (NMR) spectra were recorded at 300 K using either a Bruker 400 MHz AVANCE III HD Smart Probe, 400 MHz QNP cryoprobe or 500 MHz DCH cryoprobe spectrometer. Chemical shifts are given in parts per million (ppm) (δ), relative to residual protonated solvent peak of the deuterated solvent indicated, and the relative integral, multiplicity, and coupling constants (*J* Hz) of the peaks is noted. Assignment of ^1^H NMR and ^13^C NMR spectra was assisted by DEPT, and 2D NMR experiments (COSY, edited ^1^H–^13^C-HSQC and 1H–13C HMBC) where necessary. Infrared (IR) absorption spectra were recorded on a Spectrum One FT-IR (PerkinElmer) spectrometer by attenuated total reflectance (ATR). Data are reported as vibrational frequency (ν_max_, cm^–1^) and peak intensity—strong (s), medium (m), weak (w) or broad (br). LCMS was carried out using an AQUITY UPLC H-class system (Waters, Manchester U.K.). Samples were either run under acidic conditions on an ACQUITY UPLC HSS C-18 column, eluting with a gradient of 95–5% v/v water (containing 0.1% formic acid) in MeCN, or under basic conditions on an ACQUITY UPLC BEH130 C18 column, eluting with a gradient of 95–5% v/v water (containing 10 mM NH_4_OAc) in MeCN over a period of 3.5 min. Small molecule high resolution mass spectrometry (HRMS) was carried out using a Micromass Quadrapole-Time-of-flight (Q-Tof) mass spectrometer, Waters Xevo G2-XS QTof mass spectrometer or a ThermoFinnigan Orbitrap Classic LCMS spectrometer attached to a Dionex Ultimate 3000 HPLC. The mass to charge ratio (*m*/*z*) of the molecular ion and difference from calculated mass (δ ppm) have been quoted. All final compounds used in protein binding or cell-based assays had a purity of >95% by LCMS analysis.

### *N*-Phenylpyridin-4-amine (**2a**)

Aniline (273 μL, 3.00 mmol) was added to a solution of 4-chloropyridine hydrochloride (450 mg, 3.00 mmol) in EtOH (15 mL), followed by a catalytic amount of conc. HCl (37%, 4 drops), and the reaction was heated at 90 °C for 20 h. The reaction was then concentrated to approximately 7 mL and quenched with saturated NaHCO3. The aqueous phase was extracted with EtOAc (4 × 20 mL) and the organic fractions were combined, dried over anhydrous Na2SO4 and the solvent was removed under reduced pressure. The crude product was purified by flash chromatography (0–10% v/v MeOH in DCM) to yield compound 2a as a white solid (286 mg, 1.58 mmol, 53%). *R*_f_ 0.07 (10% v/v MeOH in DCM); ^1^H NMR (400 MHz, CDCl_3_) δ 8.26 (d, *J* = 6.5 Hz, 2H), 7.36 (app. t, *J* = 7.9 Hz, 2H), 7.20 (d, *J* = 7.6 Hz, 2H), 7.13 (t, *J* = 7.4 Hz, 1H), 6.83 (d, *J* = 6.6 Hz, 1H), 6.47 (br s, 1H) ppm; ^13^C NMR (100 MHz, CDCl_3_) δ 151.0, 150.0, 139.6, 129.7, 124.4, 121.8, 109.6 ppm; IR (solid) *v*_max_ 3054–2700 (w, br, N–H), 2896, 2838 (w, C–H), 1610 (m, C = C), 1585 (s, pyridine CC/CN), 1524 (m, C=C, N–H), 1491 (s, N–H), 1448 (m, C=C), 1349 (m, C–N), 1334 (s, C–N), 1236 (w, C–H), 1217 (m, C–H), 994 (s, C–H), 893 (w, C–H), 806 (m, C–H), 749, 694 (s, C–H), 638 (w, C=H) cm^–1^; LCMS (+ESI) *m*/*z* 171.1 [M + H]+, retention time 1.73 min, (100%); HRMS (+ESI) *m*/*z* (Calcd C_11_H_11_N_2_ [M + H]^+^, 171.0917), *Obs*. 171.0913 (δ 2.1 ppm).

### *N*-Methyl-*N*-phenylpyridin-4-amine (**2b**)

Potassium *tert-*butoxide (53 mg, 0.47 mmol) was added as a single portion to a solution of *N*-phenylpyridin-4-amine **2a** (20 mg, 0.12 mmol) in anhydrous DMF (3 mL) at room temperature. Methyl iodide (30 μL, 0.47 mmol) was added and the reaction was stirred at room temperature for 19 h, before being diluted with water (5 mL). The product was extracted into Et_2_O (3 × 10 mL), the combined organic fractions were dried over anhydrous Na_2_SO_4_ and then the solvent was removed under reduced pressure. The crude product was purified by flash chromatography (0–10% v/v MeOH in DCM) to yield compound **2b** as a yellow amorphous solid (17 mg, 0.09 mmol, 75%). ^1^H NMR (500 MHz, CDCl_3_) δ 8.17 (d, *J* = 6.3 Hz, 2H), 7.44–7.40 (m, 2H), 7.27 (dd, *J* = 7.4, 1.2 Hz, 1H), 7.19 (d, *J* = 7.4 Hz, 2H), 6.54 (dd, *J* = 5.1, 1.5 Hz, 2H), 3.31 (s, 3H) ppm; ^13^C NMR (125 MHz, CDCl_3_) δ 154.3, 149.1, 146.1, 130.3, 126.9, 126.8, 108.4, 39.7 ppm; IR (solid) *v*_max_ 3035, 2921, 1642, 1604, 1584, 1493, 1361, 1224, 985, 808 cm^–1^; LCMS (+ESI) *m*/*z* (Calcd C_12_H_13_N_2_ [M + H]^+^, 185.1), *Obs.* 185.2, retention time 1.37 (100%).

### *N,N*-Diphenylpyridin-4-amine (**2c**)

Diphenylamine (217 mg, 1.3 mmol), Pd­(OAc)_2_ (11 mg, 0.05 mmol), *rac-*BINAP (31 mg, 0.05 mmol) and potassium *tert-*butoxide (321 mg, 2.9 mmol) were added to a stirred suspension of 4-bromopyridine hydrochloride (207 mg, 1.1 mmol) in toluene (9 mL). The reaction was heated at 70 °C for 16 h, then cooled to room temperature and the product was extracted into Et_2_O (20 mL). The combined organic fractions were washed with brine (3 × 30 mL), dried over anhydrous Na_2_SO_4_ and the solvent was removed under reduced pressure. The crude product was purified by flash chromatography (0–15% v/v EtOAc in DCM) to yield compound **2c** as a brown solid (15 mg, 0.06 mmol, 6%). ^1^H NMR (400 MHz, CDCl_3_) δ 8.21 (d, *J* = 6.2 Hz, 2H), 7.34 (app. t, *J* = 7.9 Hz, 4H), 7.26–7.15 (m, 6H), 6.72 (dd, *J* = 5.0, 1.5 Hz, 2H) ppm; ^13^C NMR (100 MHz, CDCl_3_) δ 153.9, 150.4, 145.4, 130.0, 126.9, 125.8, 113.0 ppm; IR (solid) *v*_max_ 2988, 2902, 1575, 1483, 1450, 1338 1075, 812, 694 cm^–1^; LCMS (+ESI) *m*/*z* (Calcd C_17_H_15_N_2_ [M + H]^+^, 247.1), *Obs.* 247.0.

### 4-Phenoxypyridine (**2d**)

Phenol (141 mg, 1.5 mmol), copper powder (6.4 mg, 0.1 mmol) and Cs_2_CO_3_ (1.0 g, 3 mmol) were added to a stirred suspension of 4-chloropyridine hydrochloride (150 mg, 1 mmol) in anhydrous DMF (2.2 mL). The reaction was placed under an inert atmosphere are heated to 100 °C for 18 h, then allowed to cool to room temperature and diluted with DCM (20 mL). The solution was washed with 1 M NaOH (40 mL) and water (35 mL), then dried over anhydrous Na_2_SO_4_ and the solvent was removed under reduced pressure. The crude material was purified by flash chromatography (0–5% v/v MeOH in DCM) to yield compound **2d** as a brown solid (6.2 mg, 0.04 mmol, 4%). ^1^H NMR (400 MHz, CDCl_3_) δ 8.46 (m, 2H), 7.43 (app. t, *J* = 7.9 Hz, 2H), 7.26 (t, *J* = 7.4 Hz, 1H), 7.10 (d, *J* = 8.4 Hz, 2H), 6.84 (d, *J* = 8.4 Hz, 2H) ppm; ^13^C NMR (100 MHz, CDCl_3_) δ 165.1, 154.2, 151.5, 130.4, 125.7, 121.0, 112.4 ppm; IR (solid) *v*_max_ 3054, 2920, 2850, 1598, 1572, 1497, 1485, 1264, 1210, 990, 821 cm^–1^; LCMS (+ESI) *m*/*z* (Calcd C_11_H_10_NO [M + H]^+^, 172.1) *Obs.* 172.3 [M + H]^+^, retention time 0.71 min (100%).

### 4-(Phenylsulfonyl)­pyridine (**2e**)

4-Iodopyridine (205 mg, 1 mmol), benzenesulfinic acid sodium salt (197 mg, 1.2 mmol), *l* -proline sodium salt (27 mg, 0.2 mmol) and CuI (19 mg, 0.1 mmol) were combined in DMSO (3 mL) and the reaction was stirred at 80 °C for 24 h. An addition equivalent of l-proline sodium salt (27 mg, 0.2 mmol) and CuI (19 mg, 0.1 mmol) were then added, and the reaction was heated for a further 19 h, before being allowed to cool to room temperature. The reaction was then diluted with water (20 mL), washed with brine (20 mL), the organic fraction was dried over anhydrous MgSO_4_ and then the solvent was removed under reduced pressure. The crude product was purified by flash chromatography (0–50% v/v EtOAc in DCM) to yield compound **2e** as an off-white solid (5.1 mg, 0.02 mmol, 2%). ^1^H NMR (400 MHz, CDCl_3_) δ 8.81 (d, *J* = 6.0 Hz, 2H), 7.95 (d, *J* = 7.5 Hz, 2H), 7.75 (dd, *J* = 4.5, 1.6 Hz, 2H), 7.63 (dd, *J* = 7.5, 1.6 Hz, 1H), 7.54 (app. t, *J* = 7.5 Hz, 2H) ppm; ^13^C NMR (100 MHz, CDCl_3_) δ 151.4, 150.0, 140.0, 134.4, 129.9, 128.4, 120.8 ppm; IR (solid) *v*_max_ 3082, 2922, 2850, 1572, 1475, 1450, 1404, 1324, 1158, 1112, 739 cm^–1^; LCMS (+ESI) *m*/*z* (Calcd C_11_H_10_NO_2_S [M + H]^+^, 220.0) *Obs.* 220.2 [M + H]^+^.

### (*E*)-4-Styrylpyridine (**2g**)

Benzaldehyde (1.12 mL, 11 mmol) was added to a stirred solution of 4-picoline (0.98 mL, 10 mmol) in acetic anhydride (10 mL). The reaction was heated under reflux (140 °C) for 2 h and the solvent was removed under reduced pressure. Iced water (30 mL) was added to the resulting residue and the mixture was brought to pH 1 using 10% HCl solution. The mixture was washed with DCM (5 × 25 mL) and then the aqueous fraction was neutralized using 2.5 M NaOH. The product was extracted into DCM (5 × 20 mL) and then the combined organic fractions were passed through a short plug of silica, eluting with DCM (100 mL). The solvent was then removed under reduced pressure to yield compound **2g** as a yellow solid (710 mg, 3.9 mmol, 39%). ^1^H NMR (400 MHz, CDCl_3_) δ 8.58 (d, *J* = 5.6 Hz, 2H), 7.52 (d, *J* = 7.5 Hz, 2H), 7.41–7.23 (m, 6H), 7.00 (d, *J* = 16.4 Hz, 1H) ppm; ^13^C NMR (100 MHz, CDCl_3_) δ 150.1, 144.7, 136.1, 133.3, 128.9, 128.8, 127.0, 126.0, 120.9 ppm; IR (solid) *v*_max_ 3025, 1719, 1635, 1589, 1549, 1495, 1455, 1414, 971, 808 cm^–1^; LCMS (+ESI) *m*/*z* (Calcd C_13_H_12_N [M + H]^+^, 182.1) *Obs.*183.1 [M + H]^+^, retention time 1.38 min (100%).

### 4-Phenethylpyridine (**2h**)

Pd/C (10% wt., 5 mg) was added to a solution of (E)-4-styrylpyridine (X) (50 mg, 0.28 mmol) in EtOH (5 mL) and the reaction as stirred under an atmosphere of *H*_2_(*g*) for 20 h. When no starting material remained, the reaction was filtered through a short plug of Celite and the solvent was removed under reduced pressure to yield compound **2h** as an off-white solid (48 mg, 0.26 mmol, 94%). ^1^H NMR (400 MHz, CDCl_3_) δ 8.46 (d, *J* = 6.0 Hz, 2H), 7.29–7.11 (m, 5H), 7.06 (d, *J* = 6.0 Hz, 2H), 2.91 (m, 4H) ppm; ^13^C NMR (100 MHz, CDCl_3_) δ 150.7, 149.9, 140.9, 128.7, 128.6, 126.5, 124.2, 37.3, 36.8 ppm; IR (solid) *v*_max_ 3029, 2923, 2858, 1716, 1595, 1558, 1493, 1452, 1410, 1068, 989, 807 cm^–1^; LCMS (+ESI) *m*/*z* (Calcd C_13_H_14_N [M + H]^+^, 184.1) *Obs.* 184.2 [M + H]^+^, retention time 1.38 min (100%).

### 4-(3-Methoxybenzyl)­pyridine (**3a**)

4-Pyridinylboronic acid (73 mg, 0.6 mmol), 3-methoxybenzyl chloride (68 μL, 0.50 mmol), and Na_2_CO_3_ (111 mg, 1.05 mmol) were combined in a microwave vial and flushed with argon. Dry THF (2 mL) and water (1 mL) were added, followed by Pd­(PPh_3_)_4_ (11 mg, 0.01 mmol) and the reaction was further flushed with argon. The vial was sealed and heated at 100 °C for 1 h in a in a microwave reactor. The reaction was cooled to room temperature and diluted with water (5 mL) and DCM (5 mL). The phases were separated and the aqueous phase was extracted with DCM (3 × 5 mL). The organic fractions were combined, dried over anhydrous Na_2_SO_4_ and the solvent was removed under reduced pressure. The crude material was purified by flash chromatography (0–5% v/v MeOH in DCM) and the product containing fractions were combined to yield compound **3a** as a yellow oil (57.9 mg, 0.29 mmol, 58%). ^1^H NMR (400 MHz, CDCl_3_) δ 8.48 (dd, *J* = 5.9, 1.6 Hz, 2H), 7.23 (dd, *J* = 8.2, 7.5 Hz, 1H), 7.10 (m, 2H), 6.77 (m, 2H), 6.70 (dd, *J* = 2.1 Hz, 1H), 3.93 (s, 2H), 3.77 (s, 3H) ppm; ^13^C NMR (100 MHz, CDCl_3_) δ 159.9, 150.0, 149.9, 140.5, 129.8, 124.3, 121.5, 115.0, 111.9, 55.3, 41.3 ppm; LCMS (+ESI) *m*/*z* 200.1 [M + H]^+^, 3.47 min, (95%); HRMS (+ESI) *m*/*z* (Calcd C_13_H_14_NO [M + H]^+^, 200.1070), Obs. 200.1068 (δ 1.2 ppm).

### 4-(4-Methoxybenzyl)­pyridine (**3b**)

4-Pyridinylboronic acid (73 mg, 0.6 mmol), 4-methoxybenzyl chloride (68 μL, 0.50 mmol), and Na_2_CO_3_ (111 mg, 1.05 mmol) were combined in a microwave vial and flushed with argon. Dry THF (2 mL) and water (1 mL) were added, followed by Pd­(PPh_3_)_4_ (11 mg, 0.01 mmol) and the reaction was further flushed with argon. The vial was sealed and heated at 100 °C for overnight (conventional). The reaction was cooled to room temperature and diluted with water (5 mL) and DCM (5 mL). The phases were separated, and the aqueous phase was extracted with DCM (3 × 5 mL). The organic fractions were combined, dried over anhydrous Na_2_SO_4_ and the solvent was removed under reduced pressure. The crude material was purified by flash chromatography (0–5% v/v MeOH in DCM) and the product containing fractions were combined to yield compound **3b** as a colorless oil (25.2 mg, 0.13 mmol, 25%). ^1^H_NMR (400 MHz, CDCl3) δ 8.48 (m, 2H), 7.08 (m, 2H), 6.85 (m, 2H), 3.90 (s, 2H), 3.79 (s, 3H) ppm; ^13^C NMR (100 MHz, CDCl3) δ 158.5, 150.6, 149.9, 131.0, 130.1, 124.2, 114.2, 77.5, 77.2, 76.8, 55.4, 40.4 ppm; LCMS (+ESI) *m*/*z* 200.1 [M + H]^+^, 3.43 min, (100%); HRMS (+ESI) *m*/*z* (Calcd C_13_H_14_NO [M + H]^+^, 200.1070), Obs. 200.1068 (δ 0.8 ppm).

### 4-(3-Nitrobenzyl)­pyridine (**3c**)

4-Pyridinyl boronic acid (74 mg, 0.60 mmol), 3-nitrobenzyl chloride (86 mg, 0.50 mmol), and Na_2_CO_3_ (111 mg, 0.126 mmol) were combined in a microwave vessel and flushed with *N*_2_(*g*) for 5 min. Pd­(PPh_3_)_4_ (12 mg, 0.01 mmol) was added, and the reaction vessel was flushed with *N*_2_(*g*) for a further 2–3 min before the addition of a mixture of DME and water (2:1 v/v, 3 mL). The reaction was heated at 100 °C for 4 h, then allowed to cool to room temperature and diluted with water (6 mL) and DCM (6 mL). The phases were separated, and the aqueous fraction was extracted with DCM (3 × 3 mL). The organic fractions were combined, dried over anhydrous Na_2_SO_4_ and the solvent was removed under reduced pressure. The crude material was purified twice by flash chromatography (0–5% v/v MeOH in DCM) to yield compound **3c** as an orange crystalline solid (93.8 mg, 0.44 mmol, 88%), which retained a small of PPh_3_ impurity. *R*_f_ 0.47 (10% v/v MeOH in DCM); ^1^H NMR (500 MHz, CDCl_3_) δ 8.55 (d, *J* = 6.1 Hz, 2H), 8.12 (m, 1H), 8.07 (m, 1H), 7.51 (d, *J* = 1.1 Hz, 1H), 7.50 (dd, *J* = 2.1, 1.1 Hz, 1H), 7.11 (d, *J* = 6.1 Hz, 1H), 4.08 (s, 2H) ppm; ^13^C NMR (125 MHz, CDCl_3_) δ 150.2, 148.5, 148.1, 140.9, 135.1, 129.7, 124.0, 123.9, 122.0, 40.8 ppm; IR (solid) *v*_max_ 3099, 3066, 3024, 1669, 1595, 1561, 1510, 1477, 1439, 1416, 1358, 1346, 1315, 1217, 1098, 1076, 994, 912, 900, 843, 806, 819, 792, 731, 685 (s, C–H), 673, 614 cm^–1^; LCMS (+ESI) *m*/*z* 215.2 [M + H]^+^, 1.26 min, (100%); HRMS (+ESI) *m*/*z* (Calcd C_14_H_22_N_3_O [M + H]^+^, 248.1757), *Obs.* 248.1748 (δ 3.8 ppm).

### 3-(Pyridin-4-ylmethyl)­aniline (**3d**)

Pd/C (17 mg, 0.16 mmol) was added as a single portion to a warm (55 °C) suspension of the **3c** (172 mg, 0.80 mmol) and hydrazine hydrate (125 μL, 4.02 mmol) in absolute EtOH (12 mL). The reaction was heated at reflux for 2 h, at which point not starting material remained by LCMS analysis and the solution was colorless. The reaction was cooled to room temperature, filtered through filter paper, then through a short (1–2 cm) plug of silica, using a solution of 10% v/v MeOH in DCM (10–20 mL) to elute the product. The solvent was removed under reduced pressure to yield compound **3d** as a white crystalline solid (105.1 mg, 0.57 mmol, 71%). *R*_f_ 0.17 (5% v/v MeOH in DCM); ^1^H NMR (500 MHz, CDCl_3_) δ 8.48 (d, *J* = 5.9 Hz, 2H), 7.13 (d, *J* = 5.4 Hz, 2H), 7.10 (app. t, *J* = 7.8 Hz, 1H), 6.57 (d, *J* = 7.7 Hz, 2H), 6.47 (app. t, *J* = 2.0 Hz, 1H), 3.87 (s, 2H) ppm; ^13^C NMR (125 MHz, CDCl_3_) δ 150.8, 149.5, 149.4, 146.9, 140.0, 129.8, 124.5, 119.4, 115.7, 113.6, 41.4 ppm; IR (solid) *v*_max_ 3425, 3317, 3194, 3029, 2093, 1629, 1596, 1585, 1557, 1493, 1459, 1416, 1315, 1266, 1175, 1158, 1119, 997, 858, 809, 773, 752, 721, 693 cm^–1^; LCMS (+ESI) *m*/*z* 185.2 [M + H]^+^, 0.34 min, (100%); HRMS (+ESI) *m*/*z* (Calcd C_12_H_13_N_2_ [M + H]^+^, 185.1073), Obs. 185.1071 (δ 1.2 ppm).

### *N*-(4-(Pyridin-4-ylmethyl)­phenyl)­acetamide (**3f**)

Acetic anhydride (115 μL, 1.1 mmol, 1.1 equiv) was added to a stirred solution of 4-(4-aminophenyl)­pyridine (184 mg, 1 mmol), and triethylamine (279 μL, 2 mmol) in DCM (5 mL), and the reaction was stirred overnight. Water (10 mL) was added, and the product was extracted with DCM (3 × 10 mL), dried over anhydrous Na_2_SO_4_ and the solvent removed under reduced pressure. The crude material was purified by flash chromatography (0–5% MeOH in DCM) to yield **3f** as a white solid (193 mg, 0.85 mmol, 85%).

^1^H NMR (400 MHz, MeOD) δ 8.39 (m, 2H), 7.49 (m, 2H), 7.25 (dd, *J* = 8.6, 6.3 Hz, 2H), 7.16 (d, *J* = 8.7 Hz, 2H), 3.97 (s, 2H), 2.10 (s, 3H) ppm; ^13^C NMR (100 MHz, MeOD) δ 171.6, 153.5, 149.9, 138.5, 136.1, 130.4, 125.8, 121.5, 41.3, 23.8 ppm; LCMS (+ESI) *m*/*z* 227.1 [M + H]^+^, 0.70 min; (100%); HRMS (+ESI) *m*/*z* (Calcd C_14_H_15_N_2_O [M + H]^+^, 227.1179), Obs. 227.1178 (δ 0.6 ppm).

### *N*-(4-Pyridin-4-ylmethyl)­phenyl)­methanesulfonamide (**3g**)

Methane sulfonyl chloride (85 μL, 1.1 mmol, 1.1 equiv) was added to a stirred solution of 4-(4-aminophenyl)­pyridine (184 mg, 1 mmol), and triethylamine (279 μL, 2 mmol) in DCM (5 mL), and the reaction was stirred overnight. Water (10 mL) was added, and the product was extracted with DCM (3 × 10 mL), dried over anhydrous Na_2_SO_4_ and the solvent removed under reduced pressure. The crude material was purified by flash chromatography (0–5% MeOH in DCM) to yield **3g** as an off-white solid (167 mg, 0.64 mmol, 64%). ^1^H NMR (400 MHz, *d*_6_-DMSO) δ 9.67 (s, 1H), 8.45 (m, 2H), 7.23 (m, 2H), 7.21 (d, *J* = 8.9 Hz, 2H), 7.16 (d, *J* = 8.7 Hz, 2H), 3.91 (s, 2H), 2.95 (s, 3H) ppm; ^13^C NMR (100 MHz, *d*_6_-DMSO) δ 150.1, 149.7, 136.7, 135.2, 129.8, 129.4, 124.1, 120.3, 39.5, 39.2 ppm; LCMS (+ESI) *m*/*z* 263.1 [M + H]^+^, 0.70 min, (98%); HRMS (+ESI) *m*/*z* (Calcd C_13_H_15_N_2_O_2_S [M + H]^+^, 263.0849), Obs. 263.0847 (δ 0.8 ppm).

### *N*-(3-Nitrophenyl)­pyridin-4-amine (**6a**)

3-Nitroaniline (414 mg, 3.00 mmol) was added to a solution of 4-chloropyridine HCl (450 mg, 3.00 mmol) in EtOH (15 mL), followed by a catalytic amount of conc. HCl (37%, 4 drops). The reaction was heated at 90 °C for 24 h, at which point a yellow precipitate had formed. The reaction was allowed to cool to room temperature, and then the precipitate was collected by vacuum filtration, washed with ice cold EtOH, and dried under reduced pressure to yield compound **6a** as a yellow solid (411 mg, 1.91 mmol, 64%), which was used without further purification. *R*_f_ 0.02 (5% v/v MeOH in DCM); ^1^H NMR (500 MHz, *d*_6_-DMSO) δ 14.22 (s, 1H), 11.32 (s, 1H), 8.37 (d, *J* = 7.4 Hz, 2H), 8.16 (app. t, *J* = 2.1 Hz, 1H), 8.11 (ddd, *J* = 8.2, 2.2, 1.0 Hz, 1H), 7.84 (ddd, *J* = 8.0, 2.1, 0.9 Hz, 1H), 7.76 (app. t, *J* = 8.1 Hz, 1H), 7.32 (d, *J* = 7.5 Hz, 1H) ppm; ^13^C NMR (125 MHz, *d*_6_-DMSO) δ 155.9, 148.6, 140.9, 138.8, 131.3, 129.0, 120.3, 117.2, 109.4 ppm; IR (solid) *v*_max_ 3178–2700, 3066, 3036, 2872, 2831, 1646, 1610, 1584, 1563, 1523, 1479, 1355, 1342, 1317, 1304, 1243, 1233, 1202, 1103, 1003, 946, 898, 856, 840, 800, 791, 739, 700, 677 cm^–1^; LCMS (+ESI) *m*/*z* 216.1 [M + H]^+^, retention time 1.29 min, (97%); HRMS (+ESI) *m*/*z* (Calcd C_11_H_10_N_3_O_2_ [M + H]^+^, 216.0768), *Obs*. 216.0762 (δ 2.8 ppm).

### *N*-(4-Nitrophenyl)­pyridin-4-amine (**6b**)

4-Nitroaniline (414 mg, 3.00 mmol) was added to a solution of 4-chloropyridine HCl (450 mg, 3.00 mmol) in EtOH (15 mL), followed by a catalytic amount of conc. HCl (37%, 4 drops). The reaction was heated at 90 °C for 24 h, at which point a yellow precipitate had formed. The reaction was allowed to cool to room temperature, and then the precipitate was collected by vacuum filtration, washed with ice cold EtOH, and dried under reduced pressure to yield compound **6b** as a yellow solid ((352 mg, 1.64 mmol, 55%), which was used without further purification. *R*_f_ 0.18 (10% v/v MeOH in EtOAc); ^1^H NMR (500 MHz, *d*_6_-DMSO) δ 14.40 (br s, 2H), 11.51 (s, 1H), 8.45 (d, *J* = 7.4 Hz, 2H), 8.31 (d, *J* = 9.0 Hz, 2H), 7.62 (d, *J* = 9.1 Hz, 2H), 7.47 (d, *J* = 7.4 Hz, 2H) ppm; ^13^C NMR (125 MHz, *d*_6_-DMSO) δ 155.0, 144.2, 143.6, 141.2, 125.5, 121.6, 110.6 ppm; IR (solid) *v*_max_ 3076–2600, 3029, 2941, 1640, 1621, 1589, 1581, 1501, 1486, 1334, 1208, 1293, 1236, 1207, 1172, 1111, 1008, 853, 836, 810, 750, 736, 698, 631 cm^1^; LCMS (+ESI) *m*/*z* 216.1 [M + H]^+^, retention time 1.31 min, (100%); HRMS (+ESI) *m*/*z* (Calcd C_11_H_10_N_3_O_2_ [M + H]^+^, 216.0768), *Obs.* 216.0764 (δ 1.8 ppm).

### N^1^-(Pyridin-4-yl)­benzene-1,3-diamine (**4a**)

Tin­(II) chloride dihydrate (1.08 g, 4.80 mmol) was added to a stirred solution of N-(3-nitrophenyl)­pyridin-4-amine (**6a**) (207 mg, 0.96 mmol) in EtOH (6 mL). The reaction was cooled to 0 °C and concentrated HCl (37% v/v soln., 100 μL,) was added dropwise. The reaction was then heated under reflux for 3 h, then allowed to cool to room temperature and quenched with 2 M Na_2_CO_3_ (∼5 mL). The product was extracted into EtOAc (4 × 10 mL) the organic fractions were combined, washed with brine (5 mL), dried over anhydrous Na_2_SO_4_, and then the solvent was removed under reduced pressure to yield compound **4a** as a yellow solid (152 mg, 0.82 mmol, 86%). ^1^H NMR (700 MHz, *d*_6_-DMSO) δ 8.51 (s, 1H), 8.13 (d, *J* = 6.5 Hz, 1H), 6.95 (app. t, *J* = 7.9 Hz, 1H), 6.85 (m, 2H), 6.43 (app. t, *J* = 2.1 Hz, 1H), 6.32 (ddd, *J* = 7.8, 2.0, 0.7 Hz, 1H), 6.25 (ddd, *J* = 8.0, 2.1, 0.9 Hz, 1H). ppm; ^13^C NMR (175 MHz, *d*_6_-DMSO) δ 150.4, 149.9, 149.7, 141.0, 129.6, 109.2, 108.9, 108.0, 105.5 ppm; IR (solid) *v*_max_ 3455, 3372, 3199–2700, 3055, 2920, 2892, 2843, 2811, 1606, 1579, 1524, 1484, 1445, 1347, 1300, 1242, 1215, 1182, 1160, 1098, 1054, 992, 965, 841, 826, 779, 695, 657 cm^–1^; LCMS (+ESI) *m*/*z* 186.2 [M + H]^+^, retention time 0.46 min, (100%); HRMS (+ESI) *m*/*z* (Calcd C_11_H_12_N_3_ [M + H]^+^, 186.1026), *Obs*. 186.1022 (δ 1.9 ppm).

### N^1^-(Pyridin-4-yl)­benzene-1,4-diamine (**4b**)

Tin­(II) chloride dihydrate (0.56 g, 2.5 mmol) was added to a stirred solution of N-(4-nitrophenyl)­pyridin-4-amine (**6b**) (108 mg, 0.50 mmol) in EtOH (3 mL). The reaction was cooled to 0 °C and concentrated HCl (37% v/v soln., 100 μL,) was added dropwise. The reaction was then heated under reflux for 1 h, at which point all starting material had been consumed and an orange precipitate formed. The reaction was cooled to room temperature, quenched with 2 M Na_2_CO_3_ (∼5 mL), and the product was extracted into EtOAc (4 × 5 mL). The organic fractions were combined, washed with brine (2 mL), dried over anhydrous Na_2_SO_4_, and the solvent was removed under reduced pressure to yield compound **4b** as a yellow solid (88 mg, 0.48 mmol, 95%). ^1^H NMR (500 MHz, *d*_6_-DMSO) δ 8.21 (s, 1H), 8.03 (d, *J* = 6.5 Hz, 2H), 6.86 (d, *J* = 8.5 Hz, 2H), 6.63–6.53 (m, 4H), 4.97 (br s, 2H) ppm; ^13^C NMR (125 MHz, *d*_6_-DMSO) δ 152.3, 149.7, 145.6, 128.3, 124.4, 114.5, 107.8 ppm; IR (solid) *v*_max_ 3429, 3379, 3301, 3140, 3029, 1643, 1594, 1570, 1506, 1435, 1411, 1345, 1325, 1282, 1216, 1173, 991, 885, 807, 648, 613 cm^–1^; LCMS (+ESI) *m*/*z* 186.2 [M + H]^+^, retention time 0.31 min, (100%); HRMS (+ESI) *m*/*z* (Calcd C_11_H_12_N_3_ [M + H]^+^, 186.1026), *Obs*. 186.1018 (δ 4.8 ppm).

### Methyl/Ethyl 3-(pyridin-4-ylamino)­benzoate (**4c**)

Methyl 3-aminobenzoate (453 mg, 3.00 mmol) was added to a solution of 4-chloropyridine hydrochloride (450 mg, 3.00 mmol) in EtOH (15 mL). A catalytic amount of conc. HCl (37%, 4 drops) was added and the reaction was heated at 90 °C for 24 h and then the solvent was removed under reduced pressure. The resulting residue was redissolved in DCM (60 mL), washed with saturated NaHCO_3_ (15 mL), and brine (10 mL), dried over anhydrous Na_2_SO_4_ and the solvent was removed under reduced pressure. The crude material was purified by flash chromatography (0–10% v/v MeOH in DCM) to yield **4c**, which was a mixture of the methyl and ethyl 3-(pyridin-4-ylamino)­benzoates in 1.7:1.3 ratio by ^1^H NMR integration, respectively, as a pink amorphous solid (298 mg, 1.3 mmol, 24/18%). *R*_f_ 0.14 (10% v/v MeOH in DCM); ^1^H NMR (500 MHz, CDCl_3_) δ 8.32 (d, *J* = 5.0 Hz, 2H), 7.86 (d, *J* = 1.4 Hz, 1H), 7.78 (m, 1H), 7.43 (app. t, *J* = 7.7 Hz, 1H), 7.39 (d, *J* = 7.7 Hz, 1H), 6.84 (d, *J* = 4.9 Hz, 2H), 6.39 (d, *J* = 5.3 Hz, 1H), 4.38 (q, *J* = 7.1 Hz, 1H), 3.92 (s, 2H), 1.39 (t, *J* = 7.1 Hz, 1H) ppm; ^13^C NMR (125 MHz, CDCl_3_) δ 166.7, 166.2, 150.7, 150.2, 150.1, 140.2, 140.1, 132.2, 131.8, 129.8, 129.7, 125.6, 125.5, 125.1, 122.3, 122.2, 109.9, 109.8, 61.4, 52.5, 14.5 ppm; IR (solid) *v*_max_ 3164–2800, 3058, 2916, 2819, 1717, 1625, 1587, 1569, 1527, 1500, 1479, 1435, 1418, 1345, 1294, 1266, 1214, 1160, 1102, 1077, 994, 813, 749, 690, 670 cm^–1^; LCMS (+ESI) *m*/*z* 229.0, 243.0 [M + H]^+^, retention time 1.38, 1.53 min, (55%, 45%).

### Methyl 4-(pyridin-4-ylamino)­benzoate (**4d**)

Methyl 4-aminobenzoate (252 mg, 1.67 mmol) was added to a stirred solution of 4-chloropyridine hydrochloride (250 mg, 1.67 mmol) in glacial AcOH (2.5 mL). The reaction was heated at 100 °C for 20 h and then concentrated under reduced pressure. The resulting residue was resuspended in water (2.5 mL) and brought to pH 10 using 6 M NaOH. The product was extracted into CHCl_3_ (4 × 4 mL), concentrated and then purified by flash chromatography (0–5% v/v MeOH in DCM) to yield compound **4d** as a white solid (164 mg, 0.72 mmol, 43%). ^1^H NMR (400 MHz, MeOD) δ 8.22 (dd, *J* = 7.2, 1.3 Hz, 2H), 7.99 (dd, *J* = 6.8, 2.0 Hz, 2H), 7.29 (dd, *J* = 6.8, 2.0 Hz, 2H), 7.10 (dd, *J* = 5.0, 1.6 Hz, 2H), 3.88 (s, 3H) ppm; ^13^C NMR (100 MHz, MeOD) δ 168.4, 152.2, 150.2, 146.6, 132.4, 125.2, 119.8, 112.0, 52.6 ppm; IR (solid) *v*_max_ 2920, 1709, 1638, 1583, 1522, 1502, 1431, 1345, 1267, 1104, 998, 818 cm^–1^; LCMS (+ESI) *m*/*z* 229.0 [M + H]^+^, retention time 1.26 min (95%); HRMS (+ESI) *m*/*z* (Calcd C_13_H_13_N_2_O_2_ [M + H]^+^, 229.0972), *Obs*. 229.0961 (δ 4.8 ppm).

### 3-(Pyridin-4-ylamino)­benzoic Acid (**4e**)

Lithium hydroxide monohydrate (319 mg, 7.6 mmol) was added to a solution of ester **4c** (288 mg, approximately 55:45 methyl/ethyl ester, ∼1.27 mmol) in THF:MeOH:H_2_O (2:1:1) (6 mL) and the reaction was stirred at room temperature for 4 h. The reaction was then concentrated under reduced pressure and the residue was redissolved in water (75 mL) and brought to pH ∼ 1–2 using 3 M HCl. The product was extracted into a mixture of CHCl_3_ and *i*-PrOH (2:1, 20 × 30 mL). The organic fractions were combined and concentrated under reduced pressure to yield **4e** as a pink amorphous solid (133 mg, 0.62 mmol, 49%). ^1^H NMR (400 MHz, MeOD) δ 8.21 (d, *J* = 7.5 Hz, 2H), 8.01–7.97 (m, 2H), 7.65–7.58 (m, 2H), 7.16 (d, *J* = 7.5 Hz, 2H) ppm; ^13^C NMR (100 MHz, MeOD) δ 168.6, 158.7, 141.7, 138.7, 134.1, 131.4, 129.2, 129.2, 125.8, 125.6 ppm; IR (solid) *v*_max_ 3500–2800, 3404, 3228, 3086, 2981, 1720, 1644, 1586, 1571, 1524, 1493, 1476, 1430, 1384, 1306, 1270, 1255, 1212, 1201, 1110, 1003, 934, 900, 822, 791, 747, 688, 663 cm^–1^; LCMS (+ESI) *m*/*z* 215.0, 215.0 [M + H]+, retention time 0.31, 1.19 min, (17%, 83%); HRMS (-ESI) *m*/*z* (Calcd C_12_H_9_N_2_O_2_ [M-H]^−^, 213.0664), *Obs*. 213.0674 (δ 2.3 ppm).

### 4-(Pyridin-4-ylamino)­benzoic Acid (**4f**)

A solution of potassium hydroxide (15 mg, 0.26 mmol) in water (1.4 mL) was added to a stirred solution of ester **4d** (30 mg, 0.13 mmol) in EtOH (0.7 mL) and the reaction was heated under reflux for 2 h. The reaction was then concentrated under reduced pressure brought to pH 2 using 2 M HCl. The mixture was washed with CHCl_3_ (8 × 25 mL) and then the aqueous fraction was concentrated under reduced pressure. The residue was redissolved in water (15 mL), brought to pH 4 and the product was extracted using EtOAc (15 × 20 mL) and then CHCl_3_/*i-*PrOH (2:1 v/v, 5 × 15 mL). The organic fractions were combined and the solvent was removed under reduced pressure to yield compound **4f** as a white solid (11 mg, 0.05 mmol, 39%). ^1^H NMR (400 MHz, MeOD) δ 8.22 (d, *J* = 6.3 Hz, 2H), 8.09 (d, *J* = 8.6 Hz, 2H), 7.39 (*J* = 8.6 Hz, 2H), 7.21 (d, *J* = 6.3 Hz, 2H) ppm; ^13^C NMR (100 MHz, MeOD) δ 171.2, 156.9, 142.0, 141.2, 131.1, 121.4, 109.5, 93.9 ppm; IR (solid) *v*_max_ 2920, 2820, 1708, 1647, 1603, 1531, 1493, 1476, 1430, 1384, 1306, 1270, 1255, 1212 cm^–1^; LCMS (+ESI) *m*/*z* 215.2 [M + H]^+^; HRMS (-ESI) *m*/*z* (Calcd C_12_H_9_N_2_O_2_ [M + H]^+^, 215.0815), *Obs*. 215.0818 (δ 1.4 ppm).

### (3-Pyridin-4-ylamino)­phenyl)­methanol (**4g**)

LiAlH_4_ (10 mg, 0.26 mmol) was added slowly, under an inert atmosphere to a stirred solution of ester **4c** (30 mg, 0.13 mmol) in anhydrous THF (0.7 mL) at 0 °C. When the addition was complete, the reaction was allowed to warm slowly to room temperature and stirred for 16 h. As starting material remained, additional LiAlH_4_ (25 mg, 0.66 mmol) in THF (1.5 mL) was added and the reaction was stirred for a further 4 h at room temperature before being quenched with water (0.5 mL), followed by 4 M NaOH (0.5 mL), and additional water (1.5 mL). The reaction was then filtered through Celite, eluting the product with DCM (1 mL). The organic solvent was collected and concentrated under reduced pressure to yield compound **4g** as a yellow solid (24 mg, 0.12 mmol, 92%). ^1^H NMR (400 MHz, MeOD) δ 8.10 (d, *J* = 5.4 Hz, 2H), 7.34 (app.t, *J* = 7.8 Hz, 2H), 7.25 (s, 1H), 7.09 (d, *J* = 7.8 Hz, 1H), 7.12 (d, *J* = 7.8 Hz, 1H), 6.93 (d, *J* = 5.4 Hz, 2H), 4.61 (s, 2H) ppm; ^13^C NMR (100 MHz, MeOD) δ 153.7, 150.0, 144.5, 141.6, 130.6, 123.5, 121.4, 121.0, 110.3, 65.1 ppm; IR (solid) *v*_max_ 3264, 3059, 1615, 1591, 1520, 1474, 1345, 1030, 1000, 817 cm^–1^; LCMS (+ESI) *m*/*z* 201.2 [M + H]^+^; HRMS (+ESI) *m*/*z* (Calcd C_12_H_13_N_2_O [M + H]^+^, 201.1028), *Obs.* 201.1025 (δ 1.5 ppm).

### *N*-(3-Bromophenyl)­pyridin-4-amine (**4h**)

4-Aminopyridine (500 mg, 5.31 mmol), 1-bromo-3-iodobenzene (0.75 mg, 5.8 mmol), NaO^*t*^Bu (608 mg, 16.32 mmol), Pd_2_(dba)_3_ (73 mg, 0.19 mmol), and DPPF (107 mg, 0.19 mmol) were combined in anhydrous toluene (16 mL) and heated under reflux (115 °C) for 24 h. The reaction was then allowed to cool to room temperature, diluted with Et_2_O (50 mL) and filtered through a short plug of Celite. The filtrate was concentrated under reduced pressure and the crude material was purified by flash chromatography (0–5% v/v MeOH in DCM), to yield compound **4h** as a brown solid (755 mg, 3.0 mmol, 57%). *R*_f_ 0.07 (10% v/v MeOH in DCM); ^1^H NMR (400 MHz, MeOD) δ 8.16 (d, *J* = 5.1 Hz, 2H), 7.37 (dd, *J* = 1.9, 1.9 Hz, 1H), 7.29–7.19 (m, 3H), 6.95 (d, *J* = 5.1 Hz, 2H), 6.07 (br, s, 1H) ppm; ^13^C NMR (100 MHz, MeOD) δ 152.8, 150.5, 143.4, 132.1, 127.4, 124.0, 120.5 (2C), 111.0 ppm; IR (solid) *v*_max_ 2913, 1606, 1580, 1520, 1469, 1346, 1215, 993, 808 cm^–1^; HRMS (+ESI) *m*/*z* (Calcd C_11_H_10_BrN_2_ [M + H]^+^, 249.0022), *Obs.* 249.0017 (δ 2.0 ppm).

### *N*-(4-Bromophenyl)­pyridin-4-amine (**4i**)

4-Chloropyrine hydrochloride (500 mg, 3.33 mmol) was desalted using 6 M NaOH and extracted into Et_2_O (4 × 15 mL). The solution was dried over anhydrous Na_2_SO_4_ and then the solvent was removed under reduced pressure to yield a solid product. Desalted 4-chloropyridine (113 mg, 1 mmol), KO^*t*^Bu (168 mg, 1.5 mmol), Pd_2_(dba)_3_ (18 mg, 0.02 mmol), 1,3-bis­(2,6-diisopropylphenyl)­imidazol-2-ylidene hydrochloride (9 mg, 0.02 mmol), and 4-bromoaniline (189 mg, 1.1 mmol) were combined in 1,4-dioxane (3.1 mL) heated under an inert atmosphere. The reaction was heated at 100 °C for 21 h, then cooled to room temperature and diluted with 1 M HCl (7 mL). The solution was washed with E_2_O (2 × 4 mL) and then the aqueous phase was brought to pH 14 using 6 M NaOH. The product was extracted into CHCl_3_ (3 × 4 mL), the organic fractions were combined, washed with brine (4 mL), dried over Na_2_SO_4_ and the solvent was removed under reduced pressure. The crude product was purified by flash chromatography (2:3 v/v DCM:EtOAC) to yield compound **4i** as a brown solid (25 mg, 0.10 mmol, 10%). ^1^H NMR (500 MHz, CDCl_3_) δ 8.29 (dd, *J* = 4.8, 1.6 Hz, 2H), 7.45 (dd, *J* = 6.6, 2.2 Hz, 2H), 7.05 (dd, *J* = 6.6, 2.2 Hz, 2H), 6.77 (dd, *J* = 4.8, 1.6 Hz, 2H), 5.93 (s, 1H) ppm; ^13^C NMR (125 MHz, CDCl_3_) δ 150.9, 150.1, 138.9, 132.8, 123.3, 116.9, 109.9 ppm; IR (solid) *v*_max_ 2902, 1739, 1636, 1603, 1576, 1524, 1484, 1430, 1346, 1217, 1071, 996 cm^–1^; LCMS (+ESI) *m*/*z* 248.9 [M + H]^+^, retention time 1.43 min (100%); HRMS (+ESI) *m*/*z* (C

### *N*-((1*H*-Indol-5-yl)­methyl)-3-(chloromethyl)­benzamide (**7a**)

HBTU (455 mg, 1.20 mmol) was added to a solution of 3-(chloromethyl)­benzoic acid (225 mg, 1.32 mmol) in anhydrous DCM (1 mL) and anhydrous DMF (250 μL) and stirred for 10 min at room temperature. Then, a solution of 5-(aminomethyl)­indole (153 mg, 1.2 mmol) and Et_3_N (250 μL, 1.8 mmol) in anhydrous DCM (1 mL) was added slowly, and the reaction was stirred at room temperature for 24 h. The reaction was then diluted with EtOAc (50 mL), washed with saturated citric acid (6 mL), and saturated NaHCO_3_ (10 mL) until basic, followed by water (3 × 3 mL), brine (3 mL), dried over anhydrous Na_2_SO_4_, and the solvent was removed under reduced pressure. The crude product was purified twice by flash chromatography (0–50% v/v EtOAc in PE), to yield compound **7a** as a white solid (202 mg, 0.68 mmol, 56%). *R*_f_ 0.37 (50% v/v EtOAc in PE); ^1^H NMR (500 MHz, CDCl_3_) δ 8.27 (br s, 1H), 7.81 (td, *J* = 1.9, 0.5 Hz, 1H), 7.72 (ddd, *J* = 7.7, 1.8, 1.2 Hz, 1H), 7.64 (dt, *J* = 1.6, 0.8 Hz, 1H), 7.52 (dtd, *J* = 7.7, 1.3, 0.6 Hz, 1H), 7.43 (dd, *J* = 7.7, 0.5 Hz, 1H), 7.40 (dt, *J* = 8.3, 0.8 Hz, 1H), 7.24 (dd, *J* = 3.2, 2.4 Hz, 1H), 7.21 (dd, *J* = 8.4, 1.7 Hz, 1H), 6.55 (ddd, *J* = 3.1, 2.0, 0.9 Hz, 1H), 6.37 (br s, 1H), 4.74 (d, *J* = 5.3 Hz, 2H), 4.60 (s, 2H) ppm; ^13^C NMR (125 MHz, CDCl_3_) δ 166.7, 138.2, 135.5, 135.3, 131.6, 129.4, 129.2, 128.3, 127.4, 127.0, 125.1, 122.6, 120.6, 111.6, 102.8, 45.7, 45.1 ppm; IR (solid) *v*_max_ 3271, 2922, 2547, 1633, 1605, 1581, 1532, 1482, 1427, 1355, 1298, 1263, 1216, 1093, 1052, 1000, 876, 805, 753, 704 cm^–1^; LCMS (+ESI) *m*/*z* 299.2 [M + H]^+^, retention time 1.96 min, (100%); HRMS (+ESI) *m*/*z* (Calcd C_17_H_15_N_2_OClNa [M + Na]^+^, 321.0765), *Obs*. 321.0763 (δ 0.6 ppm).

### *N*-((^1^H-Indol-5-yl)­methyl)-4-(chloromethyl)­benzamide (**7b**))

HBTU (228 mg, 0.60 mmol) was added to a stirred solution of 4-(chloromethyl)­benzoic acid (113 mg, 0.66 mmol) in anhydrous DMF (700 μL). After 10 min, a solution of 5-(aminomethyl)­indole (88 mg, 0.60 mmol) and Et_3_N (125 μL, 0.9 mmol) in anhydrous DMF (300 μL) was added to the stirred reaction over period of 1–2 min. The reaction was stirred at room temperature for 24 h then diluted with EtOAc (25 mL), washed with saturated citric acid (3 mL), saturated NaHCO_3_ (10 mL), dried over anhydrous Na_2_SO_4_ and the solvent was removed under reduced pressure. The crude product was purified twice by flash chromatography (0–50% v/v EtOAc in PE), then redissolved in EtOAc (50 mL) and washed with water (4 × 5 mL), and brine (5 mL) to remove residual DMF. The solvent was removed under reduced pressure to yield compound **7b** as an off-white solid (81 mg, 0.27 mmol, 45%). *R*_f_ 0.29 (1:2 v/v EtOAc:PE); ^1^H NMR (500 MHz, CDCl_3_) δ 8.25 (br s, 1H), 7.78 (d, *J* = 8.3 Hz, 2H), 7.63 (s, 1H), 7.44 (d, *J* = 8.3 Hz, 2H), 7.39 (d, *J* = 8.3 Hz, 1H), 7.24 (app. t, *J* = 2.8 Hz, 1H), 7.20 (dd, *J* = 8.3, 1.6 Hz, 1H), 6.54 (ddd, *J* = 3.1, 2.0, 1.0 Hz, 1H), 6.35 (br s, 1H), 4.73 (d, *J* = 5.4 Hz, 2H), 4.60 (s, 2H) ppm; ^13^C NMR (125 MHz, CDCl_3_) δ 166.7, 140.9, 135.5, 134.7, 129.4, 128.8, 128.3, 127.5, 125.1, 122.6, 120.5, 111.6, 102.8, 45.5, 45.0 ppm; IR (solid) *v*_max_ 3332, 2925, 1620, 1570, 1543, 1505, 1465, 1423, 1359, 1329, 1303, 1269, 1185, 1095, 1033 987, 867, 852, 752, 723, 677 cm^–1^; LCMS (+ESI) *m*/*z* 299.2 [M + H]^+^, 1.92 min, (98%); HRMS (+ESI) *m*/*z* (Calcd C_17_H_16_N_2_OCl [M + H]^+^, 299.0946), *Obs*. 299.0941 (δ 1.4 ppm).

### (*R*)-3-(Chloromethyl)-*N*-(1-phenylethyl)­benzamide (**7c**)

HBTU (455 mg, 1.20 mmol) was added to a solution of 3-(chloromethyl)­benzoic acid (225 mg, 1.32 mmol) in anhydrous DCM (1 mL) and anhydrous DMF (250 μL) and stirred for 10 min at room temperature. Then, a solution of (*R*)*-*(+)-alpha-methylbenzylamine (153 μL, 1.2 mmol) and Et_3_N (250 μL, 1.8 mmol) in anhydrous DCM (1 mL) was added slowly, and the reaction was stirred at room temperature for 24 h. The reaction was then diluted with EtOAc (50 mL), washed with saturated citric acid (6 mL), and saturated NaHCO_3_ (10 mL) until basic, followed by water (3 × 3 mL), brine (3 mL), dried over anhydrous Na_2_SO_4_, and the solvent was removed under reduced pressure. The crude product was purified by flash chromatography (0–40% v/v EtOAc in PE), to yield compound **7c** as a white solid (194 mg, 0.71 mmol, 59%). *R*_f_ 0.45 (50% v/v EtOAc in PE); ^1^H NMR (500 MHz, CDCl_3_) δ 7.79 (app. t, *J* = 1.8 Hz, 1H), 7.71 (dt, *J* = 7.8, 1.5 Hz, 1H), 7.53 (dt, *J* = 7.7, 1.5 Hz, 1H), 7.43 (d, *J* = 7.7 Hz, 1H), 7.41–7.39 (m, 2H), 7.38–7.35 (m, 2H), 7.29 (tt, *J* = 7.2, 1.7 Hz, 1H), 6.34 (d, *J* = 7.7 Hz, 1H), 5.34 (m, 1H), 1.62 (d, *J* = 6.9 Hz, 3H) ppm; ^13^C NMR (125 MHz, CDCl_3_) δ 166.1, 143.1, 138.2, 135.3, 131.7, 129.2, 128.9, 127.7, 127.3, 126.9, 126.4, 49.5, 45.7, 21.8 ppm; IR (solid) *v*_max_ 3309, 3060, 2978, 2931, 2874, 1635, 1603, 1589, 1536, 1494, 1446, 1322, 1281, 1260, 1221, 1140, 1094, 1082, 1015, 897, 824, 761, 701, 666 cm^–1^; LCMS (+ESI) *m*/*z* 296.0 [M + Na]^+^, 2.08 min, (100%); HRMS (+ESI) *m*/*z* (Calcd C_16_H_17_NOCl [M + H]^+^, 274.0993), *Obs.* 274.0982 (δ 4.2 ppm).

### (*R*)-4-(Chloromethyl)-*N*-(1-phenylethyl)­benzamide (**7d**)

HBTU (455 mg, 1.20 mmol) was added to a solution of 4-(chloromethyl)­benzoic acid (225 mg, 1.32 mmol) in anhydrous DCM (1 mL) and anhydrous DMF (250 μL) and stirred for 10 min at room temperature. Then, a solution of (*R*)-(+)-alpha-methylbenzylamine (153 μL, 1.2 mmol) and Et_3_N (250 μL, 1.8 mmol) in anhydrous DCM (1 mL) was added slowly, and the reaction was stirred at room temperature for 24 h. The reaction was then diluted with EtOAc (50 mL), washed with saturated citric acid (6 mL), and saturated NaHCO_3_ (10 mL) until basic, followed by water (3 × 3 mL), brine (3 mL), dried over anhydrous Na_2_SO_4_, and the solvent was removed under reduced pressure. The crude product was purified by flash chromatography (0–40% v/v EtOAc in PE), to yield compound **7d** as a white solid (195 mg, 0.71 mmol, 59%). *R*_f_ 0.55 (50% v/v EtOAc in PE); ^1^H NMR (500 MHz, CDCl_3_) δ 7.76 (d, *J* = 8.3 Hz, 2H), 7.44 (d, *J* = 8.4 Hz, 2H), 7.38–7.35 (m, 4H), 7.29 (tt, *J* = 7.0, 6.7, 1.6 Hz, 1H), 6.31 (d, *J* = 7.8 Hz, 1H), 5.34 (m, 1H), 4.60 (s, 2H), 1.61 (d, *J* = 6.9 Hz, 3H) ppm; ^13^C NMR (125 MHz, CDCl_3_) δ 166.1, 143.1, 141.0, 134.7, 128.9, 128.8, 127.7, 127.5, 126.4, 49.4, 45.5, 21.8 ppm; IR (solid) *v*_max_ 3334, 3035, 2980, 1627, 1571, 1528, 1502, 1495, 1450, 1322, 1279, 1210, 1149, 1123, 1087, 1013, 910, 876, 832, 815, 764, 702 cm^–1^; LCMS (+ESI) *m*/*z* 274.2 [M + H]^+^, retention time 2.10 min, (100%); HRMS (+ESI) *m*/*z* (Calcd C_16_H_16_NOClNa [M + Na]^+^, 296.0813), *Obs*. 296.0811 (δ 0.7 ppm).

### *N*-Methyl-*N*-(4-nitrophenyl)­pyridin-4-amine (**8a**)

Sodium hydride (48 mg, 1.2 mmol, 60% dispersion in mineral oil) was added as a single portion to a solution of *N*-(4-nitrophenyl)­pyridin-4-amine 6b (107 mg, 0.50 mmol) in anhydrous DMF (2.5 mL) at 0 °C. The reaction was stirred at room temperature for 45 min, then cooled to 0 °C and methyl iodide (74 μL, 1.2 mmol) was added dropwise over 2 min. The reaction was then stirred for 8 h at room temperature and then quenched with water (5 mL). The product was extracted into EtOAc (3 × 10 mL) and organic fractions were combined, washed with brine (10 mL), dried over anhydrous Na_2_SO_4_ and then the solvent was removed under reduced pressure. The crude product was purified by flash chromatography (0–10% v/v MeOH in DCM) to yield compound 8a as a brown solid (36 mg, 0.16 mmol, 32%). *R*_f_ 0.37 (10% v/v MeOH in DCM); ^1^H NMR (500 MHz, MeOD) δ 8.34–8.17 (m, 4H), 7.44 (d, *J* = 9.2 Hz, 2H), 7.01 (d, *J* = 6.6 Hz, 2H), 3.48 (s, 3H) ppm; ^13^C NMR (125 MHz, MeOD) δ 155.2, 153.2, 150.6, 145.1, 126.4, 124.8, 113.3, 39.8 ppm; IR (solid) *v*_max_ 3019, 1574, 1491, 1418, 1361, 1333, 1318, 1305, 1225, 1195, 1148, 1112, 1092, 1062, 996, 878, 851, 826, 746, 732, 695 cm^–1^; LCMS (+ESI) *m*/*z* 230.1, retention time 1.71 min, (97%); HRMS (+ESI) *m*/*z* (Calcd C_12_H_12_N_3_O_2_ [M + H]^+^, 230.0924), *Obs.* 230.0916 (δ 3.6 ppm).

### N^1^-Methyl-N^1^-(pyridin-4-yl)­benzene-1,4-diamine (**8b**)

Ammonium chloride (361 mg, 6.75 mmol) and Zn(s) (441 mg, 6.75 mmol) were added to a solution of **8a** (62 mg, 0.27 mmol) in anhydrous DMF (4 mL) and the reaction was stirred at room temperature for 24 h. The reaction was then diluted with EtOAc (25 mL) and filtered through a plug of Celite, eluting with EtOAc (100 mL). The filtrate was washed with brine (3 × 25 mL), dried over Na_2_SO_4_, and the solvent was removed under reduced pressure. The residue was redissolved in EtOAc (75 mL), washed with water (3 × 20 mL), brine (15 mL), dried over Na_2_SO_4_, and the solvent was removed under reduced pressure to yield **8b** as a brown solid (31 mg, 0.16 mmol, 58%). *R*_f_ 0.08 (20% v/v MeOH in EtOAc); ^1^H NMR (400 MHz, *d*_6_-DMSO) δ 8.04 (d, *J* = 4.5 Hz, 2H), 6.87 (d, *J* = 8.6 Hz, 2H), 6.62 (d, *J* = 8.6 Hz, 2H), 6.45 (d, *J* = 6.1 Hz, 2H), 5.20 (s, 2H), 3.16 (s, 3H) ppm; ^13^C NMR (100 MHz, *d*_6_-DMSO) δ 154.2, 149.1, 147.5, 134.0, 127.5, 114.8, 107.5, 39.4 ppm; IR (solid) *v*_max_ 3324, 3182, 1635, 1595, 1536, 1506, 1469, 1371, 1297, 1287, 1244, 1223, 1168, 1130, 1071, 987, 877, 831, 802, 727, 699, 668 cm^–1^; LCMS (+ESI) *m*/*z* 200.0, retention time 1.22 min, (97%); HRMS (+ESI) *m*/*z* (Calcd C_12_H_14_N_3_ [M + H]^+^, 200.1188), *Obs.* 200.1190 (δ 1.0 ppm).

### *N*-((1*H*-Indol-5-yl)­methyl)-3-(pyridin-4-ylmethyl)­benzamide (**5e**)

4-Pyridinylboronic acid (49 mg, 0.40 mmol), benzyl chloride **7a** (100 mg, 0.33 mmol), and Na_2_CO_3_ (74 mg, 0.70 mmol) were combined in a microwave vessel and flushed with *N*_2_(*g*) for 5 min before the addition of Pd­(PPh_3_)_4_ (8 mg, 0.01 mmol). The reaction vessel was flushed with *N*_2_(*g*) for a further 2–3 min, and then a mixture of DME and water (2:1 v/v, 2 mL) was added, and the reaction was heated at 100 °C for 4 h. The reaction was then allowed to cool to room temperature, and diluted with water (5 mL) and DCM (5 mL). The phases were separated, and the aqueous fraction was extracted with DCM (3 × 3 mL). The organic fractions were combined, dried over anhydrous Na_2_SO_4_ and the solvent was removed under reduced pressure. The crude product was purified by flash chromatography (0–5% v/v MeOH in EtOAc) to yield compound **5e** as a pale purple solid (72 mg, 0.21 mmol, 64%). *R*_f_ 0.28 (5% v/v MeOH in EtOAc); ^1^H NMR (400 MHz, MeOD) δ 8.39 (d, *J* = 6.1 Hz, 2H), 7.74 (s, 1H) 7.72 (dt, *J* = 6.2, 2.1 Hz, 1H), 7.53 (s, 1H), 7.39 (m, 2H), 7.34 (d, *J* = 8.4 Hz, 1H), 7.27 (d, *J* = 5.7 Hz, 2H), 7.20 (d, *J* = 3.1 Hz, 1H), 7.12 (dd, *J* = 8.4, 1.6 Hz, 1H), 6.40 (dd, *J* = 3.1, 0.7 Hz, 1H), 4.63 (s, 2H), 4.06 (s, 2H) ppm; ^13^C NMR (100 MHz, MeOD) δ 169.8, 152.8, 150.0, 141.0, 137.0, 136.4, 133.3, 130.4, 130.0, 129.5, 129.1, 126.7, 126.0, 125.9, 122.4, 120.4, 112.2, 102.3, 45.3, 41.7 ppm; IR (solid) *v*_max_ 3416–3250, 3031, 2920, 1639, 1600, 1581, 1526, 1480, 1416, 1322, 1285, 1216, 1099, 1067, 999, 891, 888, 809, 723, 693 cm^–1^; LCMS (+ESI) *m*/*z* 342.3 [M + H]^+^, retention time 1.40 min, (100%); HRMS (+ESI) *m*/*z* (Calcd C_22_H_20_N_3_O [M + H]^+^, 342.1606), *Obs*. 342.1602 (δ 1.2 ppm).

### *N*-((1H-Indol-5-yl)­methyl)-4-(pyridin-4-ylmethyl)­benzamide (**5f**)

4-Pyridinylboronic acid (34 mg, 0.27 mmol), benzyl chloride **7b** (68 mg, 0.23 mmol), and Na_2_CO_3_ (51 mg, 0.48 mmol) were combined in a microwave vessel and flushed with *N*_2_(*g*) for 5 min. Pd­(PPh_3_)_4_ (5 mg, 0.005 mmol) was added, and the reaction vessel was flushed with *N*_2_(*g*) for an additional 2–3 min before the addition of a mixture of DME and water (2:1 v/v, 1.5 mL). The reaction was heated at 100 °C for 4 h, then allowed to cool to room temperature and diluted with water (5 mL) and DCM (15 mL). The phases were separated, and the aqueous fraction was extracted with DCM (3 × 3 mL). The organic fractions were combined, dried over anhydrous Na_2_SO_4_ and the solvent was removed under reduced pressure. The crude material was purified twice by flash chromatography (0–5% v/v MeOH in DCM) to yield compound **5f** as a white solid (26 mg, 0.08 mmol, 33%). *R*_f_ 0.08 (5% v/v MeOH in DCM); ^1^H NMR (500 MHz, MeOD) δ 8.41 (d, *J* = 6.2 Hz, 2H), 7.81 (d, *J* = 8.4 Hz, 2H), 7.53 (d, *J* = 0.8 Hz, 1H), 7.33 (m, *J* = 8.6 Hz, 3H), 7.28 (d, *J* = 6.1 Hz, 2H), 7.20 (d, *J* = 3.1 Hz, 1H), 7.12 (dd, *J* = 8.4, 1.6 Hz, 1H), 6.40 (dd, *J* = 3.1, 0.9 Hz, 1H), 4.64 (s, 2H), 4.08 (s, 2H) ppm; ^13^C NMR (125 MHz, MeOD) δ 169.7, 152.7, 150.1, 144.3, 137.0, 134.3, 130.4, 130.3, 129.5, 128.9, 126.0, 125.9, 122.4, 120.4, 112.2, 102.3, 45.2, 41.7 ppm; IR (solid) *v*_max_ 3319, 3126, 3029, 2926, 2845, 1651, 1630, 1600, 1546, 1504, 1480, 1417, 1332, 1298, 1240, 1218, 1194, 1098, 1001, 986, 893, 877, 779, 757, 723, 663 cm^–1^; LCMS (+ESI) *m*/*z* 342.3 [M + H]^+^, retention time 1.55 min, (100%); HRMS (+ESI) *m*/*z* (Calcd C_22_H_20_N_3_O [M + H]^+^, 342.1601), Obs. 342.1598 (δ 0.8 ppm).

### (*R*)-*N*-(1-Phenylethyl)-3-(pyridin-4-ylmethyl)­benzamide (**5h**)

4-Pyridinylboronic acid (56 mg, 0.46 mmol), benzyl chloride **7c** (105 mg, 0.38 mmol), and Na_2_CO_3_ (85 mg, 0.80 mmol) were combined in a microwave vessel and flushed with *N*_2_(*g*) for 5 min before the addition of Pd­(PPh_3_)_4_ (9 mg, 0.01 mmol). The reaction vessel was flushed with *N*_2_(*g*) for a further 2–3 min, and then a mixture of DME and water (2:1 v/v, 2 mL) was added, and the reaction was heated at 100 °C for 4 h. The reaction was then allowed to cool to room temperature, and diluted with water (5 mL) and DCM (5 mL). The phases were separated, and the aqueous fraction was extracted with DCM (3 × 3 mL). The organic fractions were combined, dried over anhydrous Na_2_SO_4_ and the solvent was removed under reduced pressure. The crude material was purified twice by flash chromatography (0–5% v/v MeOH in EtOAc, then 30–100% v/v EtOAc in DCM)). The product containing fractions were combined after each successive purification to yield compound **5h** as a colorless amorphous solid (73 mg, 0.23 mmol, 61%). *R*_f_ 0.34 (5% v/v MeOH in EtOAc); ^1^H NMR (500 MHz, MeOD) δ 8.41 (d, *J* = 5.7 Hz, 2H), 7.73–7.71 (m, 2H), 7.43–7.38 (m, 4H), 7.32 (app. t, *J* = 7.7 Hz, 2H), 7.29 (d, *J* = 5.1 Hz, 2H), 7.22 (t, *J* = 7.3 Hz, 1H), 5.23 (q, *J* = 7.1 Hz, 1H), 4.08 (s, 2H), 1.55 (d, *J* = 7.1 Hz, 3H) ppm; ^13^C NMR (125 MHz, MeOD) δ 169.4, 152.8, 150.1, 145.3, 140.9, 136.4, 133.3, 130.0, 129.5, 129.1, 128.0, 127.2, 126.8, 125.9, 50.7, 41.7, 22.2 ppm; IR (solid) *v*_max_ 3219, 3056, 3023, 2968, 2924, 2365, 1622, 1598, 1583, 1538, 1493, 1430, 1417, 1326, 1272, 1217, 1205, 1128, 1088, 1020, 999, 912, 791, 760, 698 cm^1^; LCMS (+ESI) *m*/*z* 317.1 [M + H]^+^, 1.57 min, (100%); HRMS (+ESI) *m*/*z* (Calcd C_21_H_21_N_2_O [M + H]^+^, 317.1654), *Obs.* 317.1641 (δ 4.1 ppm).

### (*R*)-*N*-(1-Phenylethyl)-4-(pyridin-4-ylmethyl)­benzamide (**5i**)

4-Pyridinylboronic acid (53 mg, 0.43 mmol), benzyl chloride **7d** (99 mg, 0.36 mmol), and Na_2_CO_3_ (80 mg, 0.76 mmol) were combined in a microwave vessel and flushed with *N*_2_(*g*) for 5 min before the addition of Pd­(PPh_3_)_4_ (8 mg, 0.01 mmol). The reaction vessel was flushed with *N*_2_(*g*) for a further 2–3 min, and then a mixture of DME and water (2:1 v/v, 2 mL) was added, and the reaction was heated at 100 °C for 4 h. The reaction was then allowed to cool to room temperature, and diluted with water (5 mL) and DCM (5 mL). The phases were separated, and the aqueous fraction was extracted with DCM (3 × 3 mL). The organic fractions were combined, dried over anhydrous Na_2_SO_4_ and the solvent was removed under reduced pressure. The crude material was purified twice by flash chromatography (0–5% v/v MeOH in EtOAc, and 30–100% v/v EtOAc in DCM). The product containing fractions were combined after each successive purification to yield compound **5i** as a colorless amorphous solid (78 mg, 0.25 mmol, 68%). *R*_f_ 0.29 (5% v/v MeOH in EtOAc); ^1^H NMR (500 MHz, MeOD) δ 8.41 (d, *J* = 5.8 Hz, 2H), 7.80 (d, *J* = 8.2 Hz, 2H), 7.38 (d, *J* = 7.1 Hz, 2H), 7.34–7.27 (m, 6H), 7.22 (t, *J* = 7.3 Hz, 1H), 5.23 (q, *J* = 7.1 Hz, 1H), 4.08 (s, 2H), 1.55 (d, *J* = 7.1 Hz, 3H) ppm; ^13^C NMR (125 MHz, MeOD) δ 169.3, 152.7, 150.1, 145.4, 144.3, 134.3, 130.2, 129.5, 128.9, 128.0, 127.2, 125.9, 50.7, 41.7, 22.3 ppm; IR (solid) *v*_max_ 3411, 3180, 3034, 2974, 2931, 2164, 1631, 1601, 1542, 1505, 1493, 1446, 1416, 1348, 1310, 1297, 1277, 1206, 1189, 1119, 1065, 1015, 1006, 876, 831, 754, 743, 695 cm^–1^; LCMS (+ESI) *m*/*z* 316.9 [M + H]^+^, retention time 1.55 min, (100%); HRMS (+ESI) *m*/*z* (Calcd C_21_H_21_N_2_O [M + H]^+^, 317.1648), *Obs*. 317.1649 (δ 0.1 ppm).

### *N*-((1*H*-Indol-5-yl)­methyl)-3-(pyridin-4-ylamino)­benzamide (**5g**)

EDC.HCl (131 mg, 0.69 mmol) and HOAt (106 mg, 0.78 mmol) were added to a stirred solution of **4e** (122 mg, 0.57 mmol) in dry DCM (10 mL). 5-(Aminomethyl)­indole (83 mg, 0.57 mmol) and DIPEA (199 μL, 1.14 mmol) were added to the solution, followed by anhydrous DMF (1 mL) to aid solubility. The reaction was allowed to stir for 48–72 h at room temperature, then diluted with EtOAc (100 mL) and washed with water (20 mL). The aqueous fraction was extracted with EtOAc (10 mL) and the combined organic fractions were washed with saturated NaHCO_3_ (10 mL) and brine (5 mL), before being dried over anhydrous Na_2_SO_4_ and concentrated under reduced pressure. The crude product was purified by flash chromatography (0–10% v/v MeOH in EtOAc) to yield compound **5g** as a pink amorphous solid (120 mg, 0.35 mmol, 61%). *R*_f_ 0.03 (10% v/v MeOH in EtOAc); ^1^H NMR (500 MHz, *d*_6_-DMSO) δ 11.01 (s, 1H), 8.99 (app. t, *J* = 6.0 Hz, 1H), 8.92 (s, 1H), 8.21 (d, *J* = 6.4 Hz, 1H), 7.72 (app. t, *J* = 1.9 Hz, 1H), 7.55 (dt, *J* = 7.7, 1.3 Hz, 1H), 7.48 (s, 1H), 7.42 (app. t, *J* = 7.8 Hz, 1H), 7.34–7.33 (m, 2H), 7.31 (app. t, *J* = 2.7 Hz, 1H), 7.08 (dd, *J* = 8.4, 1.6 Hz, 1H), 6.92 (d, *J* = 6.4 Hz, 1H), 6.38 (tt, *J* = 2.0, 0.9 Hz, 1H), 4.54 (d, *J* = 5.9 Hz, 2H) ppm; ^13^C NMR (125 MHz, *d*_6_-DMSO) δ 165.7, 150.2, 149.8, 140.7, 135.9, 134.9, 129.9, 129.3, 127.5, 125.5, 122.3, 121.0, 121.0, 118.7, 118.7, 111.2, 109.4, 100.9, 43.2 ppm; IR (solid) *v*_max_ 3300–2900, 3265, 3022, 2912, 1724, 1635, 1575, 1516, 1482, 1432, 1341, 1216, 1147, 1094, 1044, 995, 984, 814, 754, 727, 697 cm^–1^; LCMS (+ESI) *m*/*z* 343.1 [M + H]^+^, retention time 1.54 min, (100%); HRMS (+ESI) *m*/*z* (Calcd C_21_H_19_N_4_O [M + H]^+^, 343.1559), *Obs.* 343.1565 (δ 2.3 ppm).

### *N*-(4-(Pyridin-4-ylamino)­phenyl)­benzamide (**5b**)

Aniline **4b** (49 mg, 0.26 mmol) and DIPEA (45 μL, 0.26 mmol) were added to a stirred solution of benzoic acid (32 mg, 0.26 mmol) and HATU (99 mg, 0.26 mmol) in anhydrous DCM (3 mL) at 0 °C. The reaction was allowed to come slowly to room temperature and stirred for 24 h. When complete, the reaction was diluted with EtOAc (20 mL) and water (10 mL), made slightly basic with sat. NaHCO_3_. The phases were separated and the aqueous phase was extracted with EtOAc (3 × 10 mL). The organic fractions were combined, washed with brine (5 mL) and the solvent was removed under reduced pressure. The crude product was purified by flash chromatography (10–100% v/v EtOAc in DCM, then 5–15% v/v MeOH in EtOAc) to yield compound **5b** as an off-white solid (50 mg, 0.17 mmol, 66%). *R*_f_ 0.33 (10% v/v MeOH in DCM); ^1^H NMR (500 MHz, *d*_6_-DMSO) δ 10.23 (s, 1H), 8.72 (d, *J* = 1.8 Hz, 1H), 8.16 (d, *J* = 5.6 Hz, 2H), 7.95 (d, *J* = 7.1 Hz, 2H), 7.76 (d, *J* = 8.5 Hz, 2H), 7.59 (t, *J* = 7.3 Hz, 1H), 7.53 (app. t, *J* = 7.5 Hz, 2H), 7.19 (d, *J* = 8.8 Hz, 2H), 6.85 (d, *J* = 6.1 Hz, 2H) ppm; ^13^C NMR (125 MHz, *d*_6_-DMSO) δ 165.3, 150.5, 150.0, 136.0, 135.0, 134.3, 131.5, 128.4, 127.6, 121.5, 120.9, 108.8 ppm; IR (solid) *v*_max_ 3381, 3248, 3033, 2938, 1646, 1619, 1592, 1537, 1511, 1430, 1405, 1349, 1315, 1258, 1215, 1105, 995, 893, 814, 794, 714, 702, 688, 649 cm^–1^; LCMS (+ESI) *m*/*z* 290.2 [M + H]^+^, retention time 1.55 min, (100%); HRMS (+ESI) *m*/*z* (Calcd C_18_H_16_N_3_O [M + H]^+^, 290.1288), *Obs.* 290.1275 (δ 4.3 ppm).

### *N*-(4-(Methyl­(pyridin-4-yl)­amino)­phenyl)­benzamide (**5c**)

Aniline **8b** (60 mg, 0.30 mmol) and DIPEA (52 μL, 0.3 mmol) were added to a stirred solution of benzoic acid (37 mg, 0.3 mmol) and HATU (114 mg, 0.3 mmol) in anhydrous DCM (2.5 mL) at 0 °C. The reaction was allowed to come slowly to room temperature and stirred for 24 h. When complete, the reaction was diluted with EtOAc (20 mL) and water (10 mL), made slightly basic with sat. NaHCO_3_. The phases were separated, and the aqueous phase was extracted with EtOAc (2 × 5 mL). The organic fractions were combined, washed with brine (5 mL), and the solvent was removed under reduced pressure. The crude product was purified by flash chromatography (10–50% v/v EtOAc in PE, then 10% v/v MeOH in EtOAc) to yield compound **5c** as an amorphous brown solid (28 mg, 0.09 mmol, 31%). *R*_f_ 0.21 (10% v/v MeOH in DCM); ^1^H NMR (400 MHz, CDCl_3_) δ 8.19 (d, *J* = 6.1 Hz, 2H), 8.04 (s, br, 1H), 7.90 (d, *J* = 7.0 Hz, 2H), 7.73 (d, *J* = 8.8 Hz, 2H), 7.58 (tt *J* = 7.3, 1.3 Hz, 1H), 7.51 (t, *J* = 7.3 Hz, 2H), 7.23 (d, *J* = 8.8 Hz, 2H), 6.56 (d, *J* = 6.6 Hz, 2H), 3.33 (s, 3H); ^13^C NMR (100 MHz, CDCl_3_) δ 166.0, 154.3, 149.1, 142.2, 136.5, 134.8, 132.2, 129.0, 127.6, 127.2, 121.9, 108.3, 39.7 ppm; IR (solid) *v*_max_ 3219, 3054, 1645, 1592, 1529, 1502, 1406, 1363, 1313, 1243, 1223, 1137, 1102, 1072, 991, 880, 841, 807, 704, 669 cm^–1^; LCMS (+ESI) *m*/*z* 304.0, retention time 1.49 min, (100%); HRMS (+ESI) *m*/*z* (Calcd C_19_H_18_N_3_O [M + H]^+^, 304.1444), *Obs.* 304.1439 (δ 2.0 ppm).

### *N*-(4-(Pyridin-4-ylmethyl)­phenyl)­benzamide (**5a**)

4-(4-Aminophenyl)­pyridine **3d** (110 mg, 0.60 mmol) and DIPEA (105 μL, 1.2 mmol) were added to a stirred solution of benzoic acid (73 mg, 0.60 mmol) and HATU (228 mg, 0.60 mmol) in anhydrous DCM (3 mL) at 0 °C. The reaction was allowed to come slowly to room temperature and stirred for 24 h. When complete, the reaction was diluted with EtOAc (20 mL), water (10 mL), and made slightly basic with sat. NaHCO_3_. The phases were separated, and the aqueous phase was extracted with EtOAc (3 × 10 mL), the organic fractions were combined, washed with brine (5 mL), and the solvent was removed under reduced pressure. The crude product was purified by flash chromatography (10–100% v/v EtOAc in PE, then 5% v/v MeOH in EtOAc) to yield compound **5a** as a white solid (133 mg, 0.46 mmol, 77%). *R*_f_ 0.09 (2:1 v/v EtOAc:PE); ^1^H NMR (400 MHz, *d*_6_-DMSO) δ 10.22 (s, 1H), 8.46 (d, *J* = 6.1 Hz, 2H), 7.94 (m, 2H), 7.71 (d, *J* = 8.5 Hz, 2H), 7.58 (tt, *J* = 7.4, 1.4 Hz, 1H), 7.52 (dd, *J* = 7.7, 7.0 Hz, 2H), 7.25–7.22 (m, 4H), 3.94 (s, 2H) ppm; ^13^C NMR (100 MHz, *d*_6_-DMSO) δ 165.5, 150.3, 149.6, 137.6, 134.8, 131.5, 129.1, 128.4, 127.6, 124.0, 120.6, 38.3 ppm; IR (solid) *v*_max_ 3362, 3044, 2916, 1655, 1597, 1579, 1525, 1491, 1411, 1323, 1311, 1259, 1227, 1184, 1104, 1072, 1026, 1006, 858, 829, 815, 793, 775, 741, 712, 690, 672 cm^–1^; LCMS (+ESI) *m*/*z* 289.2 [M + H]^+^, 1.90 min, (100%); HRMS (+ESI) *m*/*z* (Calcd C_19_H_17_N_2_O [M + H]^+^, 289.1335), *Obs.* 289.1322 (δ 4.7 ppm).

### (*S*)-2-(Benzylamino)-3-(1*H*-indol-3-yl)-*N*-(4-(pyridin-4-ylmethyl)­phenyl)­propanamide (**5d**)

**Step 1:** Benzaldehyde (203 μL, 2.0 mmol) was added to a stirred suspension of *l* -tryptophan (408 mg, 2.0 mmol) and NaOH (84 mg, 2.1 mmol) in dry MeOH (5 mL), and allowed to stir at room temperature for 1 h. The reaction was then cooled to 0 °C and sodium borohydride (99 mg, 2.6 mmol) was added as a single portion. The reaction was allowed to come slowly to room temperature and stirred for 2 h and then concentrated under reduced pressure. The residue was diluted with water (5 mL) and brought to pH ∼ 5 using 1.5 M HCl solution. The resulting precipitate was collected under reduced pressure, washed with iced water (10 mL), ice cold MeOH (5 mL), and then dried under reduced pressure to yield benzyl- *l* -tryptophan as a white solid (486 mg, 1.65 mmol, 83%). ^1^H NMR (500 MHz, *d*_6_-DMSO) δ 10.9 (s, 1H), 7.49 (d, *J* = 7.9 Hz, 1H), 7.33 (d, *J* = 8.1 Hz, 1H), 7.29–7.24 (m, 5H), 7.15 (d, *J* = 2.3 Hz, 1H), 7.05 (ddd, *J* = 8.1, 6.9, 1.2 Hz, 1H), 6.95 (ddd, *J* = 7.9, 7.4, 0.9 Hz, 1H), 3.81 (d, *J* = 13.4 Hz, 2H), 3.69 (d, *J* = 13.4 Hz, 2H), 3.41 (t, *J* = 6.5 Hz, 1H), 3.12 (dd, *J* = 14.6, 6.2 Hz, 1H), 3.02 (dd, *J* = 14.6, 6.7 Hz, 1H) ppm; ^13^C NMR (125 MHz, *d*_6_-DMSO) δ 173.6, 138.0, 136.1, 128.4, 128.2, 127.4, 127.2, 123.7, 120.8, 118.4, 118.21, 111.3, 110.1, 61.2, 50.5, 27.9 ppm; IR (solid) *v*_max_ 3049, 2968, 2880, 2702–2452, 1596, 1551, 1529, 1517, 1497, 1431, 1417, 1348, 1232, 1222, 1147, 1090, 1065, 1022, 933, 805, 751, 691, 654 cm^–1^; LCMS (+ESI) *m*/*z* 295.2 [M + H]^+^, 1.46 min, (100%); HRMS (+ESI) *m*/*z* (Calcd C_18_H_18_N_2_O_2_Na [M + Na]^+^, 317.1260), *Obs.* 317.1267.

**Step 2:** Benzyl- *l* -tryptophan (147 mg, 0.50 mmol), *n*-methyl morpholine (121 μL, 1.10 mmol) and then PyBOP (260 mg, 0.50 mmol) were added in quick succession to a stirred solution of 4-(pyridin-4-ylmethyl)­aniline (92 mg, 0.5 mmol) in anhydrous DCM (2 mL). The reaction was allowed to stir at room temperature for 1 h and then anhydrous DMF (300 μL) was added to aid solubility. The reaction was stirred at room temperature for a further 4 h and then concentrated under reduced pressure. The residue was diluted with EtOAc (30 mL), washed with water (5 mL) and saturated NaHCO_3_ (5 mL), dried over anhydrous Na_2_SO_4_ and then the solvent was removed under reduced pressure. The crude product was purified by flash chromatography (0–5% v/v MeOH in DCM) and the product containing fractions were concentrated under reduced pressure. The resulting oil was redissolved in EtOAc (50 mL), washed with water (4 × 10 mL), and brine (10 mL), dried over anhydrous Na_2_SO_4_ and the solvent was removed under reduced pressure to yield compound **5d** as a yellow amorphous solid (168 mg, 0.37 mmol, 73%). *R*_f_ 0.08 (2:1 v/v EtOAc:PE); ^1^H NMR (500 MHz, CDCl_3_) δ 9.41 (s, 1H, NH), 8.50 (d, *J* = 5.0 Hz, 2H), 8.47 (d, *J* = 4.6 Hz, 1H, NH), 8.11 (br s, 1H, NH), 7.66 (d, *J* = 7.8 Hz, 1H), 7.53 (d, *J* = 8.5 Hz, 1H), 7.38 (d, *J* = 8.2 Hz, 1H), 7.26–7.19 (m, 3H), 7.14 (d, *J* = 8.4 Hz, 2H), 7.12–7.09 (m, 3H), 7.06 (dd, *J* = 7.4, 2.0 Hz, 1H), 7.01 (d, *J* = 2.4 Hz, 1H), 6.96 (d, *J* = 8.4 Hz, 1H), 6.65 (d, *J* = 8.4 Hz, 1H), 3.95 (s, 2H), 3.76 (d, *J* = 13.4 Hz, 1H), 3.63 (d, *J* = 13.8 Hz, 1H), 3.61 (m, 1H), 3.44 (ddd, *J* = 14.7, 4.1, 1.0 Hz, 1H), 3.04 (dd, *J* = 14.8, 9.4 Hz, 2H) ppm; ^13^C NMR (125 MHz, CDCl_3_) δ 172.4, 150.3, 149.9, 145.1, 139.1, 136.6, 136.5, 134.6, 130.1, 129.7, 128.7, 128.0, 127.6, 127.4, 124.3, 123.0, 122.6, 120.0, 119.9, 119.0, 115.5, 111.4, 63.1, 53.0, 40.8, 29.1 ppm; IR (solid) *v*_max_ 3431, 3321, 3193, 3028, 2921, 2853, 1665, 1632, 1601, 1515, 1496, 1454, 1412, 1342, 1295, 1234, 1180, 1108, 1067, 999, 919, 846, 811, 738, 697 cm^–1^; LCMS (+ESI) *m*/*z* 461.4 [M + H]^+^, 1.45 min, (100%); HRMS (+ESI) *m*/*z* (Calcd C_30_H_29_N_4_O [M + H]^+^, 461.2336), *Obs*. 461.2269 (δ 1.3 ppm).

### *N*-(4-(Pyridin-4-ylmethyl)­phenyl)­benzenesulfonamide (**5j**)

Benzenesulfonyl chloride (77 μL, 0.6 mmol) was added to a solution of 4-(4-aminobenzyl)­pyridine **3d** (110 mg, 0.6 mmol) in anhydrous pyridine (2 mL) and the reaction was stirred overnight at room temperature. When complete, the reaction was diluted with DCM (25 mL) and water (10 mL), the phases were separated, and the aqueous phase was extracted with DCM (3 mL). The organic fractions were combined, dried over anhydrous Na_2_SO_4_ and the solvent was removed under reduced pressure. The crude product was purified by flash chromatography (0–100% v/v EtOAC in PE, then 0–10% v/v MeOH in EtOAc) to yield compound **5j** as a white solid (95 mg, 0.29 mmol, 49%). *R*_f_ 0.09 (2:1 v/v EtOAc:PE); ^1^H NMR (400 MHz, *d*_6_-DMSO) δ 10.22 (s, 1H), 8.42 (d, *J* = 6.0 Hz, 2H), 7.73 (m, 2H), 7.59 (tt, *J* = 7.5, 1.2 Hz, 1H), 7.53 (app. t, *J* = 7.5 Hz, 2H), 7.15 (d, *J* = 5.9 Hz, 2H), 7.09 (d, *J* = 8.4 Hz, 2H), 7.01 (d, *J* = 8.5 Hz, 2H), 3.84 (s, 2H) ppm; ^13^C NMR (100 MHz, *d*_6_-DMSO) δ 149.9, 149.6, 139.5, 136.0, 135.3, 132.9, 129.6, 129.2, 126.6, 124.0, 120.5, 39.4 ppm; IR (solid) *v*_max_ 3063, 3020, 2829, 2654, 1677, 1604, 1558, 1508, 1445, 1425, 1326, 1304, 1290, 1230, 1219, 1161, 1092, 1068, 1007, 964, 922, 849, 804, 787, 755, 723, 714, 700, 689 cm^–1^; LCMS (+ESI) *m*/*z* 325.2 [M + H]^+^, retention time 1.40 min, (95%); HRMS (+ESI) *m*/*z* (Calcd C_18_H_17_N_2_O_2_S [M + H]^+^, 325.1005), *Obs*. 325.0994 (δ 3.4 ppm).

### *N*-(4-(Pyridin-4-ylmethyl)­phenyl)-4-(trifluoromethoxy)­benzenesulfonamide (**5k**)

4-(Trifluoromethoxy)­benzenesulfonyl chloride (51 μL, 0.44 mmol) was added to a stirred solution of 4-(pyridin-4-ylmethyl)­aniline (74 mg, 0.40 mmol) and Et_3_N (112 μL, 0.80 mmol) in dry DCM (3 mL). The reaction was stirred at room temperature for 20 h and then diluted with DCM (20 mL) and washed with water (5 mL). The aqueous phase was extracted with DCM (2 mL) and the combined organic fractions were washed with brine (2 mL). The solvent was removed under reduced pressure and the crude product was purified by flash chromatography (20–80% v/v EtOAc in PE) to yield compound **5k** as a pale pink solid (46 mg, 0.11 mmol, 28%). *R*_f_ 0.10 (50% v/v EtOAc in PE); ^1^H NMR (500 MHz, CDCl_3_) δ 8.49 (d, *J* = 6.0 Hz, 2H), 7.80 (d, *J* = 9.0 Hz, 2H), 7.25 (d, *J* = 7.5 Hz, 2H), 7.14 (s, 1H), 7.08–7.02 (m, 6H), 3.91 (s, 2H) ppm; ^13^C NMR (125 MHz, CDCl_3_) δ 152.5, 150.0, 149.7, 137.6, 136.7, 134.8, 130.2, 129.5, 124.3, 122.6, 120.9, 119.3, 40.7 ppm; IR (solid) *v*_max_ 3009, 2924, 2645, 1606, 1563, 1510, 1489, 1421, 1332, 1258, 1212, 1154, 1095, 1008, 826, 808, 765, 708, 686, 625 cm^–1^; LCMS (+ESI) *m*/*z* 409.2 [M + H]^+^, 1.80 min, (100%); HRMS (+ESI) *m*/*z* (Calcd C_19_H_16_N_2_F_3_S [M + H]^+^, 409.0828), *Obs.* 409.0813 (δ 3.8 ppm).

### *N*-(4-Methoxybenzyl)-3-(pyridin-4-ylmethyl)­aniline (**5l**)

Glacial AcOH (0.7 mL) was added to a solution of 3-(pyridin-4-ylmethyl)­aniline **3c** (73 mg, 0.39 mmol) and *p*-anisaldehyde (88 μL, 0.75 mmol) in dry MeOH (5 mL). The reaction was stirred at room temperature for 30 min, and then NaCNBH_3_ (25 mg, 0.39 mmol) was added as a single portion. The reaction was stirred at room temperature for 24 h and then concentrated under reduced pressure. EtOAc (40 mL) and water (10 mL) were added, and the mixture was brought to pH 8 using saturated NaHCO_3_ solution. The phases were separated, and the aqueous fraction was extracted with EtOAc (2 × 10 mL). The combined organic fractions were washed with brine (5 mL), dried over anhydrous Na_2_SO_4_ and the solvent was removed under reduced pressure. The crude product was purified by flash chromatography (0–5% v/v MeOH in EtOAc) to yield compound **5l** as a yellow solid (51 mg, 0.17 mmol, 43%). *R*_f_ 0.22 (50% v/v EtOAc:PE); ^1^H NMR (500 MHz, CDCl_3_) δ 8.47 (d, *J* = 5.1 Hz, 2H), 7.26 (m, 2H), 7.11 (m, 3H), 6.87 (dd, *J* = 8.2, 1.5 Hz, 2H), 6.52 (dd, *J* = 8.3, 3.1 Hz, 2H), 6.41 (m, 1H), 4.22 (s, 2H), 3.86 (s, 2H) 3.78 (s, 3H) ppm; ^13^C NMR (125 MHz, CDCl_3_) δ 158.9, 150.5, 149.4, 148.5, 139.9, 131.1, 129.6, 128.8, 124.3, 118.2, 114.0, 113.4, 111.2, 55.3, 47.7, 41.4 ppm; IR (solid) *v*_max_ 3272, 3033, 2995, 2837, 1599, 1584, 1558, 1530, 1510, 1488, 1466, 1416, 1331, 1298, 1249, 1172, 1159, 1103, 1032, 997, 927, 854, 830, 813, 762, 748, 726, 690, 625, 605 cm^–1^; LCMS (+ESI) *m*/*z* 305.3, retention time 2.48 min, (100%); HRMS (+ESI) *m*/*z* (Calcd C_20_H_21_N_2_ O_1_ [M + H]^+^, 305.1648), *Obs.* 305.1642 (δ 2.2 ppm).

### *N*-(4-Methoxybenzyl)-4-(pyridin-4-ylmethyl)­aniline (**5m**)

Glacial AcOH (1 mL) was added to a solution of 4-(pyridin-4-ylmethyl)­aniline **3d** (138 mg, 0.75 mmol) and *p*-anisaldehyde (91 μL, 0.75 mmol) in dry MeOH (7.5 mL). The reaction was stirred at room temperature for 30 min, and then NaCNBH_3_ (47 mg, 0.75 mmol) was added as a single portion. The reaction was stirred at room temperature for 17 h and then concentrated under reduced pressure. DCM (10 mL) and water (10 mL) were added, and the mixture was brought to pH 8 using saturated NaHCO_3_ solution. The phases were separated, and the aqueous fraction was extracted with DCM (2 × 5 mL). The combined organic fractions were washed with brine (3 mL), dried over anhydrous Na_2_SO_4_ and the solvent was removed under reduced pressure. The crude product was purified by flash chromatography (25–100% EtOAc in DCM) to yield compound **5m** as a white solid (186 mg, 0.61 mmol, 82%). *R*_f_ 0.20 (2:1 v/v EtOAc:PE); ^1^H NMR (500 MHz, CDCl_3_) δ 8.47 (d, *J* = 6.1 Hz, 2H), 7.28 (d, *J* = 8.7 Hz, 2H), 7.09 (d, *J* = 6.1 Hz, 2H), 6.97 (d, *J* = 8.6 Hz, 2H), 6.88 (d, *J* = 8.6 Hz, 2H), 6.59 (d, *J* = 8.5 Hz, 2H), 4.24 (s, 2H), 3.85 (s, 2H), 3.80 (s, 3H) ppm; ^13^C NMR (125 MHz, CDCl_3_) δ 159.0, 151.1, 149.8, 147.1, 131.4, 130.0, 128.9, 127.8, 124.2, 114.2, 113.2, 55.4, 48.0, 40.5 ppm; IR (solid) *v*_max_ 3250, 3072, 3018, 2960, 2913, 2837, 1609, 1599, 1512, 1472, 1457, 1441, 1415, 1312, 1299, 1260, 1243, 1219, 1181, 1171, 1106, 1089, 1027, 997, 857, 817, 807, 777, 717 cm^–1^; LCMS (+ESI) *m*/*z* 305.3 [M + H]^+^, 1.72 min, (100%); HRMS (+ESI) *m*/*z* (Calcd C_20_H_21_N_2_O [M + H]^+^, 305.1648), *Obs.* 305.1644 (δ 1.4 ppm).

### *N*-(1-(4-Methoxyphenyl)­ethyl)-4-(pyridin-4-ylmethyl)­aniline (**5o**)

A 1 M solution of TiCl_4_ in DCM (1.3 mL, 1.3 mmol) was added to a solution of 4*′*-methoxyacetophenone (151 g, 1.0 mmol) in dry DCM (6 mL). The mixture was cooled to 0 °C and 4-(pyridin-4-ylmethyl)­aniline (372 mg, 2 mmol) was added. The reaction was then allowed to warm to room temperature and stir for 3 h before a methanolic solution of Na­(CN)­BH_3_ (185 μL of a 6.5 M solution, 1.2 mmol) was added. The reaction was stirred at room temperature for 24 h and then quenched with 2 M NaOH (until pH 10). The mixture was filtered, and the filtrate was partitioned between EtOAc (50 mL) and water (25 mL). The organic layer separated and washed with water (2 × 20 mL) and brine (20 mL), dried over anhydrous Na_2_SO_4_ and the solvent was removed under reduced pressure. The crude product was purified by flash chromatography (20–80% v/v EtOAc in DCM, followed by 0–5% v/v MeOH in EtOAC) to obtain compound **5o** as a yellow amorphous solid (88 mg, 0.28 mmol, 28%). *R*_f_ 0.26 (50% v/v EtOAc in Pet. ether); ^1^H NMR (400 MHz, CDCl_3_) δ 8.44 (d, *J* = 5.9 Hz, 2H), 7.27 (d, *J* = 8.8 Hz, 2H), 7.06 (d, *J* = 5.9 Hz, 2H), 6.89 (d, *J* = 8.4 Hz, 2H), 6.85 (d, *J* = 8.7 Hz, 2H), 6.46 (d, *J* = 8.5 Hz, 2H), 4.41 (q, *J* = 6.7 Hz, 1H), 3.98 (br s, 1H), 3.80 (s, 2H), 3.78 (s, 3H), 1.48 (d, *J* = 6.8 Hz, 3H) ppm; ^13^C NMR (100 MHz, CDCl_3_) δ 158.5, 151.0, 149.7, 146.1, 137.2, 129.7, 127.3, 126.9, 124.1, 114.0, 113.5, 55.3, 53.0, 40.4, 25.1 ppm; IR (solid) *v*_max_ 3307, 3035, 2960, 2833, 1610, 1586, 1559, 1509, 1460, 1437, 1415, 1364, 1321, 1286, 123, 1222, 1182, 1167, 1097, 1028, 1012, 1006, 995, 941, 911, 879, 851, 826, 808, 767, 741, 666 cm^–1^; LCMS (+ESI]) *m*/*z* 319.3, retention time 1.55 min, (100%); HRMS (+ESI) *m*/*z* (Calcd C_21_H_23_ON_2_ [M + H]^+^, 319.1810), *Obs.* 319.1815 (δ 1.6 ppm).

### *N*-(3-(Methylsulfonyl)­benzyl)-4-(pyridin-4-ylmethyl)­aniline (**5p**)

Glacial AcOH (1 mL) was added to a solution of 4-(pyridin-4-ylmethyl)­aniline **3d** (138 mg, 0.75 mmol) and 3-methylsulfonylbenzaldehyde (131 mg, 0.75 mmol) in dry MeOH (7.5 mL). The reaction was stirred at room temperature for 30 min, and then NaCNBH_3_ (47 mg, 0.75 mmol) was added as a single portion. The reaction was stirred at room temperature for 17 h and then concentrated under reduced pressure. DCM (10 mL) and water (10 mL) were added, and the mixture was brought to pH 8 using saturated NaHCO_3_ solution. The phases were separated, and the aqueous fraction was extracted with DCM (2 × 5 mL). The combined organic fractions were washed with brine (3 mL), dried over anhydrous Na_2_SO_4_ and the solvent was removed under reduced pressure. The crude product was purified by flash chromatography (25–100% EtOAc in DCM) to yield compound **5p** as a yellow solid (209 mg, 0.59 mmol, 79%). *R*_f_ 0.05 (2:1 v/v EtOAc:PE); ^1^H NMR (500 MHz, CDCl_3_) δ 8.46 (d, *J* = 6.0 Hz, 1H), 7.94 (s, 1H), 7.84 (ddd, *J* = 7.7, 2.0, 1.1 Hz, 1H), 7.66 (ddd, *J* = 7.6, 1.9, 1.0 Hz, 1H), 7.53 (app. t, *J* = 7.7 Hz, 1H), 7.08 (d, *J* = 6.0 Hz, 1H), 6.97 (d, *J* = 8.5 Hz, 2H), 6.55 (d, *J* = 8.5 Hz, 2H), 4.42 (d, *J* = 4.8 Hz, 2H), 4.21 (t, *J* = 5.7 Hz, 1H), 3.84 (s, 2H), 3.03 (s, 3H) ppm; ^13^C NMR (125 MHz, CDCl_3_) δ 150.9, 149.8, 146.3, 141.8, 141.1, 132.6, 130.1, 129.8, 128.5, 126.2, 126.0, 124.2, 113.3, 47.9, 44.5, 40.5 ppm; (solid) *v*_max_ 3251, 3027, 2916, 2869, 2845, 1611, 1603, 1560, 1519, 1477, 1416, 1316, 1291, 1261, 1220, 1181, 1142, 1094, 996, 960, 924, 865, 810, 778, 760, 686 cm^–1^; LCMS (+ESI) *m*/*z* 353.2 [M + H]^+^, 1.43 min, (100%); HRMS (+ESI) *m*/*z* (Calcd C_20_H_21_N_2_O_2_S [M + H]^+^, 353.1318), *Obs*. 353.1313 (δ 1.4 ppm).

### N^1^-(4-Methoxybenzyl)-N^4^-methyl-N^4^-(pyridin-4-yl)­benzene-1,4-diamine (**5n**)

Glacial AcOH (0.7 mL) was added to a solution of aniline **8b** (62 mg, 0.31 mmol) and *p*-anisaldehyde (73 μL, 0.62 mmol) in dry MeOH (5 mL). The reaction was stirred at room temperature for 30 min, and then NaCNBH_3_ (20 mg, 0.31 mmol) was added as a single portion. The reaction was stirred at room temperature for 24 h and then concentrated under reduced pressure. EtOAc (40 mL) and water (10 mL) were added, and the mixture was brought to pH 8 using saturated NaHCO_3_ solution. The phases were separated, and the aqueous fraction was extracted with EtOAc (2 × 10 mL). The combined organic fractions were washed with brine (5 mL), dried over anhydrous Na_2_SO_4_ and the solvent was removed under reduced pressure. The crude product was purified by flash chromatography (0–10% v/v MeOH in DCM) to yield compound **5n** as an off-white solid (49 mg, 0.15 mmol, 50%). *R*_f_ 0.32 (10% v/v MeOH in DCM); ^1^H NMR (500 MHz, CDCl_3_) δ 8.15 (d, *J* = 6.0 Hz, 2H), 7.31 (d, *J* = 8.7 Hz, 2H), 6.99 (d, *J* = 8.7 Hz, 2H), 6.91 (d, *J* = 8.7 Hz, 2H), 6.67 (d, *J* = 8.7 Hz, 2H), 6.47 (d, *J* = 6.6 Hz, 2H), 4.27 (s, 2H), 3.82 (s, 3H), 3.25 (s, 3H) ppm; ^13^C NMR (125 MHz, CDCl_3_) δ 159.1, 154.9, 148.9, 147.1, 135.9, 131.1, 128.9, 128.2, 114.2, 113.9, 107.8, 55.5, 48.0, 39.8 ppm; IR (solid) *v*_max_ 3291, 2826, 1642, 1608, 1593, 1535, 1508, 1471, 1442, 1372, 1322, 1303, 1243, 1222, 1181, 1114, 1035, 983, 872, 833, 807, 798, 759 cm^–1^; LCMS (+ESI) *m*/*z* 320.1, retention time 1.80 min (100%); HRMS (+ESI) *m*/*z* (Calcd C_20_H_22_N_3_O [M + H]^+^, 320.1757), Obs. 320.1751 (δ 2.0 ppm).

## Supplementary Material





## Notes

Supplementary file containing fragment screening results; structure-activity relationships of 1a analogues; X-ray crystallography refinement statistics and electron density maps in Fragmen Figures 1a and 3a-d; biochemical inhibition data, antimicrobial activity data; selectivity profiling against human P450s; reporter assays; protein purification, supplementary synthesis schemes; NMR spectra of final compounds; and representative LC-MS data (PDF)
Molecular strings table for reported compounds (CSV)

## Abbreviations

EtOAc: ethyl acetate
DIPEA: diisopropyl ethylamine
EDC: *N*-(3-(dimethylamino)­propyl)-*N*′-ethylcarbodiimide
GE: group efficiency
HBTU: O-(benzotriazol-1-yl)-*N*,*N*,*N*′,*N*′-tetramethyluronium hexafluorophosphate
HOAt: 1-hydroxy-7-azabenzotriazole
HPCD: (2-hydroxypropyl)-β-cyclodextrin
K_D_: dissociation constant
MABA: microplate alamar blue assay
MeCN: acetonitrile
Mtb: *Mycobacterium tuberculosis*
P450: cytochrome P450 enzyme
RLU: relative light units
XDR: extensively drug resistant
