# Supplementary material for: Fragment-Based Development of Small Molecule Inhibitors Targeting Mycobacterium tuberculosis Cholesterol Metabolism
Source: J Med Chem. 2025 Jul 14;68(14):14416–41. doi: 10.1021/acs.jmedchem.5c00478 (PMC12305662; doi:10.1021/acs.jmedchem.5c00478)

Supporting Information:

Fragment-based development of small molecule inhibitors targeting *Mycobacterium tuberculosis* cholesterol metabolism

Madeline E. Kavanagh,[1] Kirsty J. McLean,[2]* Sophie H. Gilbert[1], Cecilia N. Amadi[2], Matthew Snee[2], Richard B. Tunnicliffe[2], Kriti Arora[3], Helena I. M. Boshoff[3], Alexander Fanourakis[1], Maria Jose Rebollo-Lopez[4], Fatima Ortega[4], Colin W. Levy[5], Andrew W. Munro[2], David Leys[6], Chris Abell[1] and Anthony G. Coyne[1]*

[1]Yusuf Hamied Department of Chemistry, University of Cambridge, Lensfield Road, Cambridge, CB2 1EW, UK. [2]Centre for Synthetic Biology of Fine and Specialty Chemicals (SYNBIOCHEM), Manchester Institute of Biotechnology, University of Manchester, 131 Princess Street, Manchester, M1 7DN, UK. [3]Tuberculosis Research Section, Laboratory of Clinical Immunology and Microbiology, National Institutes of Health, Bethesda, Maryland, 20892, USA. [4]Global Health R&D, GSK, Severo Ochoa 2, Tres Cantos, 28760, Spain. [5]Manchester Protein Structure Facility (MPSF), Manchester Institute of Biotechnology, University of Manchester, Manchester, M1 7DN, UK. [6]Department of Chemistry, Manchester Institute of Biotechnology, University of Manchester, 131 Princess Street, Manchester, M1 7DN, UK

*corresponding authors. Contact: m.e.kavanagh@lic.leidenuniv.nl; k.j.mclean@hud.ac.uk

Contents

Table S1. Fragment screen against purified *Mtb* P450s by UV-vis spectroscopy…………………………….. 2
Table S2. Structure-activity relationships of benzylpyridine **1a** analogues…………………………………... 4
Table S3. X-ray crystallography data and refinement statistics………………………………………………. 5
Table S4. Inhibition of CYP125 and CYP142 catalytic activity *in vitro*……………………………………... 6
Table S5. Antibacterial activity of CYP125/142 ligands against extracellular *Mtb* (H37Rv) on cholesterol… 7
Table S6. Activity of CYP125/142 inhibitors against multi-drug resistant *Mtb*……………………………….. 8
Table S7. Antibacterial activity of CYP125/142 inhibitors against H37Rv *Mtb* cultured on glucose or glycerol…… 9
Table S8. Inhibition of human liver microsomal P450s………………………………………………………. 10
Figure S1. Compound **5g** induces a unique conformation in the CYP125 active site………………………... 11
Figure S2. Electron density maps for ligands shown in Figures 1a and 3a-d………………………………… 12
Figure S3. Transcriptional reporter assays to determine **5m** mechanisms of action ………………………… 13
Figure S4. Non-specific protein binding reduces extracellular antitubercular activity…..…………………… 14
Figure S5. Purification and crystallization of recombinant CYP125 and CYP142…………………………… 14
Scheme S1. Synthesis of **1a-i** analogue to explore SAR of benzylic position……………………………... 15
Scheme S2. Synthesis of **1a-i** analogues to explore SAR of "linker"……………………………………… 15
Scheme S3. Synthesis of **2a** analogues to explore SAR of "*linker*"………………………………………... 15
NMR spectra of final compounds tested in antitubercular assays……………………………………………... 16
Representative LC-MS data……………………………………………………………………………………. 33

Table S1. Fragment screen against purified *Mtb* P450s by UV-vis spectroscopy. Interaction between fragment (1 mM) and P450 (4-6 μM) was quantified from the shift in the Soret band ($\Delta\lambda_{max}$, nm) of each enzyme's absorbance spectrum, relative to DMSO. NB: All P450s tested are predominantly low-spin at resting state (λ_{max} (DMSO) ~ 416 – 421 nm), except CYP125, which is predominantly high spin (λ_{max} (DMSO) = 393 nm). Consequently, fragments that bind and stabilize the P450 low-spin state induce a significantly larger $\Delta\lambda_{max}$ in the spectrum of CYP125 compared to the other P450s analyzed. ND: not determined, due to optical interference, or insolubility in P450-optimized buffer.

Fragment	SMILES	CYP125	CYP142	CYP124	CYP121	CYP126	CYP143	CYP144
	Soret λ_{max} (nm) - DMSO	393	418.5	419	417	418	416	421
1	OC(=O)c1ccnnc1	0	0	0	-1	0	0.5	0
2	C1(C2=CC=NN2)=CC=CC=N1	0	0	0	0	0	0	0
3	NC1=NC2C(C=CC=C2)=N1	0	0	0	0	0	0.5	0
4	C1(C2=CC=CC=C2)=NC=CN1	0	0	0	0	0	0	0
5	c1ncnn1Cc1cc(ccc1)N	0	1.5	1	2	0	1	0
6	Nc1n(c2ccccc2)nc(C)c1	0	0	0	0	0	0.5	0
7	CC(C=C1)=NNC1=O	0	0	0	0	0	0	0
8	c1(c2ccncc2)ccc(cc1)CO	25	0	1	1	3	1	0
9	n1c(cc(n1[H])c1occc1)CO	0	0	-1	0	0	0.5	0
10	n1(c(ncc1)CO)Cc1ccccc1	0	3	1	0	0	1	0
11	n1n(c(cc1c1ccccc1)N)C	0	0	0	0	0	-0.5	0
12	NC1=CC(C2=CC=C(C=C2)Cl)=NN1	-2	0	-1	2	1	0	0
13	N1(/N=C(\CC1=O)/N)c1ccccc1	0	0	-1	0	0	0	0
14	CC1=C(C=NN1)C2=CC=CC=C2	-2	1.5	3	0	0	0	0
15	c1cnc(cc1)c1cccc(c1)C(O)=O	0	0	0	-1	0	0	0
16	NC1=CC=C(N2N=C(C)C=C2C)C=C1	0	0	0	-1	0	0.5	0
17	s1c(ccc1c1ncccc1)C(=O)O	0	0	0	-1	0	0.5	0
18	n1(nc(c(n1)C)C(=O)O)c1ccccc1	0	0	0	0	0	0	0
19	Clc1cncc(Cl)c1	0	0	0	0	0	0	0
20	c1ccc(cc1)Cc1ccncc1	28	3	1	2	1	2	0
21	CNCc1cccc(c1)c1cccnc1	25	3	-1	0	1	4	0
22	OC(=O)c1ccccc1n1cccn1	0	0	0	0	0	0	0
23	Nc1ccc(Br)cn1	0	0	0	-1	0	0.5	0
24	Oc1nnc(O)c(C)c1	0	0	0	-1	0	0	0
25	COC(=O)c1ccnc(N)c1	0	0	0	0	0	0.5	0
26	COC(=O)c1ncc2ccccc2c1	0	0	0	0	0	1	0
27	Clc1ccc(Cl)nn1	0	0	0	0	0	0.5	0
28	Nc1cc(Cl)nc(Cl)c1	0	0	0	0	0	0	0
29	OCc1ccc(Br)cn1	0	0	0	0	0	0.5	0
30	Oc1cccc(n1)C(=O)O	0	0	0	0	0	1	0
31	COc1ccc(cn1)C(=O)C	0	0	0	0	0	0.5	0
32	Cc1cc(C(=O)O)[nH]n1	0	0	0	0	0	-0.5	0
33	OB(O)c1cccnc1	0	0	0	0	0	4	0
34	OC(=O)CCc1nc2ccccc2[nH]1	0	0	0	0	0	0.5	0
35	c1ccc(cc1)c1n[nH]cc1	-2	0	-1	0	0	1	0
36	OC(=O)CCc1cccnc1	0	0	0	0	2	1	0
37	Brc1ccc2ccnc(Cl)c2c1	0	0	0	-3	0	ND	0
38	Brc1c[nH]nc1C	0	1.5	0	0	0	0	0

39	N1CCc2ccccc2C1	0	1.5	2	0	0	1	0
40	N#Cc1ccccn1	0	0	0	0	0	0.5	0
41	N#Cc1ccncc1	0	0	0	0	0	-0.5	0
42	Oc1ncccc1O	0	0	-1	-1	0	0.5	0
43	Nc1ccccn1	0	0	0	0	0	1	0
44	Oc1ccccn1	0	0	0	0	0	-0.5	0
45	Nc1cccnc1O	0	0	0	ND	0	0	0
46	CC(C)c1n[nH]c(c1)C(=O)O	0	0	0	0	0	-0.5	0
47	Nc1ccncc1	0	1.5	0	0	0	0	0
48	Nc1ccc(nc1)Oc1ccccc1	0	1.5	-1	1	2	0.5	0
49	Nc1ccc(cc1)n1cccn1	0	0	0	0	2	1	0
50	COc1ncccc1N	0	0	0	0	0	2.5	0
51	OC(=O)c1ccnn1c1ccccc1	0	0	0	-1	0	0.5	0
52	CNCc1ccc(cc1)c1ccncc1	28.5	1.5	2	-1	0	4	0
53	OC(=O)c1ccc(cc1)c1cccnc1	0	0	0	0	0	1	0
54	OC(=O)c1ccc(cc1)n1cccn1	0	0	0	0	-1	-0.5	0
55	[O-][N+](=O)c1cccnc1N	ND	0	0	ND	0	ND	0
56	Clc1nccc2ccccc12	0	0	0	-2	0	-0.5	0
57	Brc1cccnc1Cl	0	0	0	-1	0	0.5	0
58	Brc1ccc(=O)[nH]c1	0	0	0	0	-1	0.5	0
59	[O-][N+](=O)c1ccc(Br)c2ccncc12	0	0	1	ND	0	ND	0
60	OC(=O)c1cc(F)cnc1	0	0	0	0	0	1	0
61	Clc1ncccc1Cl	ND	0	0	0	0	0	0
62	OC(=O)c1cccnc1N	ND	0	0	ND	0	4	0
63	c1ccc(nc1)N1CCOCC1	0	0	0	0	0	1	0
64	O=C1CCN(N1)c1ccccc1	0	1.5	-1	0	0	1	0
65	COc1ncccc1C(=O)O	ND	0	0	0	0	1	0
66	Nc1ncccc1N	ND	0	0	ND	0	0	0
67	CCc1[nH]cc(C)n1	0	0	0	-1	0	0	0
68	c1ccc(cc1)c1c[nH]cn1	0	3	4	0	1	2	0
69	OCc1ccc(cc1)Cn1ncnc1	0	0	0	0	0	0	0
70	OC(=O)c1cc(c2ccccc2)n(C)n1	0	0	2	0	0	0.5	0
71	N#Cc1cccc(c1)n1cncc1	0	3	1	0	0	2.5	0
72	N#Cc1ncccc1F	0	0	0	0	0	0	0
73	Nc1ccc(Cl)nn1	0	0	0	0	0	0	0
74	OC(=O)c1cccc(c1)c1cccnc1	0	0	0	0	0	1	0
75	C#Cc1cccnc1	0	0	0	0	0	0	0
76	OC(=O)c1cc(O)c2ccccc2n1	0	0	0	0	0	0.5	0
77	Brc1c[nH]cn1	0	3	0	-1	0	0.5	0
78	OS(=O)(=O)c1ccccn1	0	0	0	0	0	1	0
79	OC(=O)[C@H]1NCc2ccccc2C1	-2	0	0	1	0	0	0
80	Cc1ccc(nc1)S(=O)(=O)N	0	0	0	0	0	0	0

Table S2. Structure-activity relationships of benzylpyridine **1a** analogues. Compound (100 µM) binding to purified P450 (5 µM) was quantified from the shift in the Soret band ($\Delta\lambda_{max}$, nm) of each enzyme's absorbance spectrum, relative to DMSO. [a]CYP125 is prominently high spin (HS) at resting state (λ_{max} ~ 393 nm),$\Delta\lambda_{max}$ values was calculated for the $\Delta\lambda_{max}$ of both the HS and low spin (LS) enzyme populations represented in the absorbance spectrum. [b]The ratio of LS/HS CYP125 provides additional indication of the extent to which compound binding stabilizes the LS, presumably inactive, state. [c]No LS or no HS maxima was present in the enzyme spectrum. ND – value not determined.

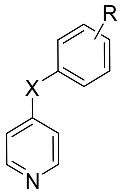

Compound	X	R	CYP125 $\Delta\lambda_{max}$ (nm) HS[a]	CYP125 $\Delta\lambda_{max}$ (nm) LS	CYP125 LS/HS[b]	CYP142 $\Delta\lambda_{max}$ (nm)
**1a**	CH_2	H	2	27	1.1	2
**2a**	NH	H	0	-[c]	ND	4
**2b**	NHMe	H	1	25	0.9	4
**2c**	NHPh	H	2	28	1	4
**2d**	O	H	1	25	1	2
**2e**	SO_2	H	0	-	ND	0
**2f**	C=O	H	0	-	ND	0
**2g**	*E*-CH=CH	H	-	29	1.4	3
**2h**	CH_2CH_2	H	2	26	1	2
**3a**	CH_2	3-OMe	1	27	1.1	2
**3b**	CH_2	4-OMe	1	25	1	2
**3d**	CH_2	3-NH_2	3	29	1.3	2
**3e**	CH_2	4-NH_2	3	29	1.7	3
**3f**	CH_2	4-NHCOMe	-	13	ND	2
**3g**	CH_2	4-$NHSO_2Me$	-	30	2.7	0
**4a**	NH	3-NH_2	1	22	0.9	3
**4b**	NH	4-NH_2	1	31	1.3	3
**4c**	NH	3-CO_2Me	1	27	1	3
**4d**	NH	4-CO_2Me	2	26	1	3
**4e**	NH	3-CO_2H	0	-	ND	0
**4f**	NH	4-CO_2H	1	-	ND	0
**4g**	NH	3-CH_2OH	0	-	ND	1
**4h**	NH	3-Br	-	31	1.4	4
**4i**	NH	4-Br	2	29	1.2	4

Table S3. X-ray crystallography data and refinement statistics.

	**CYP125-5m (PDB 7ZIC)**	**CYP125-5j (PDB 7ZGL)**	**CYP125-5g (PDB 8S4M)**	**CYP142-1a (PDB 8S53)**	**CYP142-5m (PDB 7P5T)**	**CYP142 -5j (PDB 7QQ7)**
**Data collection**						
Space group	C 1 2 1	C 1 2 1	C1 2 1	P $2_1 2_1 2_1$	P $2_1 2_1 2_1$	P $2_1 2_1 2_1$
Cell dimensions						
a, b, c (Å)	136.23, 68.68, 144.47	136.31, 69.33, 144.89	136.90, 68.85, 144.12	55.46, 65.77, 130.66	55.73, 65.72, 129.07	55.31, 65.19, 128.41
α, β, γ (°)	90,00 93.97, 90.00	90.00, 94.41, 90.00	90.00, 93.92, 90.00	90.00, 90.00, 90.00	90.00, 90.00, 90.00	90.00, 90.00, 90.00
Resolution (Å)	1.90	2.50	2.1	1.6	1.30	1.60
No. reflections (total)	348123 (16169)	156569 (15019)	260955 (15185)	490509 (12064)	663389 (12279)	410853 (21072)
No. reflections (unique)	104289 (5050)	46975 (4600)	78300 (4463)	62563 (2491)	116304 (5347)	62174 (3085)
R_{merge}	0.064 (0.898)	0.148 (0.908)	0.071 (0.861)	0.062 (0.831)	0.053 (0.705)	0.072 (0.795)
I / σI	9.2 (1.2)	4.6 (1.1)	9.1 (1.4)	17 (1.3)	11.4 (1.0)	12.1 (2.2)
CC 1/2	0.996 (0.518)	0.969 (0.599)	0.995 (0.585)	0.998 (0.657)	0.998 (0.566)	0.998 (0.841)
Completeness (%)	99.4 (99.0)	99.9 (99.9)	99.9 (100)	97.9 (79.9)	99.28 (99.10)	100.00 (100.00)
Multiplicity	3.3 (3.2)	3.3 (3.3)	3.3 (3.4)	7.8 (4.8)	5.7 (2.3)	6.6 (6.5)
**Refinement**						
Rwork / Rfree	0.188 / 0.224	0.210 / 0.251	0.212 / 0.246	0.156/0.175	0.137 / 0.162	0.164 / 0.183
R.m.s. deviations						
Bond lengths (Å)	0.007	0.004	0.003	0.012	0.009	0.009
Bond angles (°)	0.801	0.651	0.619	1.154	1.41	1.072

Table S4. Inhibition of CYP125 and CYP142 catalytic activity *in vitro*. The concentration of compound to inhibit 50% of CYP125 (0.5 µM) or CYP142 (1 µM) catalyzed turnover of cholest-4-en-3-one (5 µM) (IC_{50} value) was quantified by LC-MS. Inhibition equilibrium constants (K_I) were estimated by Cheng-Prusoff method using cholest-4-en-3-one K_m CYP125 = 2.1 µM, CYP142 = 0.36 µM. "-" – not determined. Compound structures shown below.

	CYP125A1		CYP142A1	
**Compound**	**IC_{50} (µM)**	**K_I (µM)**	**IC_{50} (µM)**	**K_I (µM)**
**1a**	-	-	-	-
**2a**	-	-	-	-
**3f**	-	-	-	-
**5a**	4.3 ± 0.5	1.3	-	-
**5b**	-	-	-	-
**5c**	26 ± 2.4	7.7	-	-
**5d**	0.79 ± 0.09	0.23	2.5 ± 2.4	0.17
**5e**	9.2 ± 0.89	2.7	33 ± 3.4	2.2
**5f**	3.6 ± 0.41	1.1	-	-
**5g**	25 ± 2.4	7.1	34 ± 3.4	2.3
**5i**	33 ± 3.7	9.8	-	-
**3g**	-	-	-	-
**5j**	1.5 ± 0.16	0.44	2.3 ± 0.30	0.15
**5k**	0.91 ± 0.22	0.27	3.6 ± 0.36	0.24
**5l**	18 ± 1.9	5.4	16 ± 1.6	1.1
**5m**	0.35 ± 0.04	0.10	0.67 ± 0.07	0.05
**5n**	12 ± 1.2	3.6	-	-
**5o**	4.2 ± 0.48	1.2	6.1 ± 0.60	0.41
**5p**	10 ± 0.95	3.1	22 ± 2.4	1.5

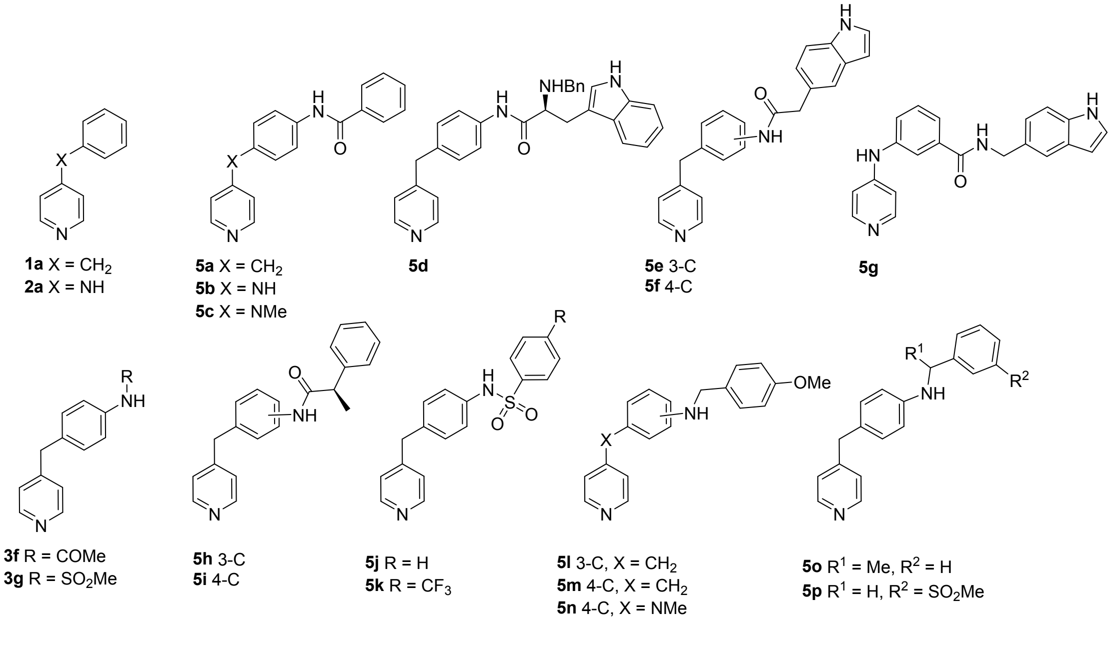

Table S5. Antibacterial activity of CYP125/142 ligands against extracellular *Mtb* on cholesterol. *Mtb* (H37Rv or CDC1551) was cultured in media that contained cholesterol as the only source of carbon and treated with compounds (0-50 µM) for 1-2 weeks. Growth inhibition was quantified as the concentration of compound required to reduce resazurin reduction (MABA) by 99% relative to DMSO control (MIC_{99}), or ATP-dependent luminescence (ATP) by 50% relative to DMSO treated controls (IC_{50}) in independent replicate experiments. "-" – not determined.

	**MABA-MIC$_{99}$ (W2) (µM)**		**ATP-IC$_{50}$ (W1) (µM)**		**ATP- IC$_{50}$ (W2) (µM)**	
**Compound**	**H37Rv**	**CDC1551**	**H37Rv**	**CDC1551**	**H37Rv**	**CDC1551**
**1a**	-	-	-	-	-	-
**2a**	-	-	-	-	-	-
**3f**	-	-	-	-	-	-
**5a**	25	>50	19	19	38	38
**5b**	-	-	-	-	-	-
**5c**	>50	-	9.4	-	19	-
**5d**	25	25	4.7	4.7	4.7	38
**5e**	13	6.3	2.3	0.07	19	19
**5f**	>50	>50	>50	>50	>50	>50
**5g**	13	25	4.7	9.4	9.4	38
**5h**	25	25	9.4	2.3	19	38
**5i**	25	25	9.4	2.3	4.7	38
**3g**	-	-	-	-	-	-
**5j**	50	25	38	9.4	50	>50
**5k**	25	25	2.3	1.2	2.3	9.4
**5l**	19	-	0.59	-	2.3	-
**5m**	1.5	13	0.15	0.59	1.2	9.4
**5n**	50	-	4.7	-	9.4	-
**5o**	19	-	0.15	-	0.59	-
**5p**	13	13	1.2	2.3	4.7	19
**p-AS**	0.19	0.39	0.04	0/04	0.29	2.3
**Isoniazid**	<0.1	-	<0.1	-	<0.1	-

Table S6. Activity of CYP125/142 inhibitors against multi-drug resistant *Mtb*. Inhibition of drug susceptible H37Rv *Mtb* or isoniazid and rifampicin resistant MDR-TB (K26b00MR 113) growth by 90% (MIC_{90}, µM) was calculated from the difference in resazurin reduction (MABA) relative to DMSO-treated controls 1- and 2-weeks post-compound treatment in replicate experiments.

	Week 1		Week 2	
**Compound**	**H37Rv**	**MDR-TB**	**H37Rv**	**MDR-TB**
**5m**	0.78	0.39	6.25	12.5
Isoniazid	0.19-0.39	12.5-25	0.39	50

Table S7. Antibacterial activity of CYP125/142 inhibitors against H37Rv *Mtb* cultured on glucose or glycerol. The concentration of compound required to inhibit the growth of H37Rv *Mtb* on media that contained either glucose or glycerol as the sole source of carbon by 50% (IC_{50}) was determined at 2 time points post-compound treatment from either the relative reduction in ATP-dependent luminescence or resazurin reduction (MABA) relative to DMSO treated controls. "-" – not determined.

	**Glucose Media**				**Glycerol Media**	
	**ATP IC_{50} (μM) (W1)**		**ATP IC_{50} (μM) (W2)**		**MABA IC_{50} (μM)**	
**Compound**	**H37Rv**	**CDC1551**	**H37Rv**	**CDC1551**	**10-days**	**21 days**
**5a**	19	19	50	38	-	-
**5c**	19	-	>50	-	-	-
**5d**	4.7	19	2.3	38	-	-
**5e**	1.2	19	9.4	19	-	-
**5g**	2.3	9.4	4.7	38	-	-
**5h**	1.2	19	19	38	-	-
**5i**	9.4	19	19	38	-	-
**5j**	38	38	19	38	-	-
**5k**	19	19	4.7	9.4	-	-
**5l**	9.4	-	2.3	-	-	-
**5m**	1.2	4.7	2.3	9.4	3.5	19
**5n**	9.4	-	19	-	-	-
**5o**	2.3	-	2.3	-	-	-
**5p**	2.3	19	4.7	19	-	-
p-AS	<0.04	<0.04	0.07	0.07	-	-
Isoniazid	<0.1		0.24		-	-
Pyrazinamide	-		-		0.9	2.8

Table S8. Inhibition of human liver microsomal P450s. Inhibition constants (IC_{50} values, μM) of CYP125/142 inhibitors **5d**, **5k**, and **5m** were determined for select human P450 isoforms using the following substrates: CYP1A-ethoxyresorufin, CYP2C19- mephenytoin, CYP2C9-tolbutamide, CYP2D6-dextromethorphan, CYP3A4-midazolam/testosterone.

Compound	**CYP1A**	**CYP2C19**	**CYP2C9**	**CYP2D6**	**CYP3A4**
**5d**	0.11	<0.10	0.14	0.46	0.52/1.49
**5k**	5.95	3.1	5.27	0.18	0.26/1.25
**5m**	0.82	0.44	1.86	0.33	0.52/1.82

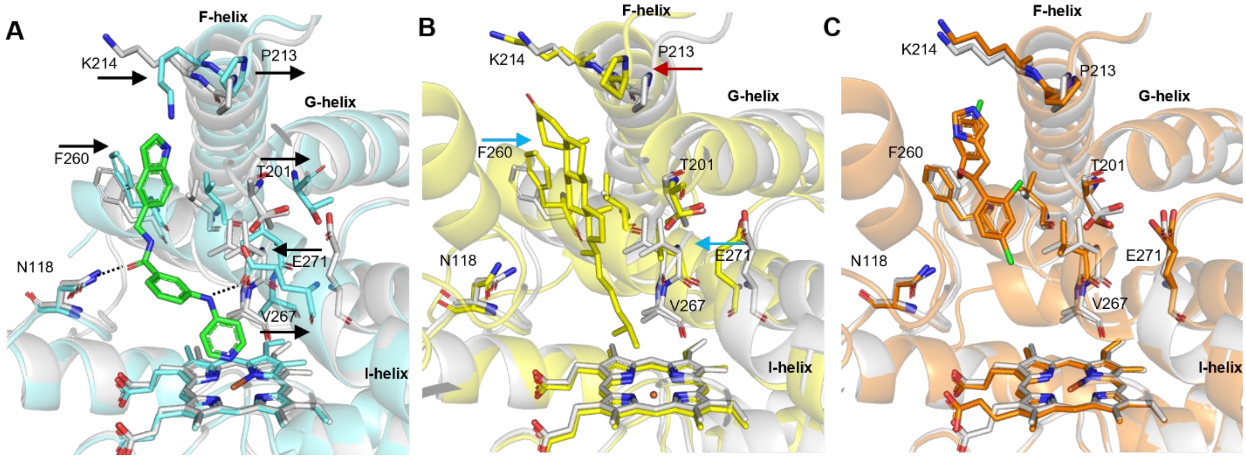

Figure S1. Compound **5g** induces a unique conformation in the CYP125 active site. Aligned X-ray crystal structures of apoenzyme CYP125 (grey, PDB 3IW0) and (A) **5g**-CYP125 (green ligand, blue cartoon, PDB 8S4M), (B) cholestenone (yellow, PDB 2X5W), or (C) econazole-CYP125 (orange, PDB 3IW2). Key residues are annotated, with arrows indicating the shift in orientation relative to apo-CYP125. Blue/red arrows in (B) indicate similar/opposite orientation to **5g,** respectively. Predicted hydrogen bonds between **5g** and N118 or E271 are shown as black dashed lines. RMSD of 8S4M-3IW0 = 0.694; 8S4M-2X5W = 1.108; 8S4M-3IW2 = 0.703.

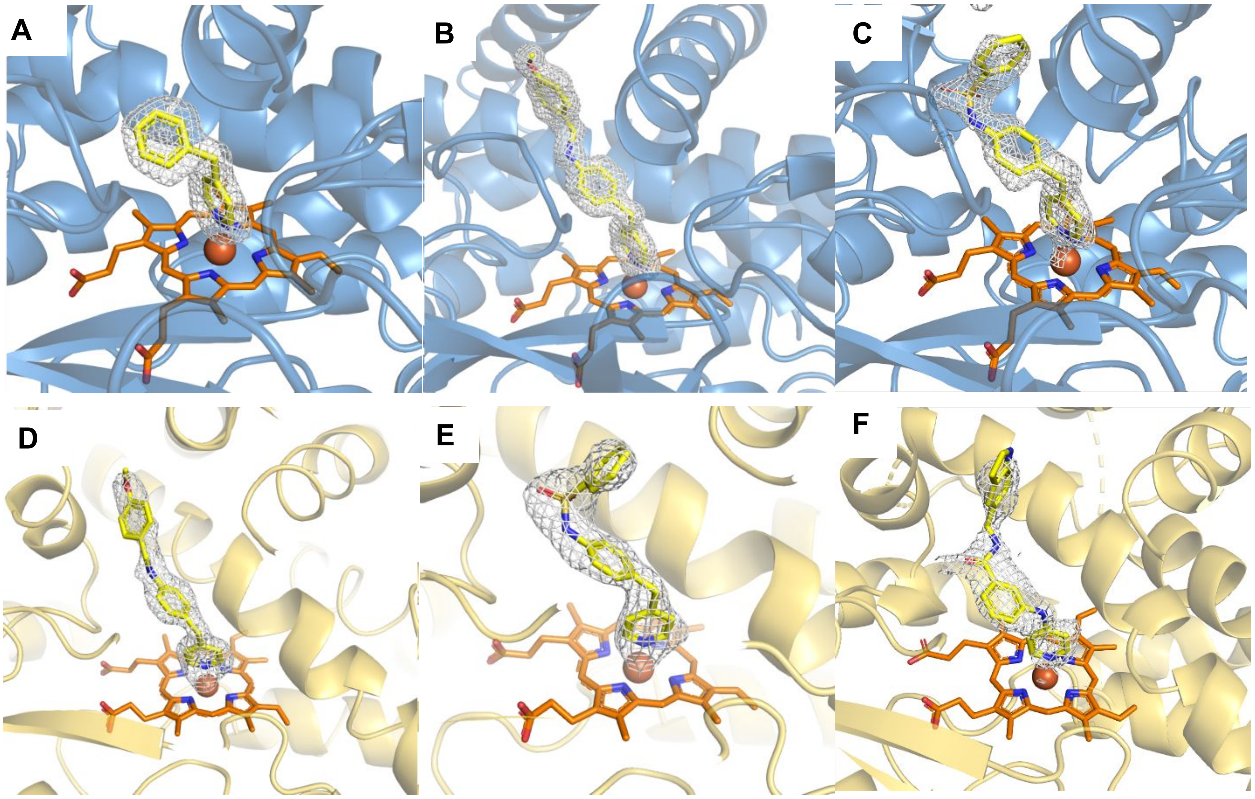

Figure S2. Electron density maps for ligands shown in Figures 1a and 3a-d. Polder maps (grey mesh) are contoured to 3σ, ligands are shown as yellow sticks, in complex with either CYP125 (yellow cartoon), or CYP142 (blue cartoon). (A) CYP142-**1a-i** (PDB 8S53); (B) CYP142-**5m**; (PBD 7P5T); (C) CYP142-**5j** (PBD 7QQ7); (D) CYP125-**5m** (PDB 7ZIC); (E) CYP125-**5j** (PDB 7ZGL); and (F) CYP125-**5g** (PDB 8S4M).

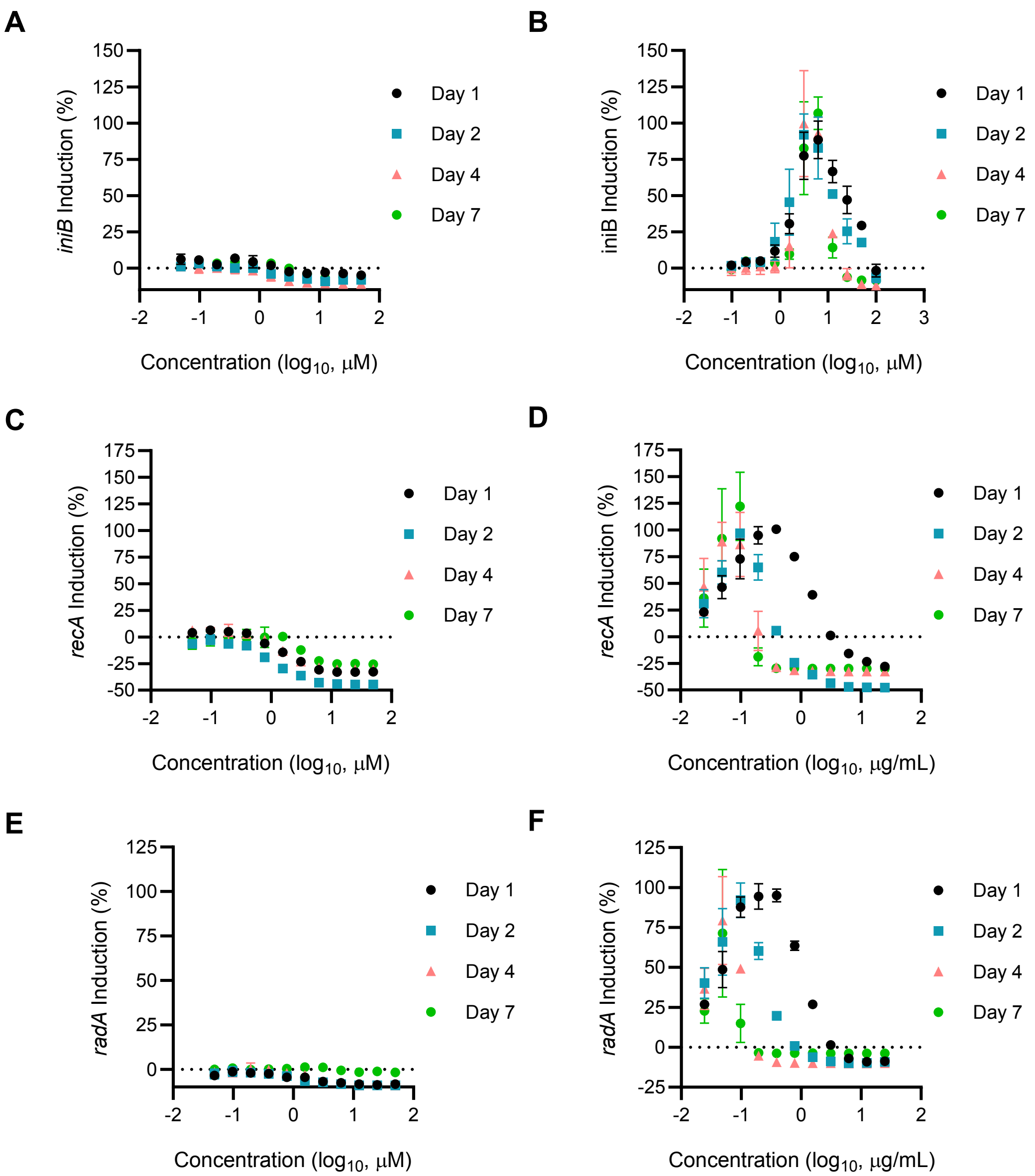

Figure S3. Transcriptional reporter assays to determine **5m** mechanisms of action. Induction of bioluminescent reporter for cell wall damage response (*iniB*) or DNA damage response (*recA* or *radA)* after *Mtb* were treated with compound 5m (A, C, E) or positive control compound SQ109 (B) or moxifloxacin (D, F). Signal intensity of the reporter was adjusted as a %max signal induced by positive control, and data are plotted as mean values ± SD of n=2 replicates.

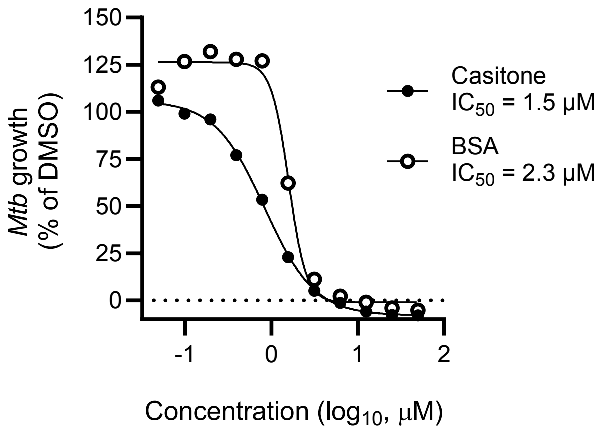

Figure S4. Non-specific protein binding reduces extracellular antitubercular activity of CYP125-142 inhibitors. Dose-response curves showing the inhibition of H37Rv *Mtb* growth by compound **5m** when bacteria are cultured on cholesterol media supplemented with either casitone or bovine serum albumin (BSA). Bacterial growth quantified by ATP-luminescence 2-weeks post-compound treatment and are reported as a percent of the DMSO-treated control.

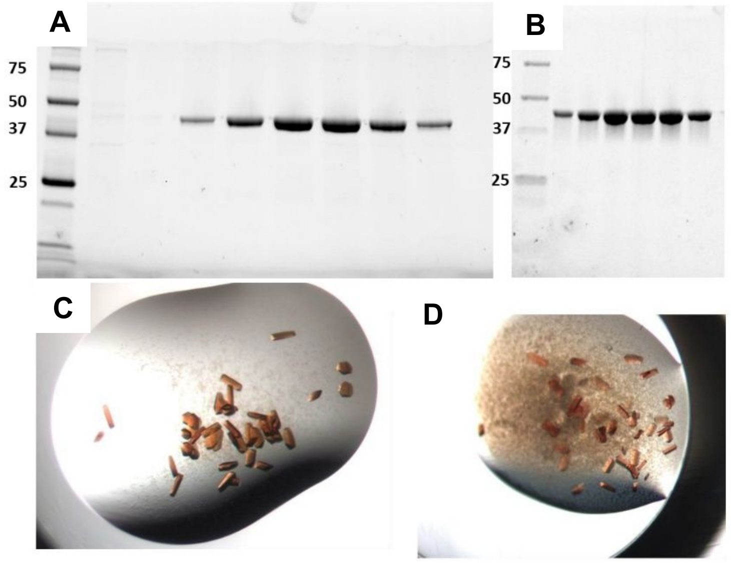

Figure S5. Purification and crystallization of recombinant CYP125 and CYP142. SDS-PAGE gels of purified recombinant (a) CYP125 and (b) CYP142 proteins, and crystals used to obtain structures of lead compounds **5m** and **5j** with (c) CYP125 and (d) CYP142. Gels are representative of protein purity used throughout the study, and have been previously reported (Snee, M. PhD Thesis, University of Manchester, (2023)).

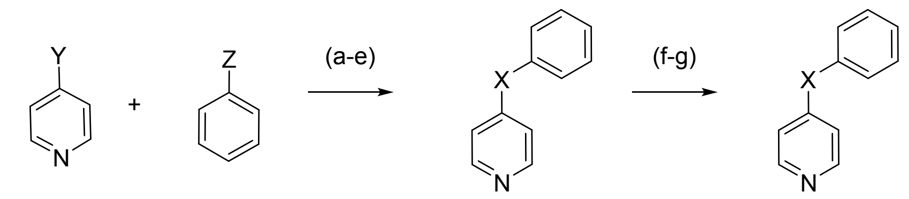

Y = Cl, I, Br, CH_3 | Z = NH_2, NHPh, OH, SO_3Na, COH

2a X = NH (a, 53%)
2c X = NPh (b, 6%)
2d X = O (c, 4%)
2e X = SO_2 (d, 2%)
2g X = *E*-CH=CH (e, 39%)

2b X = NMe (f, 75%)
2h X = CH_2CH_2 (g, 94%)

Scheme S1. Synthesis of **1a-i** analogues to explore SAR of benzylic position. *Reagents and conditions:* (a) Y=Cl.HCl, Z=NH_2, HCl (37%), EtOH, 90 °C, 20 h; (b) Y=Br.HCl, Z=NHPh, K^{t}OBu, $Pd(OAc)_2$, *rac*-BINAP, toluene, 70 °C, 16 h; (c) Y=Cl.HCl, Z=OH, Cu(s) powder, Cs_2CO_3, DMF, 100 °C, 18 h; (d) Y=I, Z=SO_3Na, *L*-proline sodium salt, CuI, DMSO, 80 °C, 44 h; (e) Y=CH_3, Z=COH, Ac_2O, 140 °C, 24 h; (f) **2a**, MeI, K^{t}OBu, DMF, r.t. 19 h; (g) **2g**, H_2(g), Pd/C, EtOH, r.t., 20 h.

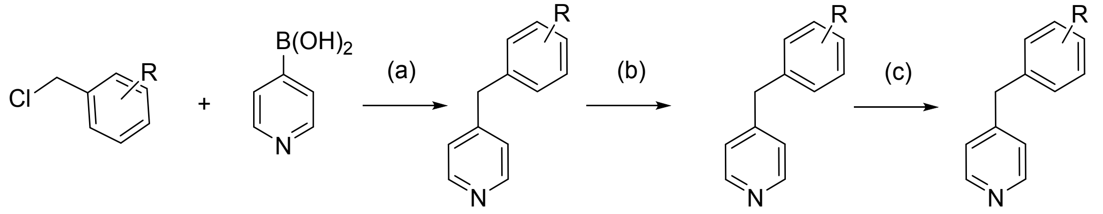

R = 3-OMe, 4-OMe, 3-NO_2

3a R = 3-OMe (58%)
3b R = 4-OMe (25%)
3c R = 3-NO_2 (88%)

3d R = 3-NH_2 (71%)
3e R = 4-NH_2 (NA)

3f R = 4-NHCOMe (c, 85%)
3g R = 4-SO_2Me (d, 64%)

Scheme S2. Synthesis of **1a-i** analogues to explore SAR of "linker". *Reagents and conditions:* (a) $Pd(PPh_3)_4$, Na_2CO_3, DME:H_2O (2:1), 100 °C, 4 h; (b) **3c**, Pd/C, N_2H_4.xH_2O, EtOH, 90 °C, 2 h; (c) **3f** (d) **3g**. N/A – obtained from commercial sources.

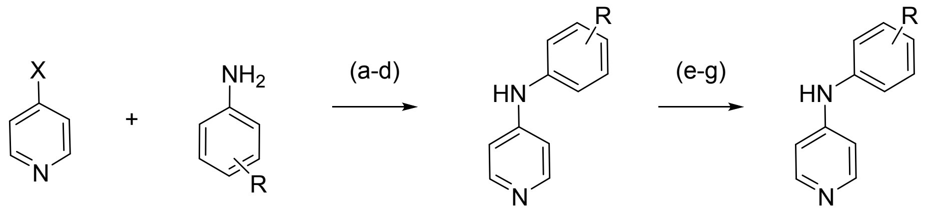

X = Cl, or NH_2*

R = 3/4-NO_2, 3/4-CO_2Me, 1-Br-3-I-benzene*, or 4-Br

6a R = 3-NO_2 (a, 64%)
6b R = 4-NO_2 (a, 55%)
4c R = 3-CO_2CH_3 (a, 43%#)
4d R = 4-CO_2CH_3 (b, 43%)
4h R = 3-Br (c, 57%)*
4i R = 4-Br (d, 10%)

4a R = 3-NH_2 (e, 86%)
4b R = 4-NH_2 (e, 95%)
4e R = 3-CO_2H (f, 49%)
4f R = 4-CO_2H (g, 39%)
4g R = 3-CH_2OH (h, 92%)

Scheme S3. Synthesis of **2a** analogues to explore SAR of "*linker*"*:* (a) X = Cl.xHCl, HCl (37%), EtOH, 90 °C, 20 h; (b) X = Cl.xHCl, R=4-CO_2Me, AcOH, LiOH.H_2O, MeOH:H_2O:THF, r.t., 4 h; (c) X = NH_2, 1-bromo-3-iodobenzene, $Pd_2(dba)_3$, DPPF, Na^{t}OBu, toluene, 115 °C, 24 h; (d) $Pd_2(dba)_3$, IPr.HCl, K^{t}OBu, 1,4-dioxane, 100 °C, 21 h; (e) **6a/b**, $SnCl_2$.2H_2O, HCl (37%), EtOH, 0-80 °C, 1−3 h; (f) **4c**, LiOH.H_2O, MeOH:H_2O:THF, r.t., 4 h; (g) **4d**, KOH(aq), EtOH, reflux, 2 h; (h) **4c**, $LiAlH_4$, THF, 0 °C-r.t., 20 h. #impure mixture of methyl/ethyl ester.

NMR spectra of final compounds tested in anti-tubercular assays

N-(4-(Pyridin-4-ylmethyl)phenyl)benzamide, (5a), 1H-NMR, 400 MHz, d6-DMSO

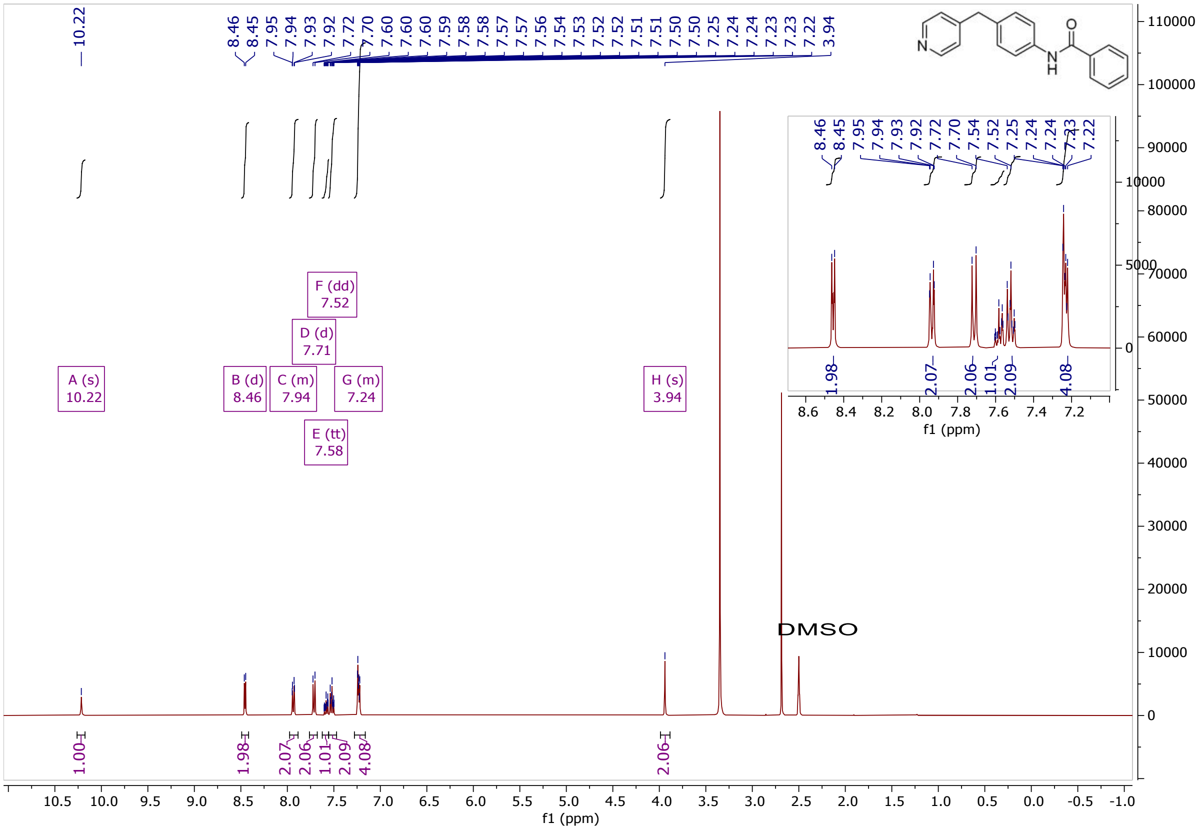

N-(4-(Pyridin-4-ylmethyl)phenyl)benzamide, (5a), 13C-NMR, 100 MHz, d6-DMSO

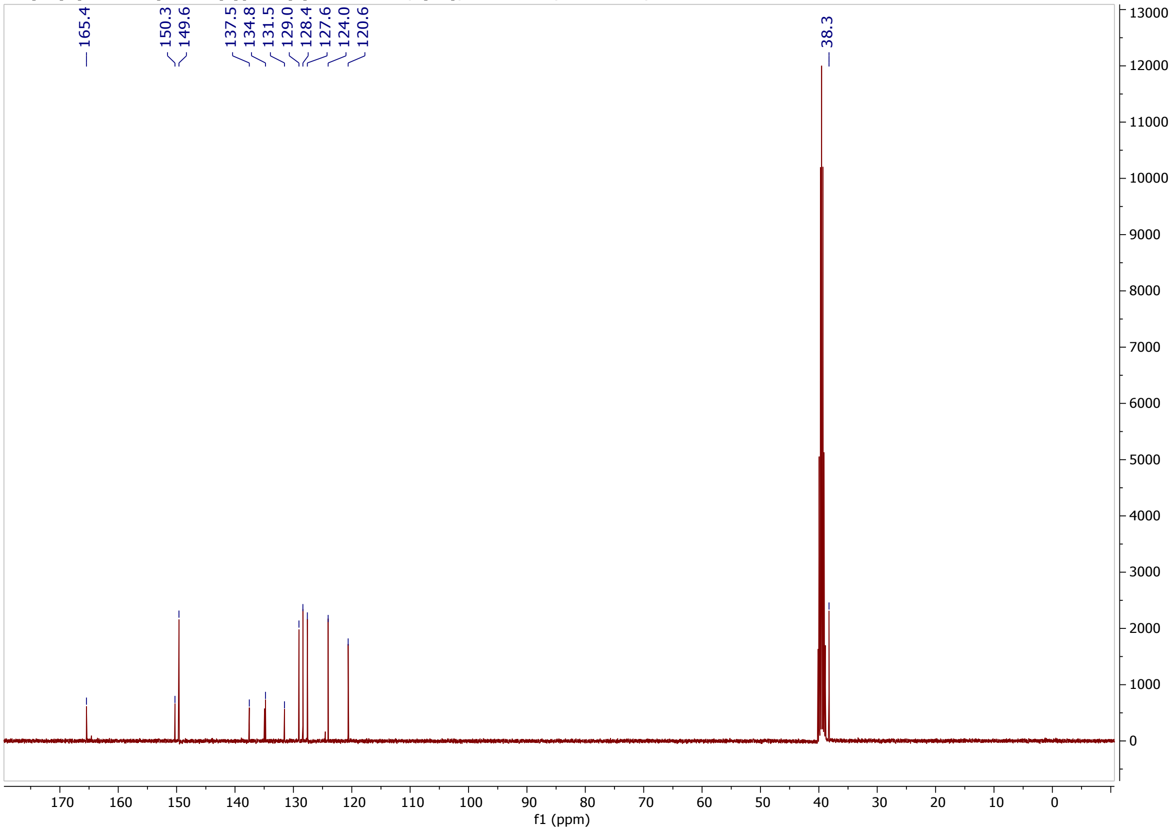

N-(4-(Pyridin-4-ylamino)phenyl)benzamide, (5b), 1H-NMR, 500 MHz, d6-DMSO

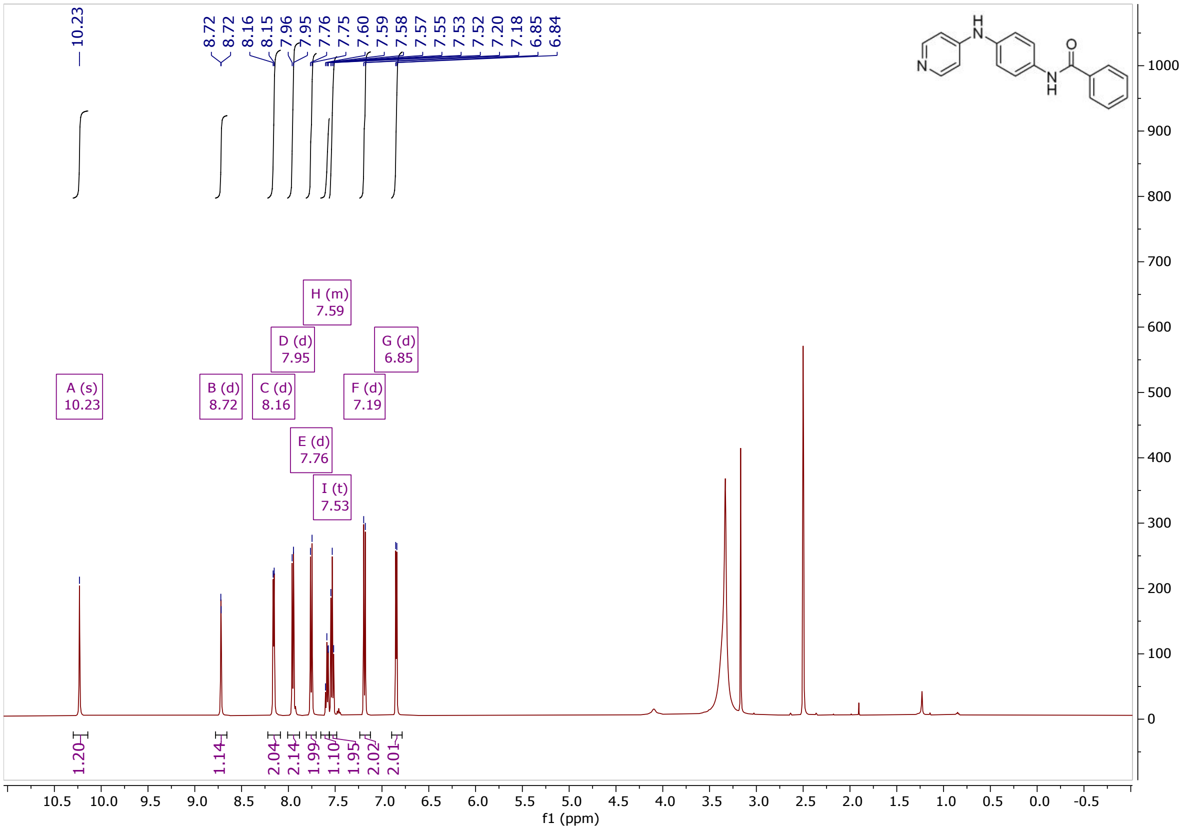

N-(4-(Pyridin-4-ylamino)phenyl)benzamide, (5b), 13C-NMR, 125 MHz, d6-DMSO)

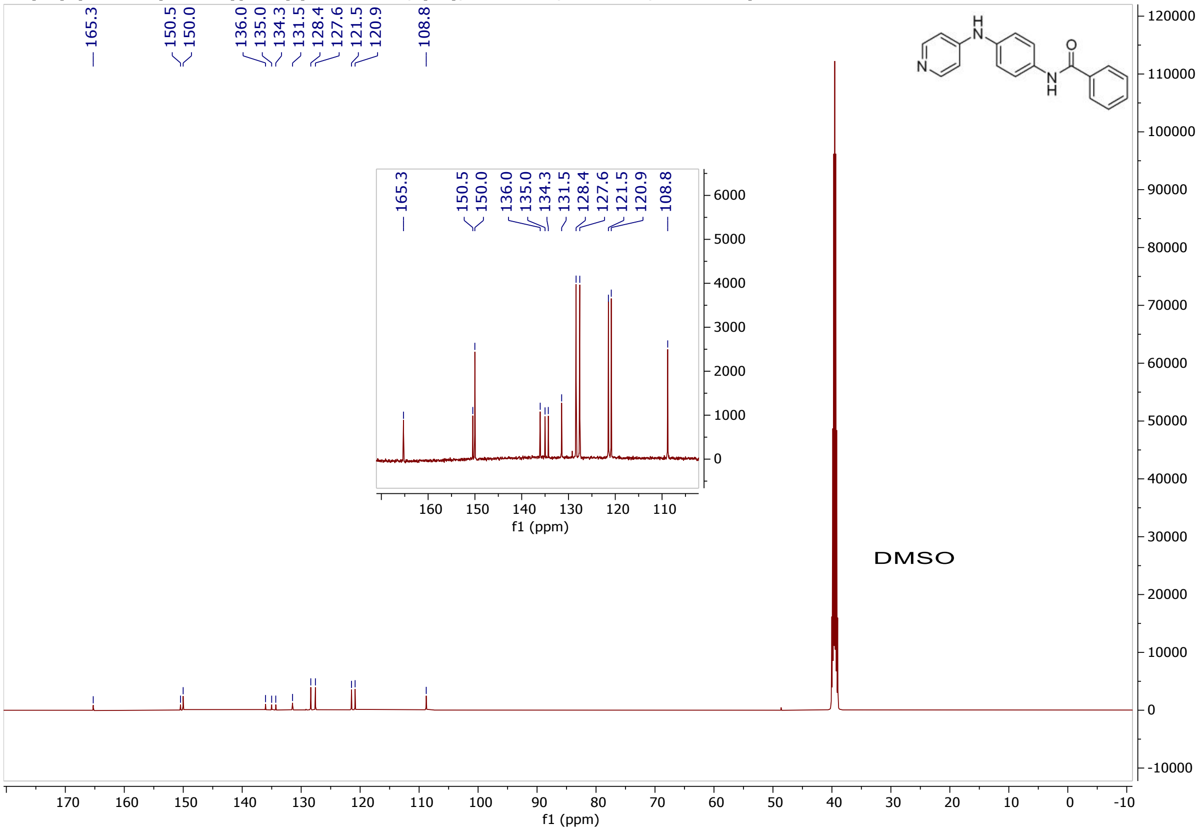

N-(4-(Methyl(pyridin-4-yl)amino)phenyl)benzamide, (5c), 1H-NMR, 400 MHz, CDCl3

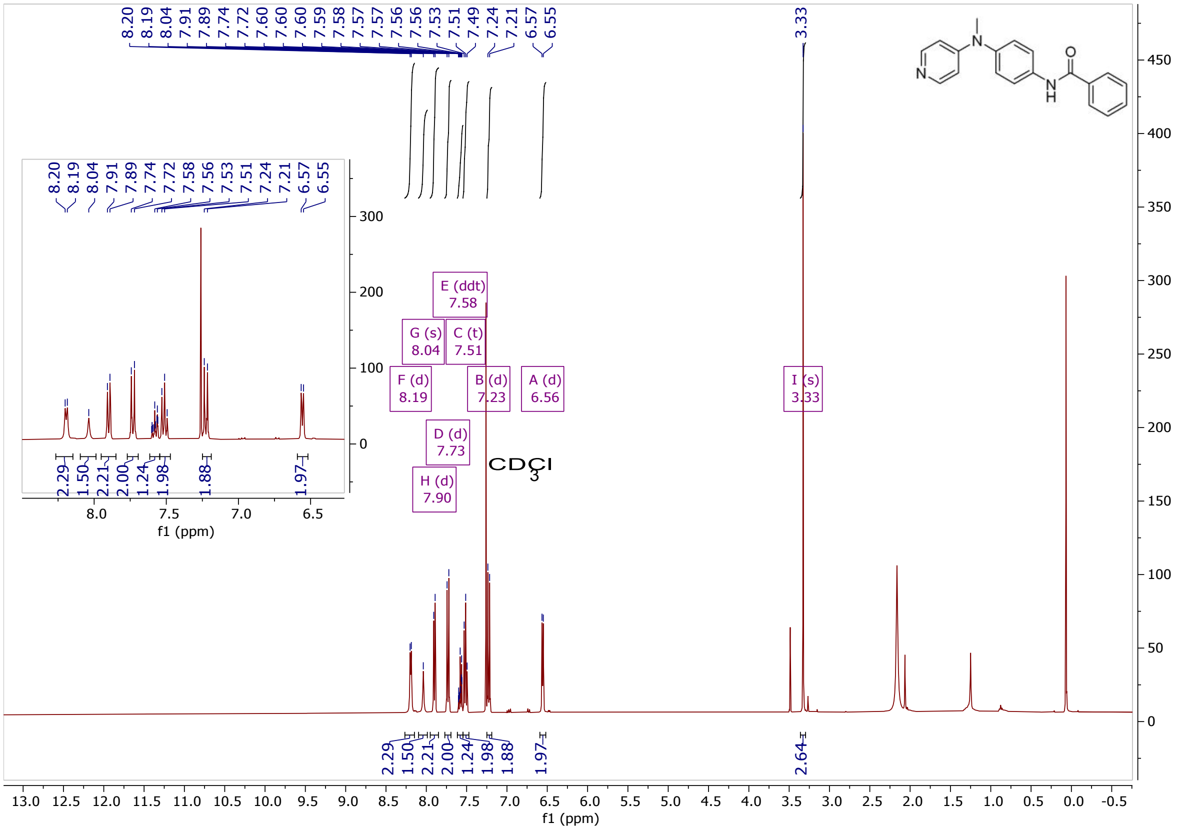

N-(4-(Methyl(pyridin-4-yl)amino)phenyl)benzamide, (5c), 13C-NMR, 100 MHz, CDCl3

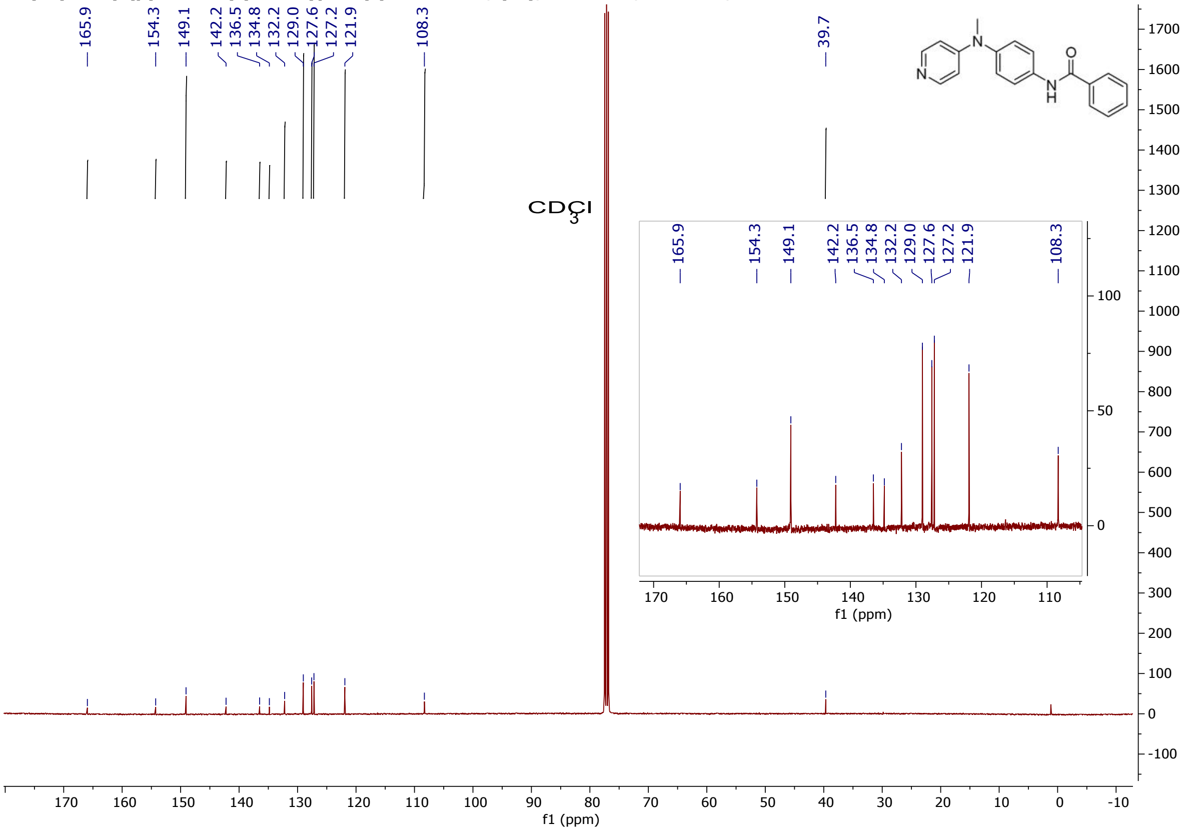

(S)-2-(Benzylamino)-3-(1H-indol-3-yl)-N-(4-(pyridin-4-ylmethyl)phenyl)propanamide, (5d), 1H-NMR, 500 MHz, CDCl3

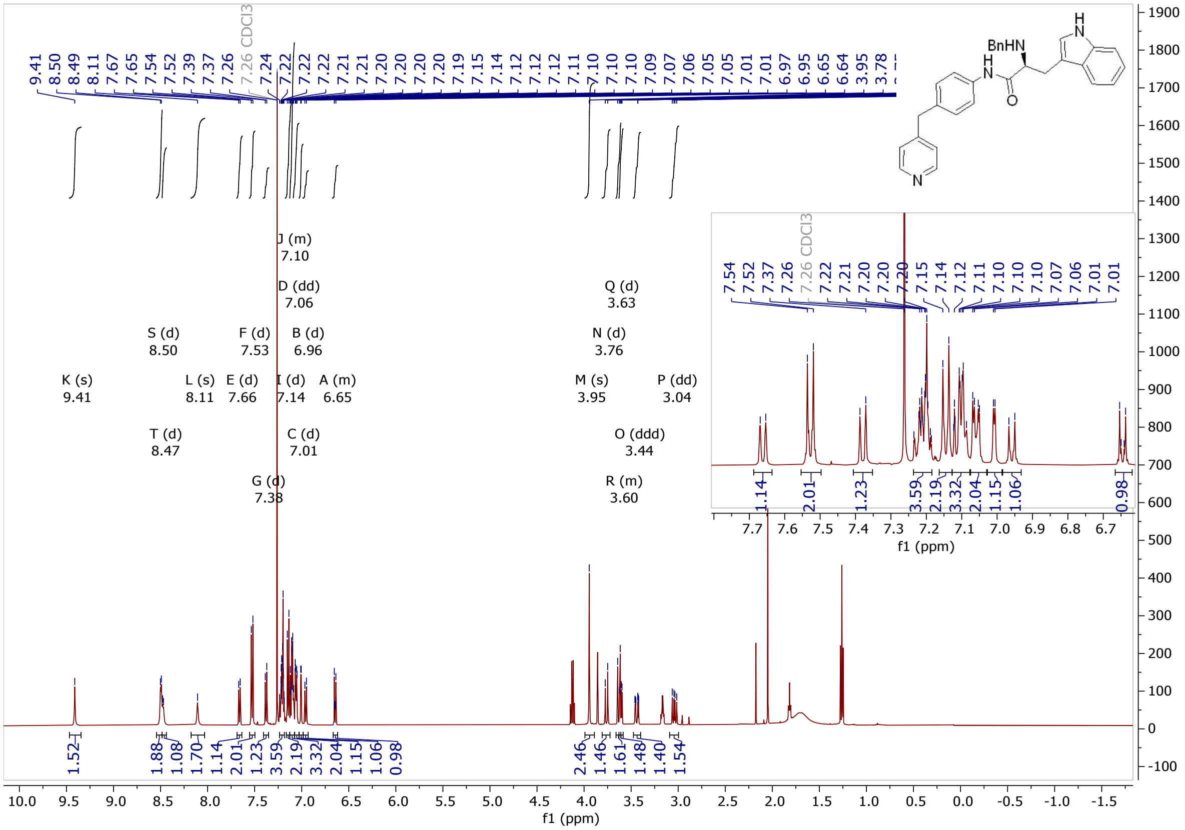

(S)-2-(Benzylamino)-3-(1H-indol-3-yl)-N-(4-(pyridin-4-ylmethyl)phenyl)propanamide, (5d), 13C-NMR, 125 MHz, CDCl3

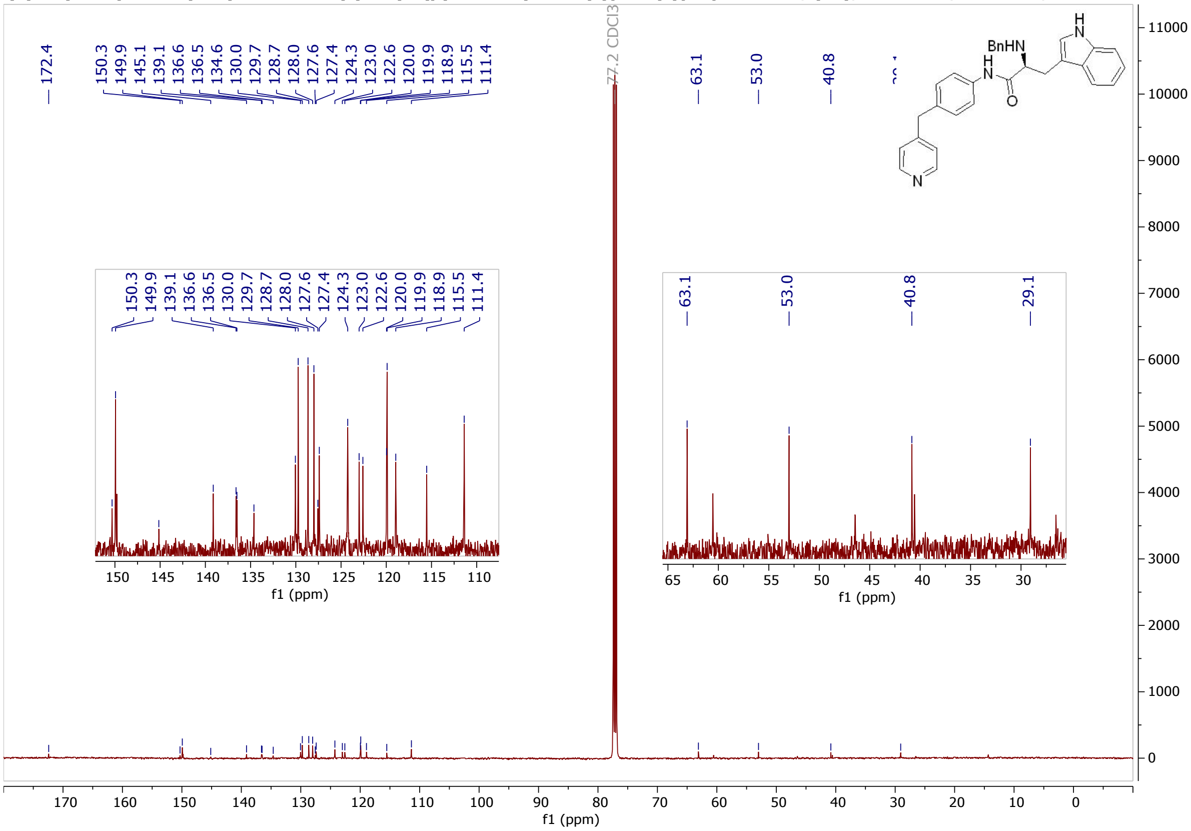

N-((1H-Indol-5-yl)methyl)-3-(pyridin-4-ylmethyl)benzamide, (5e), 1H-NMR, 500 MHz, CDCl3

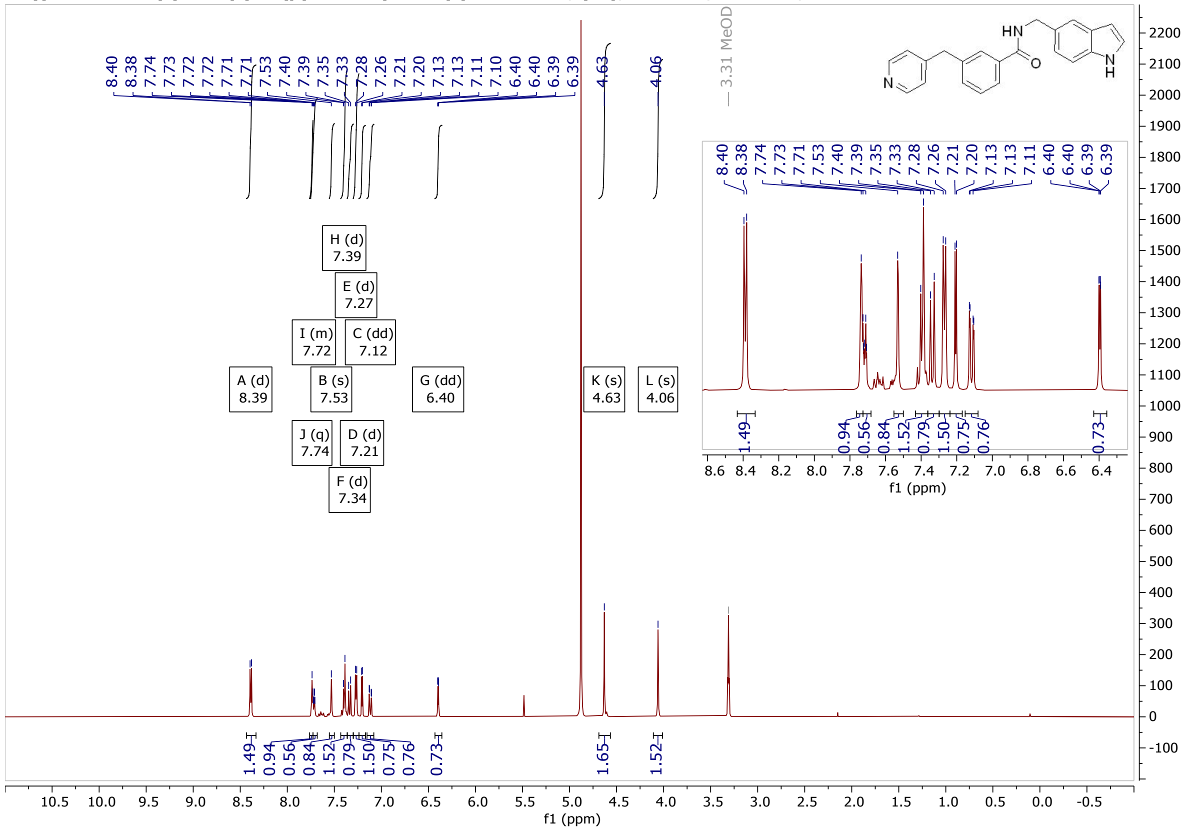

N-((1H-Indol-5-yl)methyl)-3-(pyridin-4-ylmethyl)benzamide, (5e), 13C-NMR, 125 MHz, CDCl3

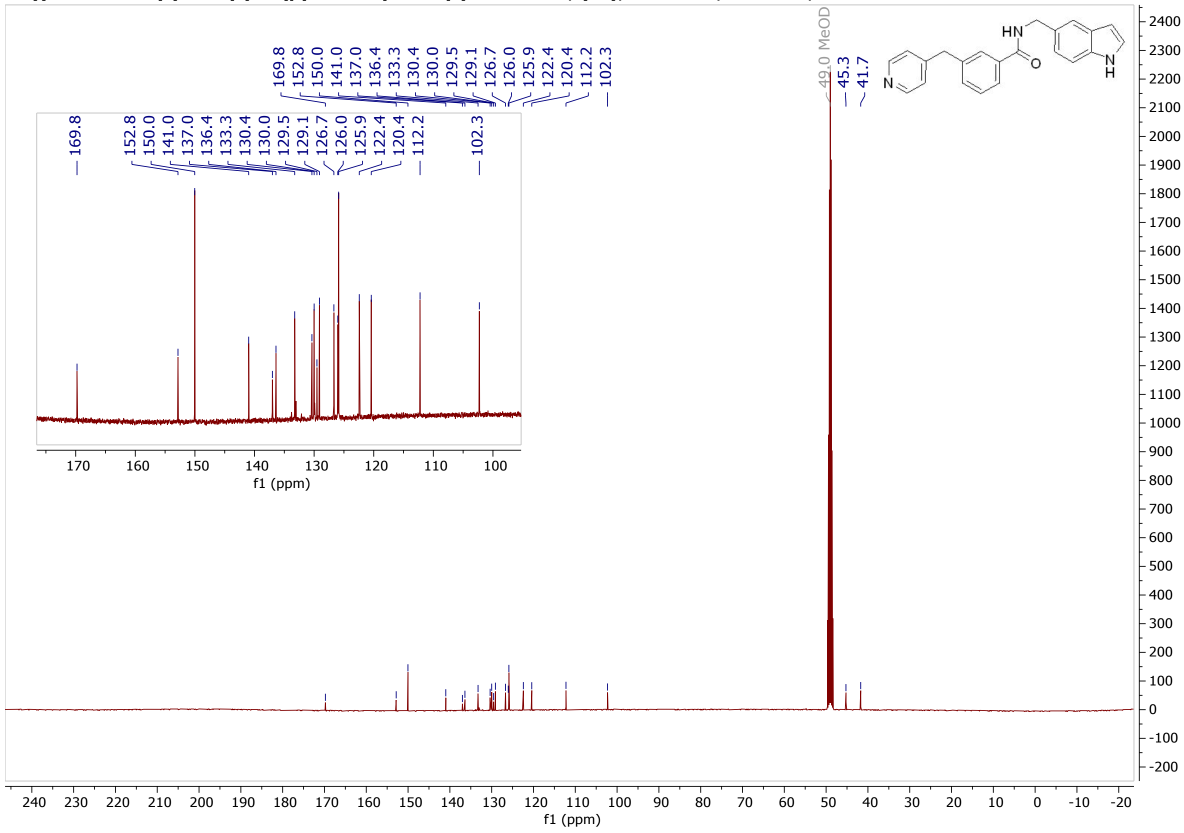

N-((1H-Indol-5-yl)methyl)-4-(pyridin-4-ylmethyl)benzamide, (5f), 1H-NMR, 500 MHz, MeOD

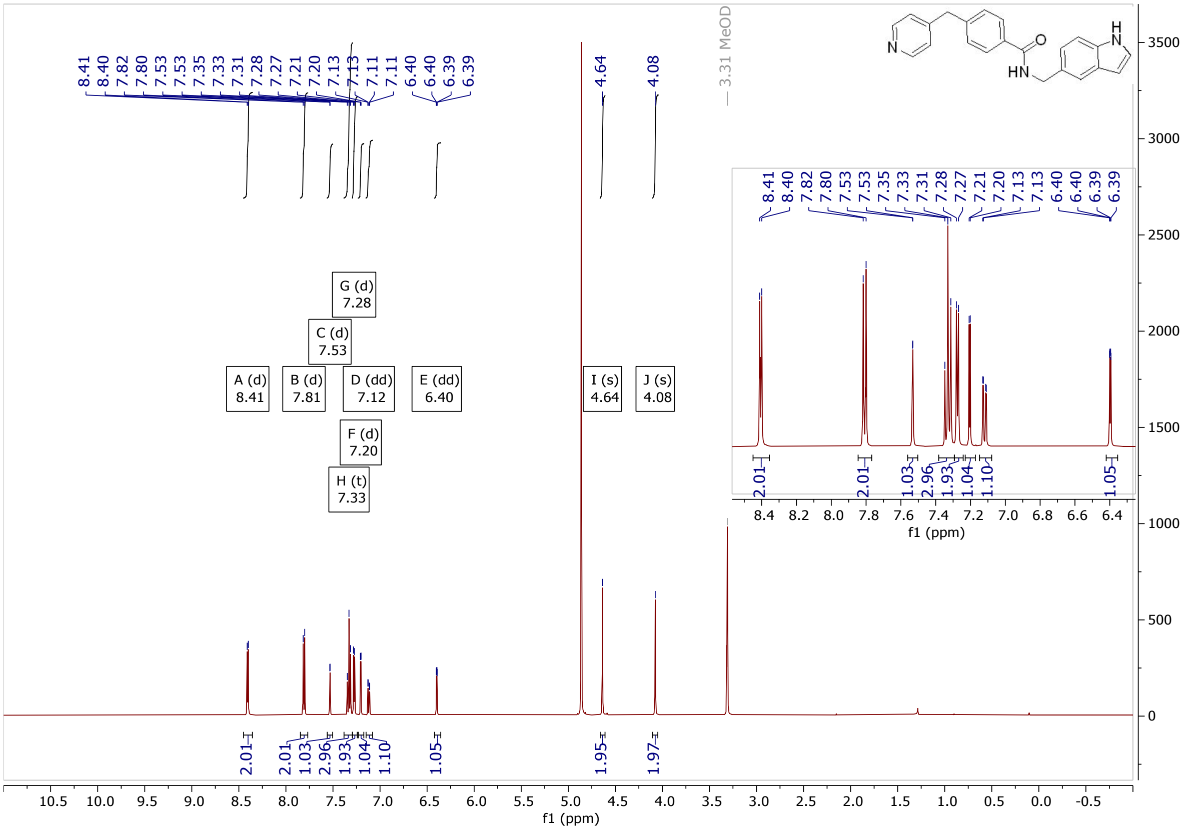

N-((1H-Indol-5-yl)methyl)-4-(pyridin-4-ylmethyl)benzamide, (5f), 13C-NMR, 125 MHz, MeOD

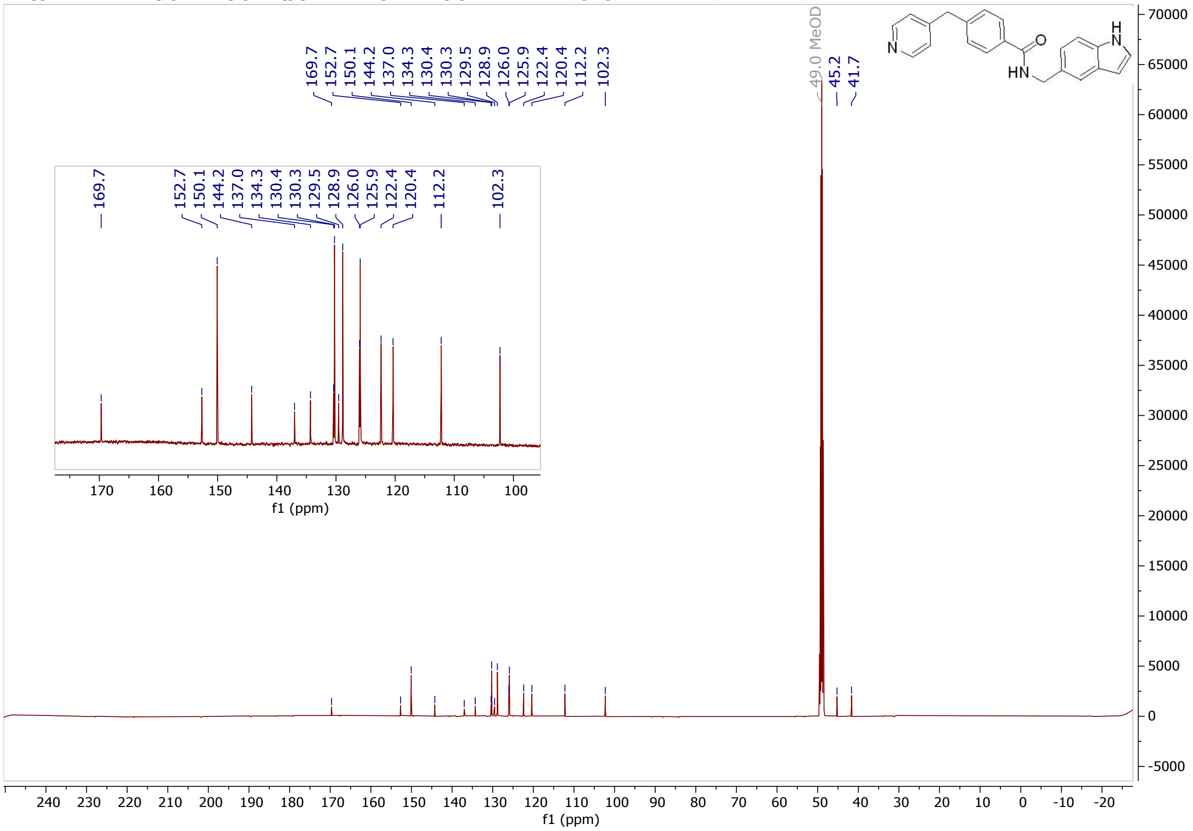

(R)-N-(1-Phenylethyl)-3-(pyridin-4-ylmethyl)benzamide, (5h), 500 MHz, MeOD

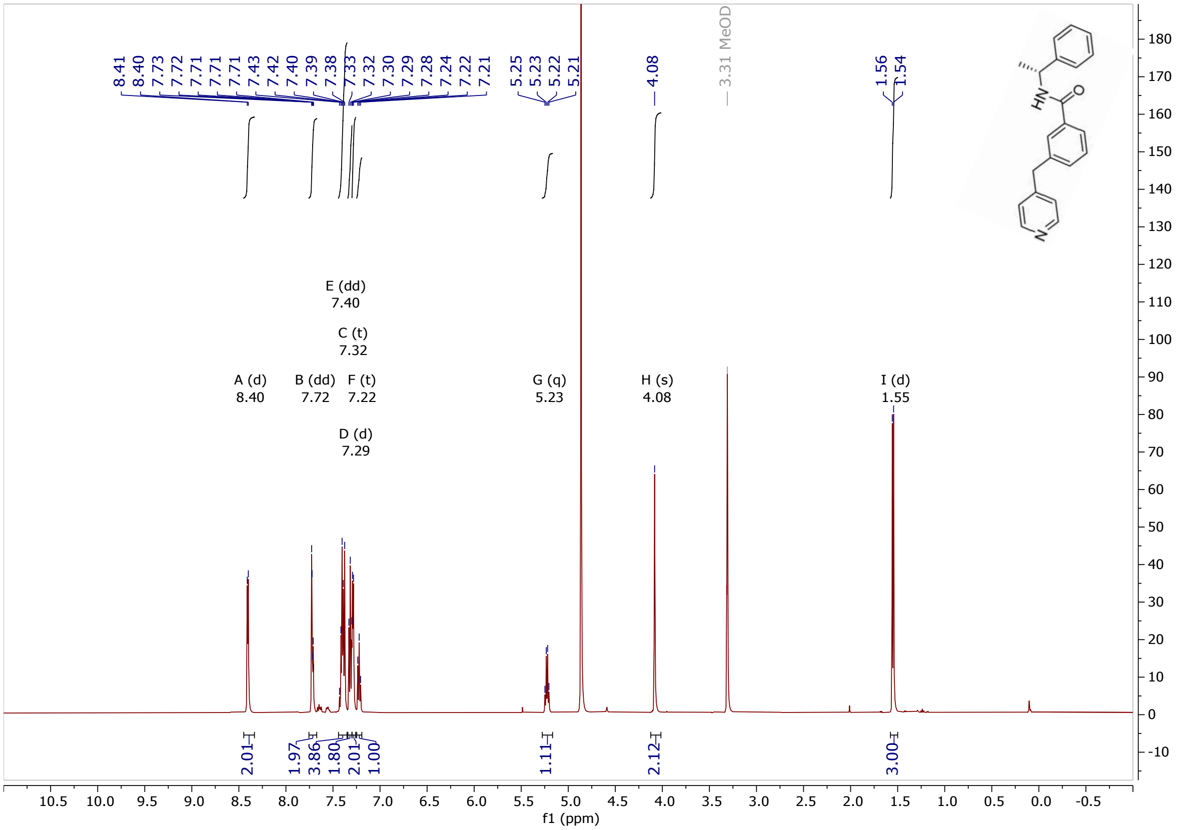

(R)-N-(1-Phenylethyl)-3-(pyridin-4-ylmethyl)benzamide, (5h), 125 MHz, MeOD

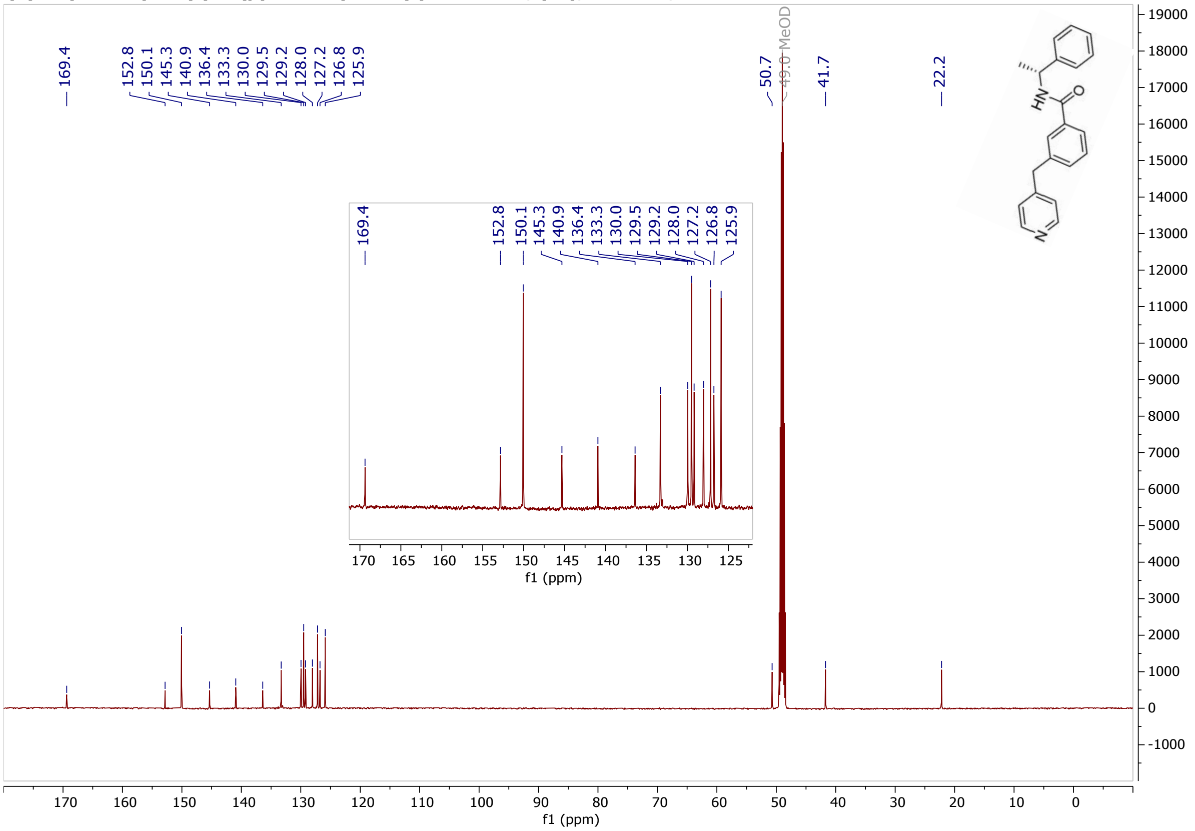

(R)-N-(1-Phenylethyl)-4-(pyridin-4-ylmethyl)benzamide, (5i), 500 MHz, MeOD

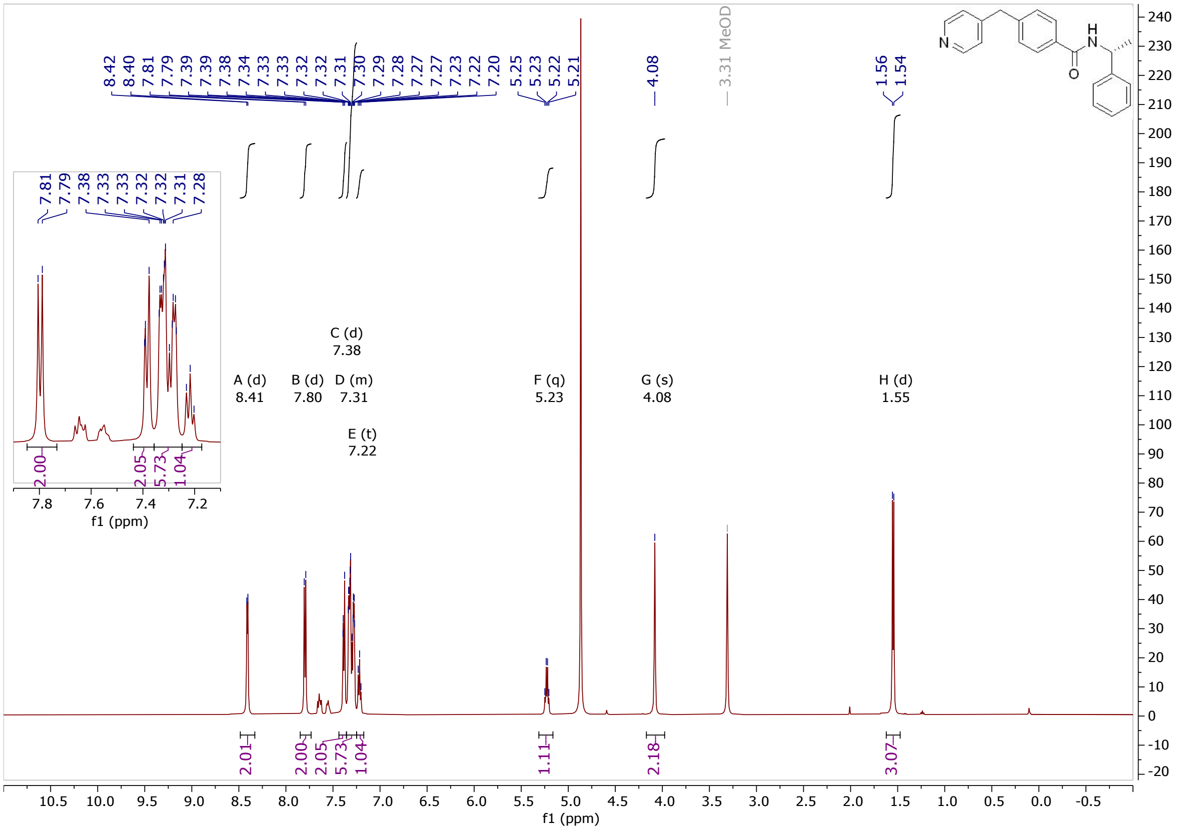

(R)-N-(1-Phenylethyl)-4-(pyridin-4-ylmethyl)benzamide, (5i), 500 MHz, MeOD

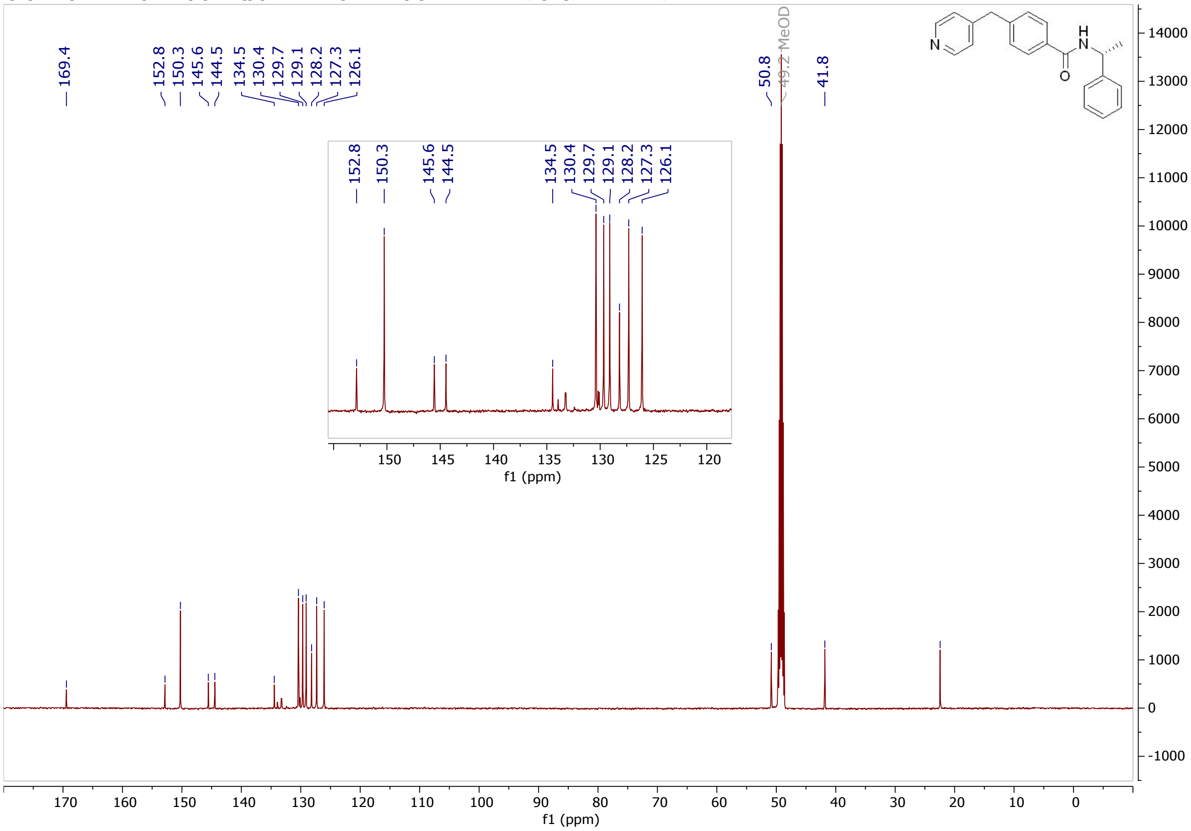

N-(4-(Pyridin-4-ylmethyl)phenyl)benzenesulfonamide, (5j), 1H-NMR, 400 MHz, d6-DMSO

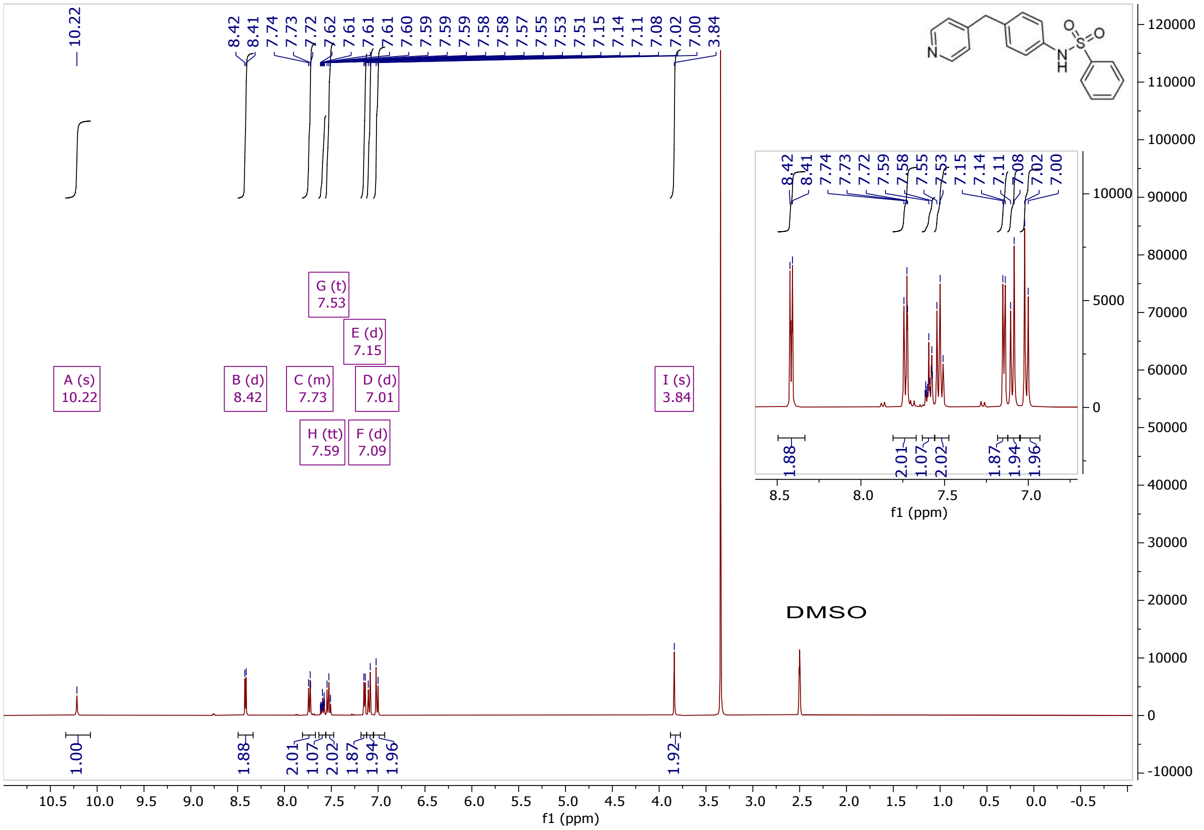

N-(4-(Pyridin-4-ylmethyl)phenyl)benzenesulfonamide, (5j), 13C-NMR, 100 MHz, d6-DMSO

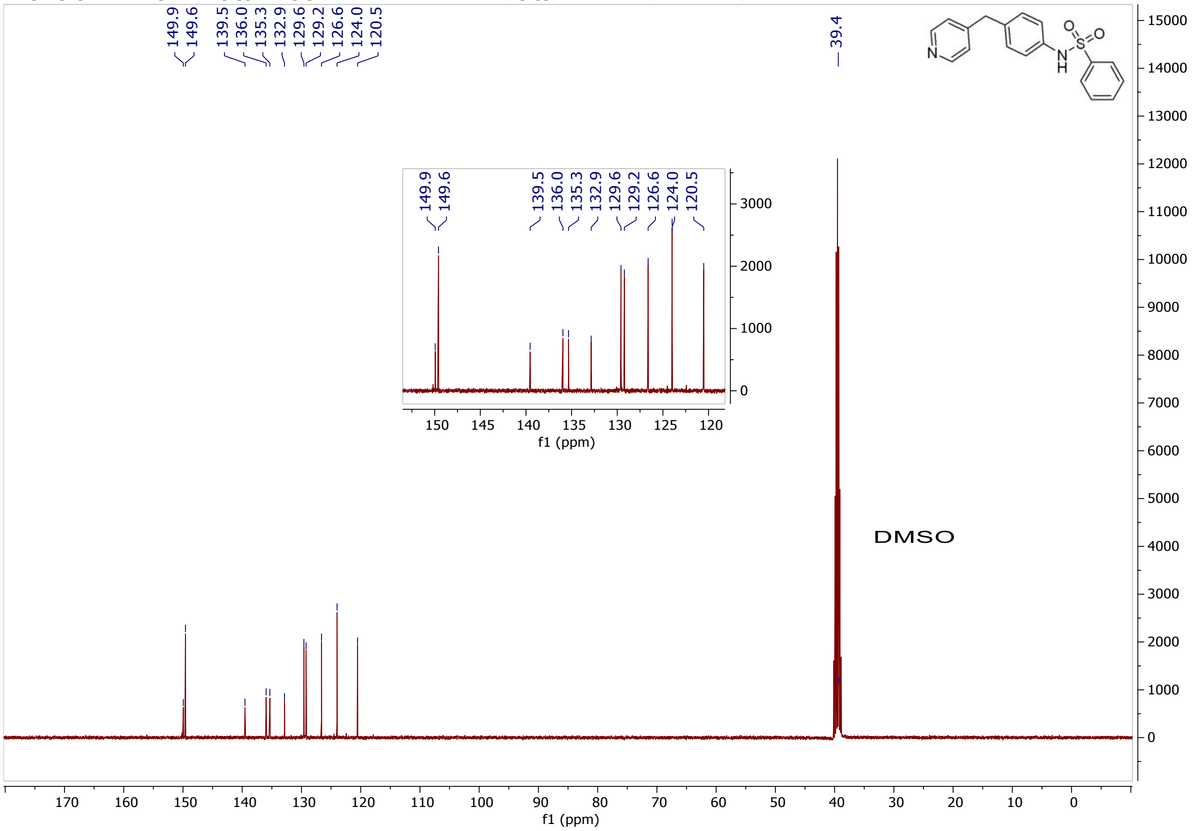

N-(4-(Pyridin-4-ylmethyl)phenyl)-4-(trifluoromethoxy)benzenesulfonamide, (5k), 1H-NMR, CDCl3

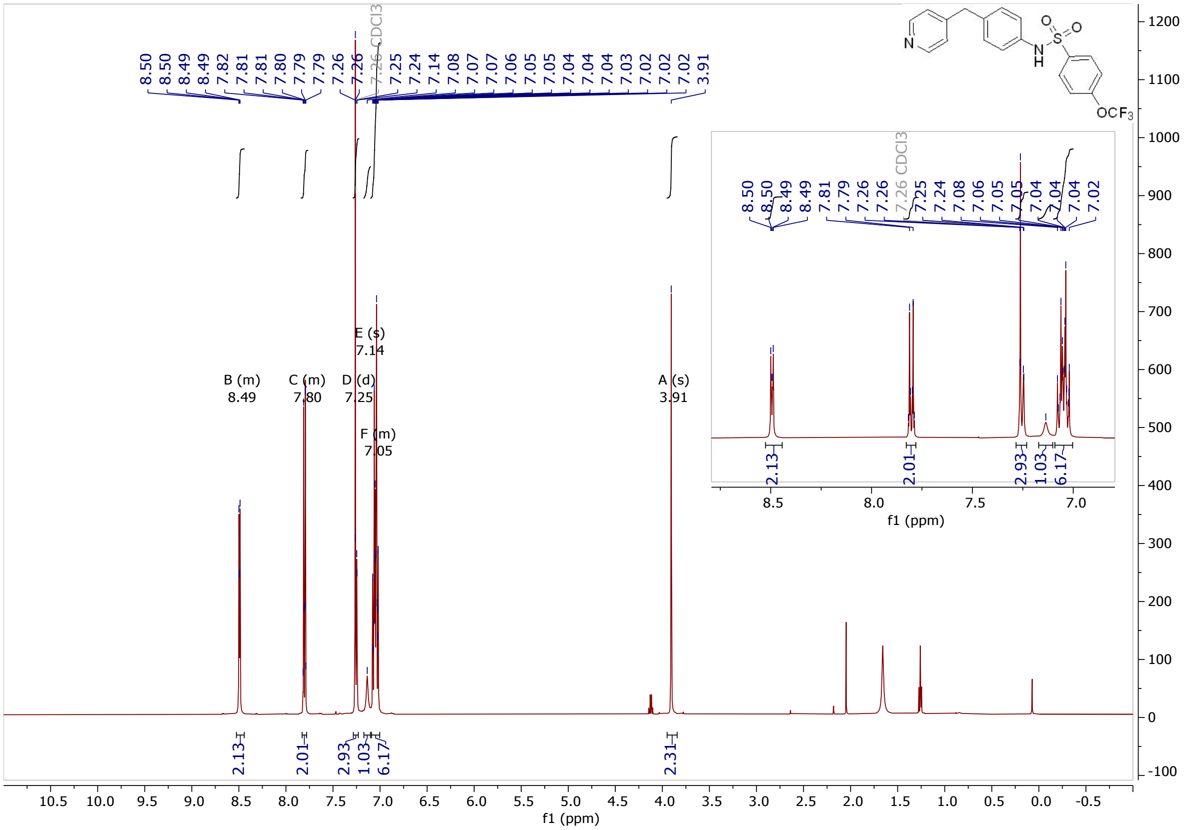

N-(4-(Pyridin-4-ylmethyl)phenyl)-4-(trifluoromethoxy)benzenesulfonamide, (5k), 13C-NMR, CDCl3

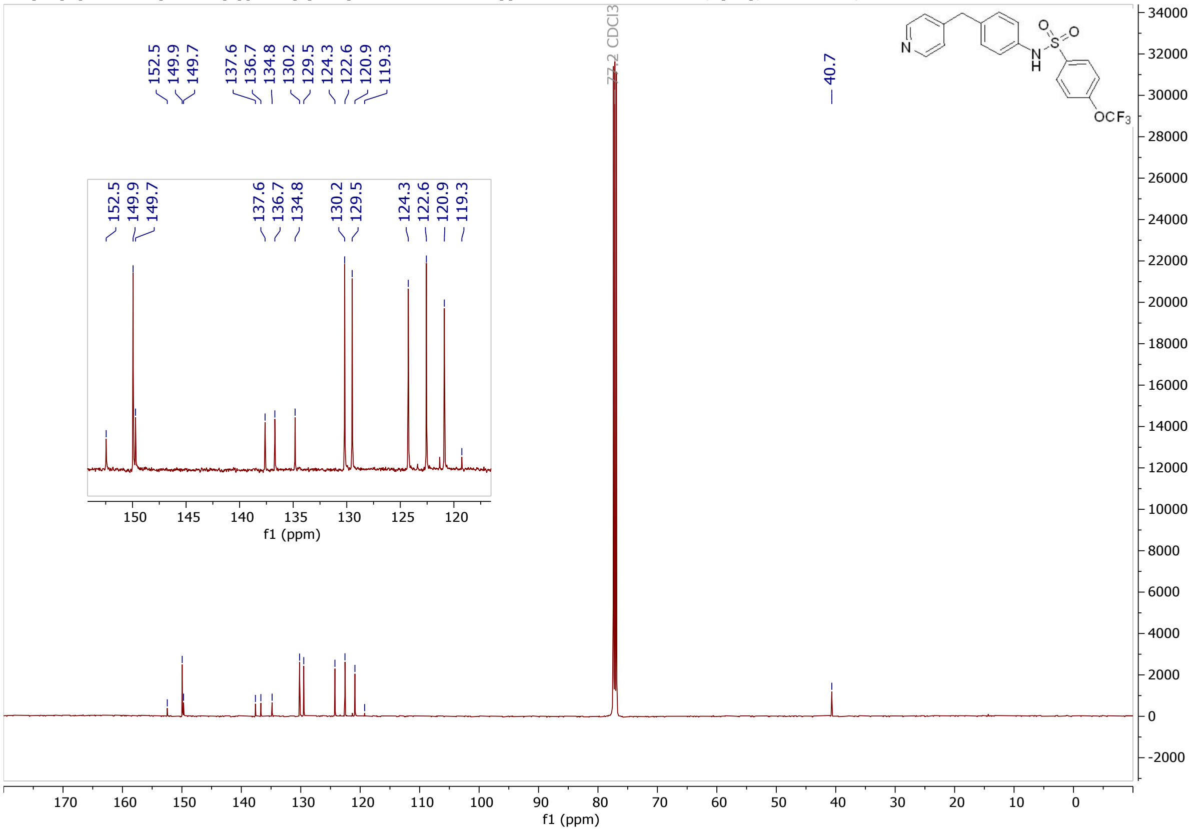

N-(4-Methoxybenzyl)-3-(pyridin-4-ylmethyl)aniline, (5l), 1H-NMR, 500 MHz, CDCl3

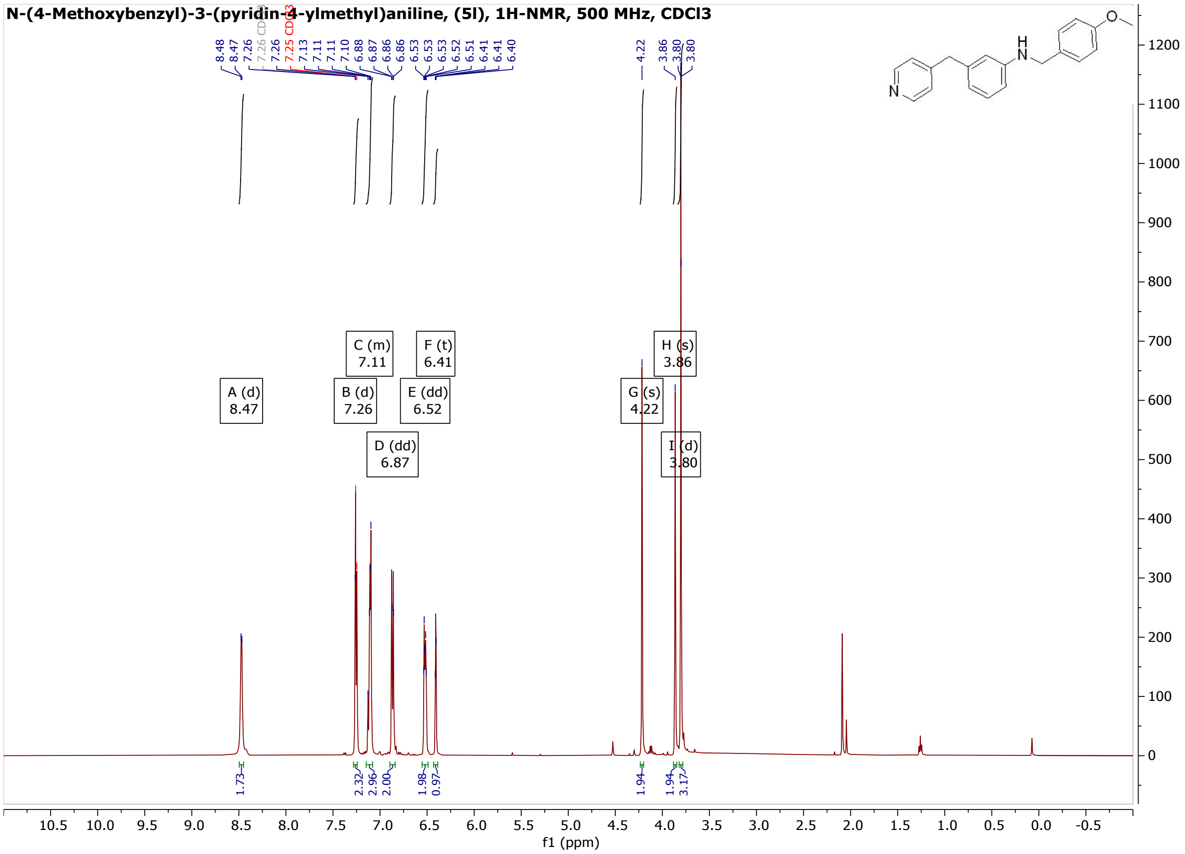

N-(4-Methoxybenzyl)-3-(pyridin-4-ylmethyl)aniline, (5l), 1H-NMR, 500 MHz, CDCl3

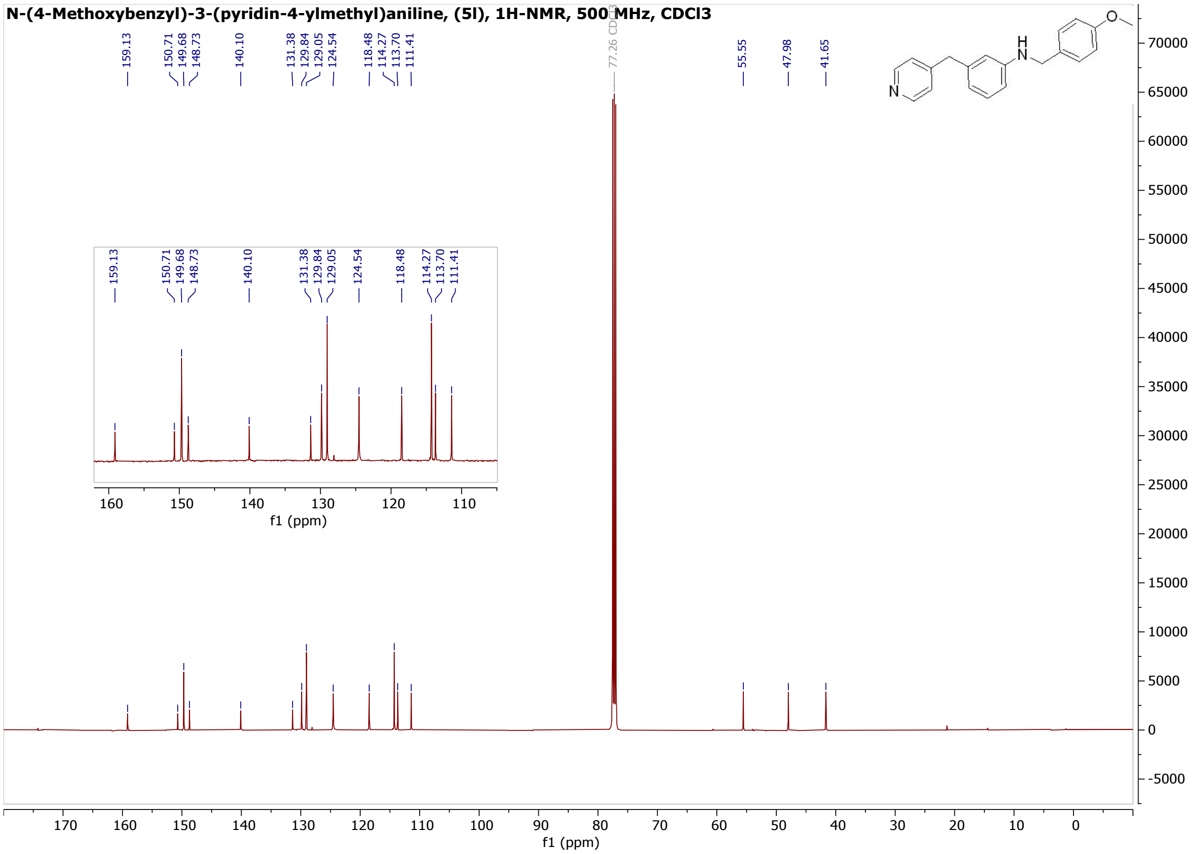

N-(4-Methoxybenzyl)-4-(pyridin-4-ylmethyl)aniline, (5m), 1H-NMR, 500 MHz, CDCl3

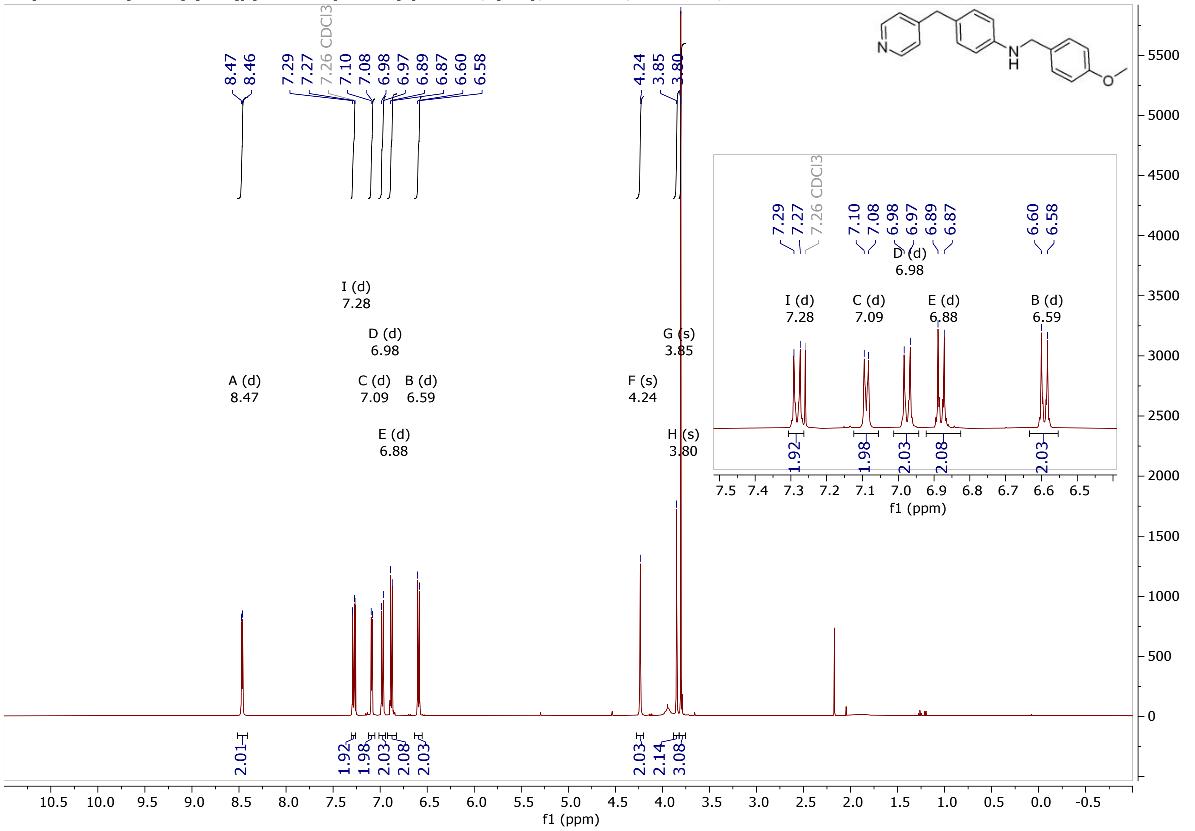

N-(4-Methoxybenzyl)-4-(pyridin-4-ylmethyl)aniline, (5m), 13C-NMR, 125 MHz, CDCl3

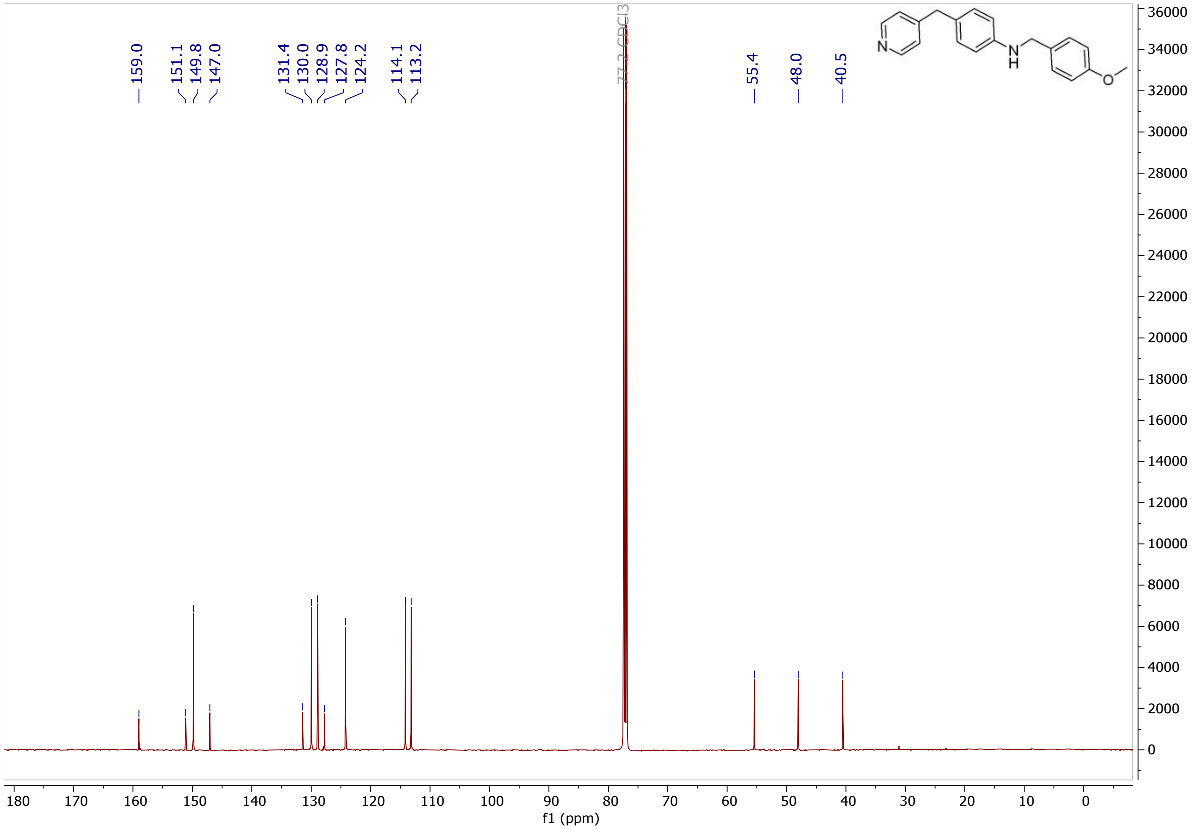

N1-(4-Methoxybenzyl)-N4-methyl-N4-(pyridin-4-yl)benzene-1,4-diamine, (5n), 1H-NMR, 500 MHz, CDCl3

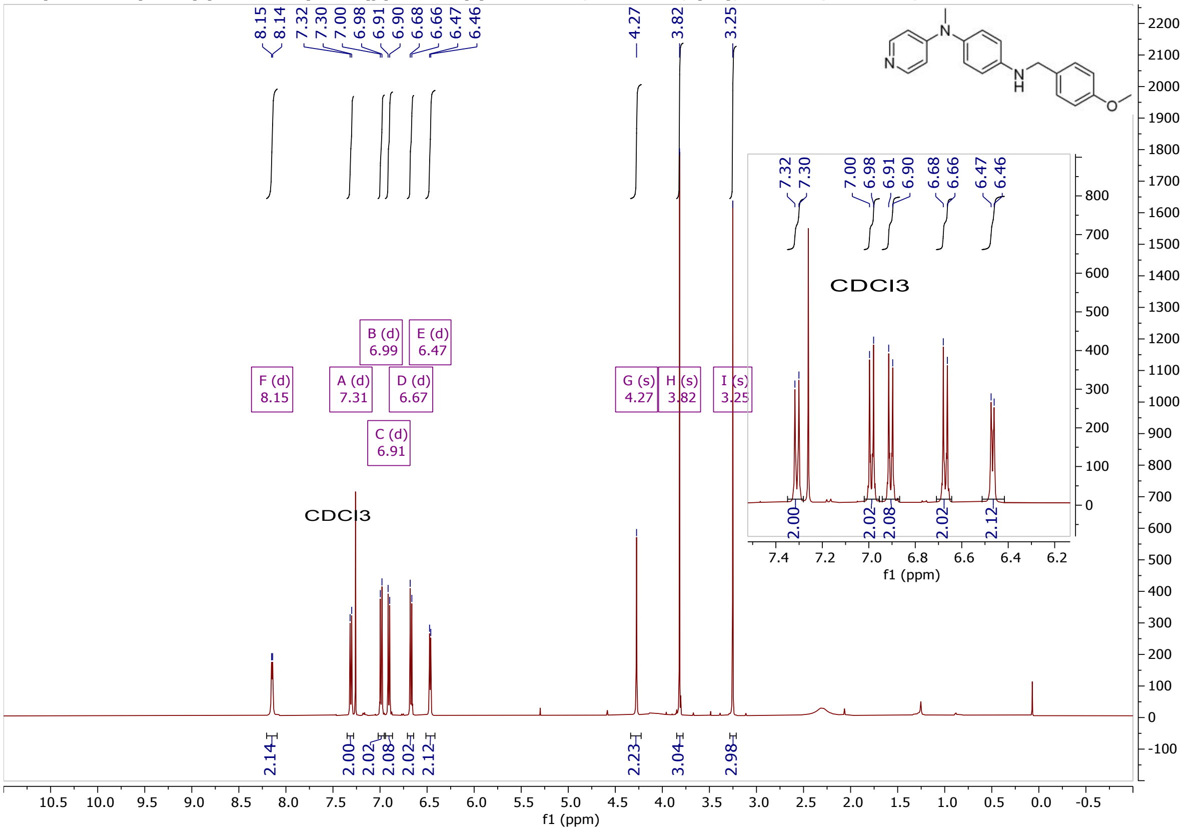

N1-(4-Methoxybenzyl)-N4-methyl-N4-(pyridin-4-yl)benzene-1,4-diamine, (5n), 13C-NMR, 125 MHz, CDCl3

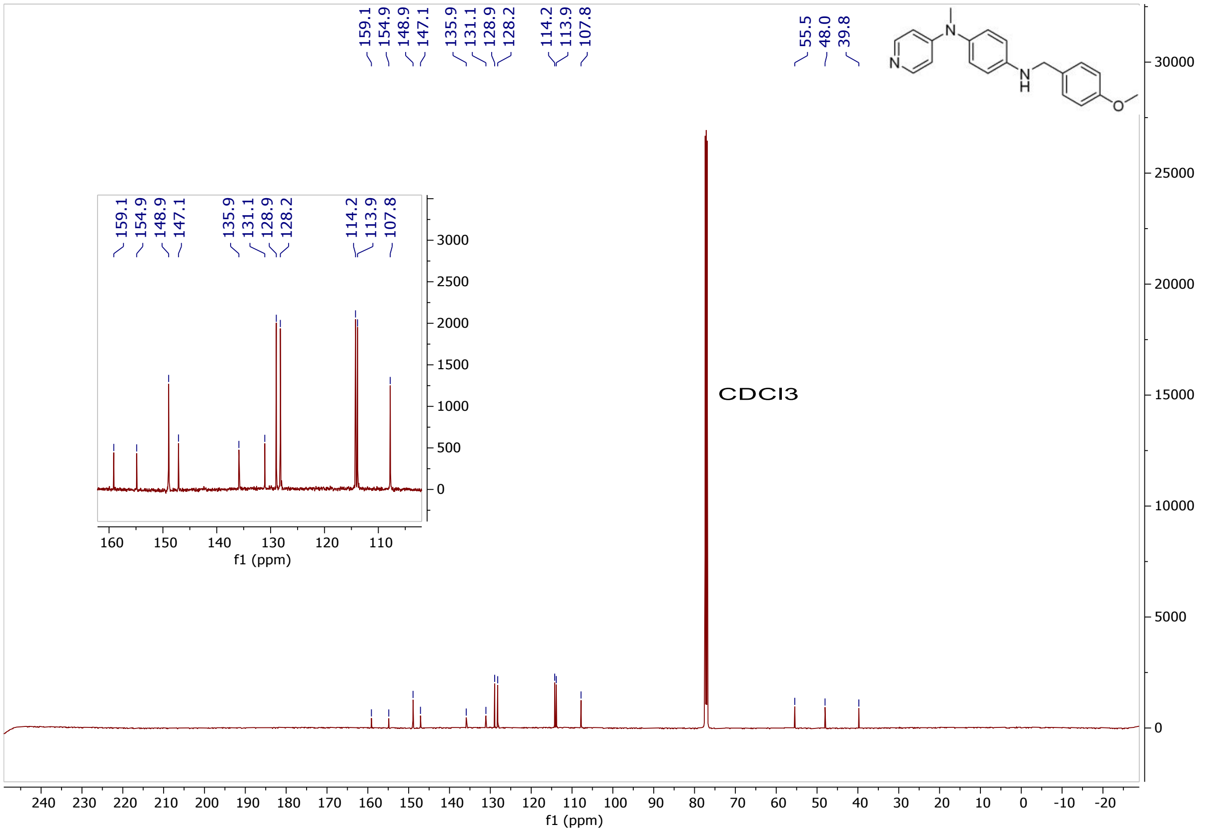

N-(1-(4-Methoxyphenyl)ethyl)-4-(pyridin-4-ylmethyl)aniline, (5o), 1H-NMR, 400 MHz, CDCl3

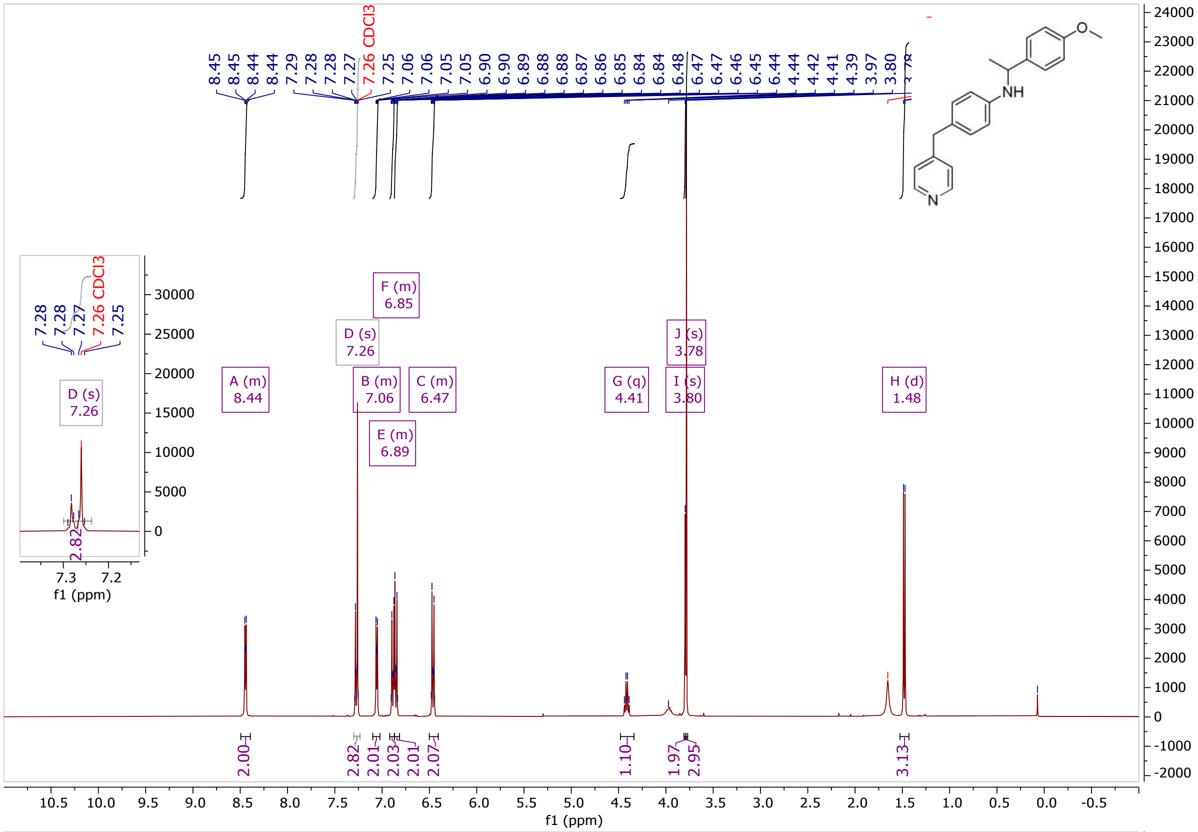

N-(1-(4-Methoxyphenyl)ethyl)-4-(pyridin-4-ylmethyl)aniline, (5o), 13C-NMR, 100 MHz, CDCl3

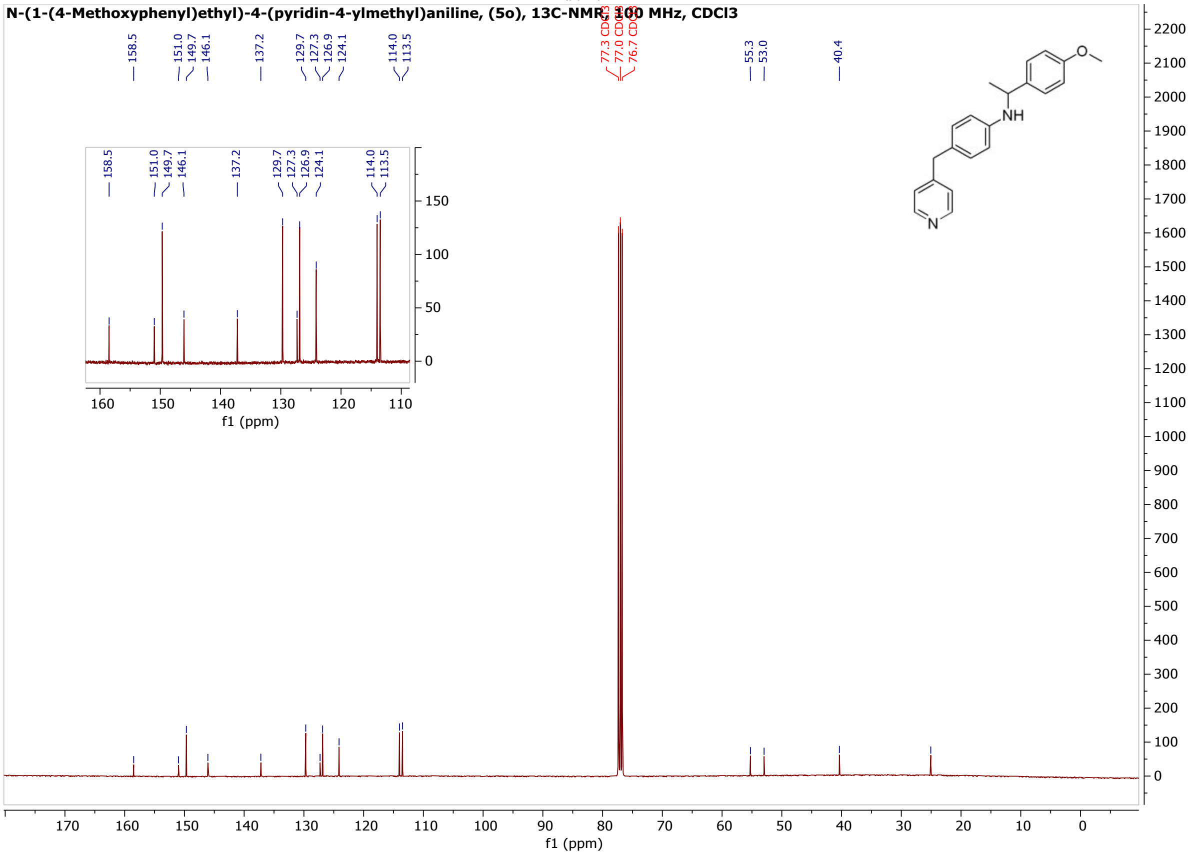

N-(3-(Methylsulfonyl)benzyl)-4-(pyridin-4-ylmethyl)aniline, (5p), 500 MHz, CDCl3

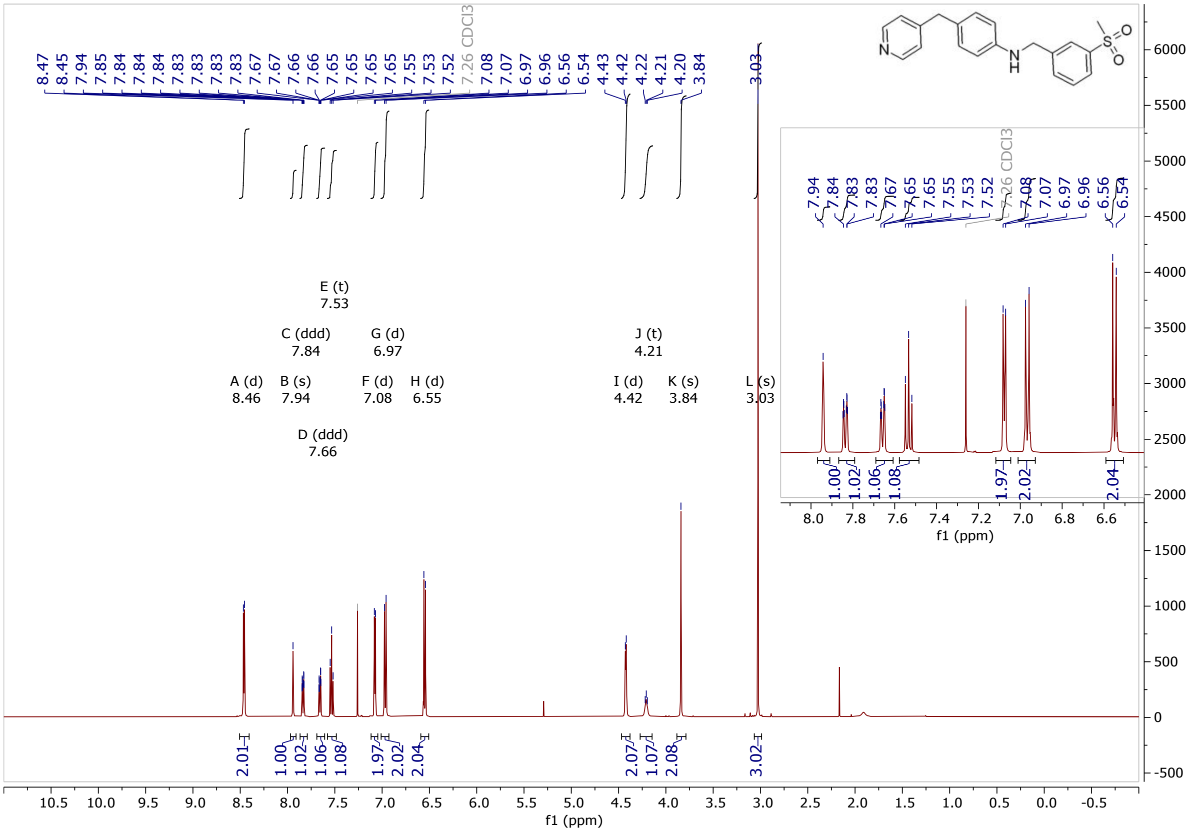

N-(3-(Methylsulfonyl)benzyl)-4-(pyridin-4-ylmethyl)aniline, (5p), 125 MHz, CDCl3

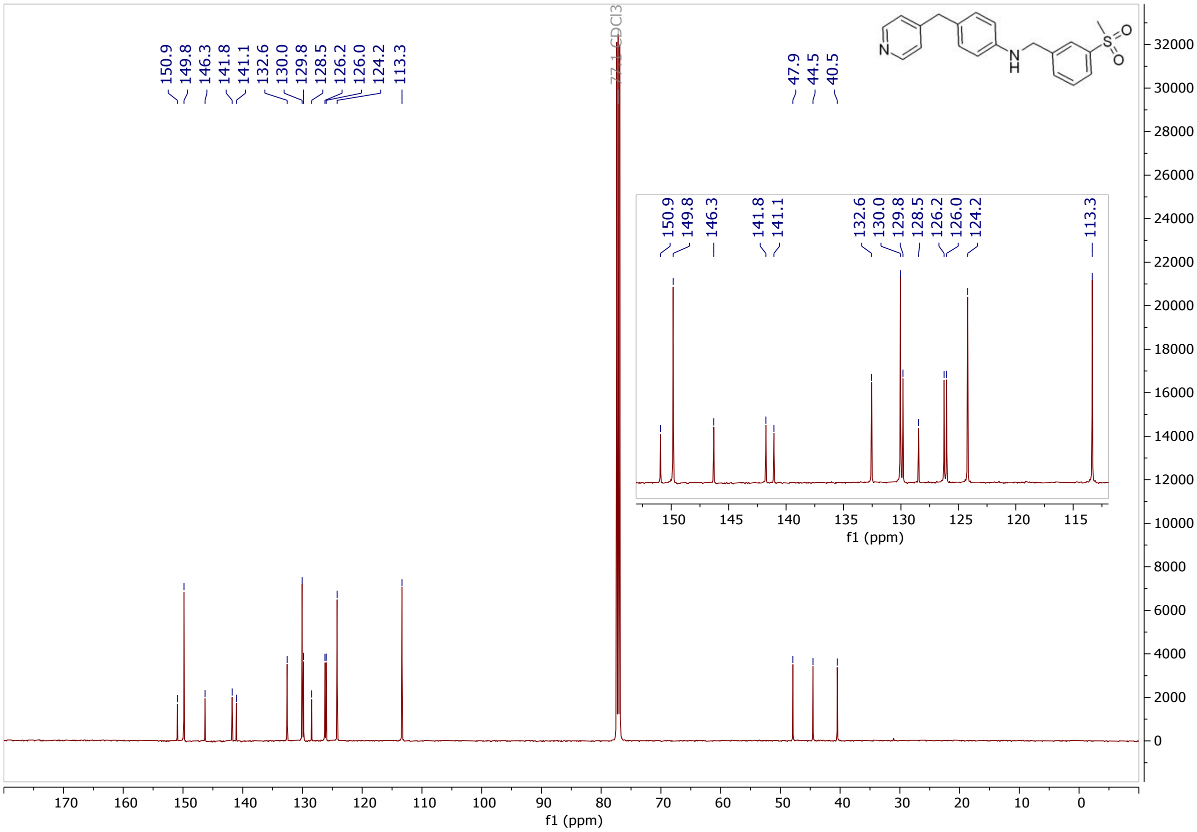

Representative LCMS data

Compound 2a

Openlynx Report - H-Class report M Kavanagh Page 1

ID:MEK137_purity Vial:2:31 JobCode:M Kavanagh2014

File:Abell_Hclass0023290 Date:27-Jul-2016 Time:15:48:32

Description: Method:C:\MassLynx\4_min_Full_Range.olp

Printed: Wed Jul 27 15:52:55 2016

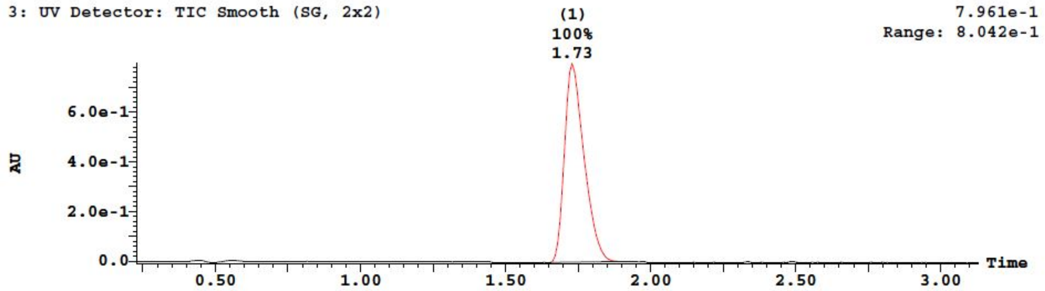

Peak Number	Time	AreaAbs	Area %Total	Height	Mass Found
1	1.73	62795	100.00	799612	

Peak ID	Time	Mass Found
1	1.73	

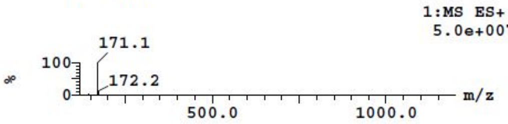

Peak ID	Time	Mass Found
1	1.73	

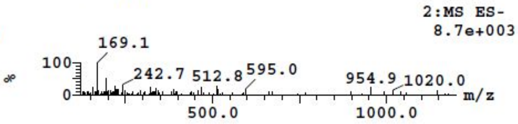

Compound 2b

Openlynx Report - A Coyne Page 1

Sample: 1 Vial:2:27 ID:SG 003 RM

File:Abell_Hclass0008028 Date:20-Oct-2014 Time:09:32:03

Description: Method:C:\MassLynx\4_min_Full_Range.olp

Printed: Mon Oct 20 09:37:53 2014

Sample Report:

Sample 1 Vial 2:27 ID SG 003 RM File Abell_Hclass0008028 Date 20-Oct-2014 Time 09:32:03 Description

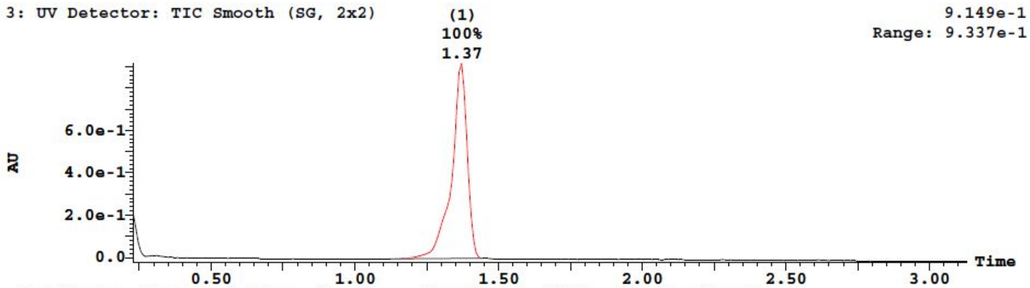

Peak Number	Compound	Time	AreaAbs	Area %Total	Width	Height	Mass Found
1		1.37	6e+004	100.00	0	9e+005	

Peak ID	Compound	Time	Mass Found
1		1.37	

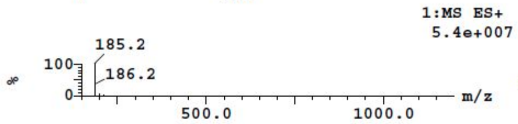

Peak ID	Compound	Time	Mass Found
1		1.37	

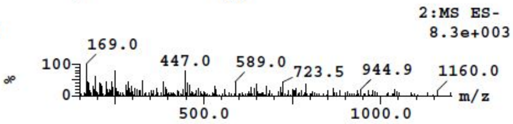

Compound 2d

Openlynx Report - A Coyne Page 1

Sample: 1 Vial:2:7 ID:SG 002 VAC TUBE RERUN
File:Abell_Hclass0008008 Date:17-Oct-2014 Time:15:49:16
Description: Method:C:\MassLynx\4_min_Full_Range.olp

Printed: Fri Oct 17 15:54:07 2014

Sample Report:

Sample 1 Vial 2:7 ID SG 002 VAC TUBE RERUN File Abell_Hclass0008008 Date 17-Oct-2014 Time 15:49:16 Description

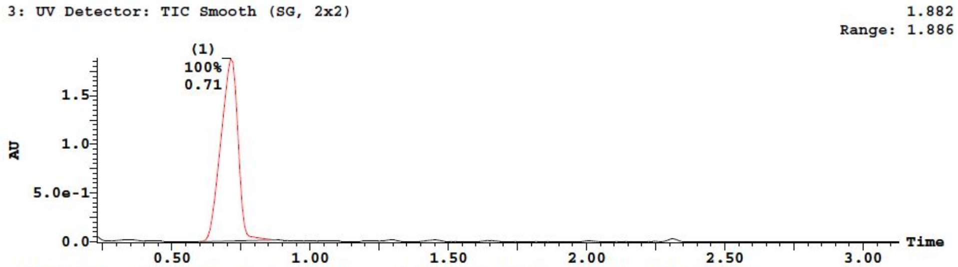

Peak Number	Compound	Time	AreaAbs	Area %Total	Width	Height	Mass Found
1		0.71	1e+005	100.00	0	2e+006	

Peak ID	Compound	Time	Mass Found
1		0.71	

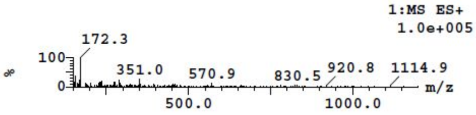

Peak ID	Compound	Time	Mass Found
1		0.71	

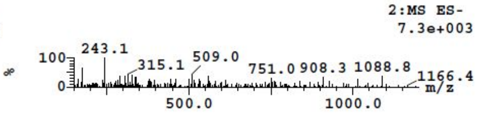

Compound 2g

Openlynx Report - A Coyne Page 1

Sample: 1 Vial:1:28 ID:SG 004 wu
File:Abell_Hclass0008077 Date:22-Oct-2014 Time:09:51:06
Description: Method:C:\MassLynx\4_min_Full_Range.olp

Printed: Wed Oct 22 09:58:22 2014

Sample Report:

Sample 1 Vial 1:28 ID SG 004 wu File Abell_Hclass0008077 Date 22-Oct-2014 Time 09:51:06 Description

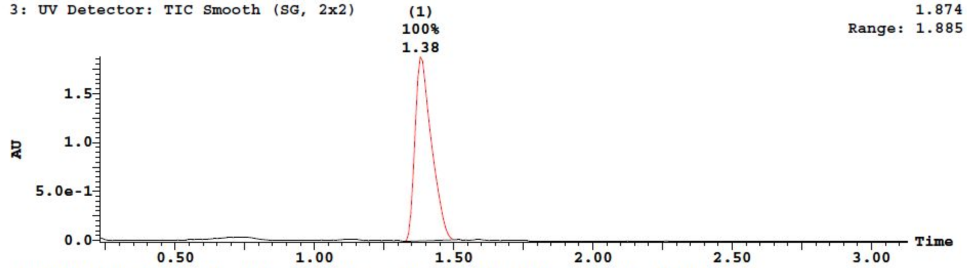

Peak Number	Compound	Time	AreaAbs	Area %Total	Width	Height	Mass Found
1		1.38	1e+005	100.00	0	2e+006	

Peak ID	Compound	Time	Mass Found
1		1.38	

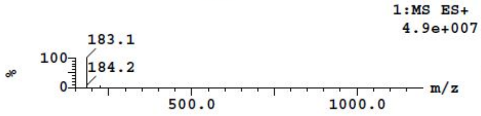

Peak ID	Compound	Time	Mass Found
1		1.38	

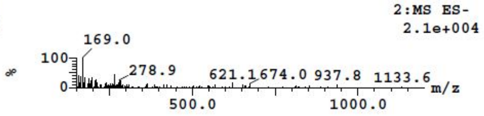

Compound 2h

Openlynx Report - A Coyne Page 1

Sample: 1 Vial:1:45 ID:SG 005 o/n

File:Abell_Hclass0008094 Date:23-Oct-2014 Time:09:25:09

Description: Method:C:\MassLynx\4_min_Full_Range.olp

Printed: Thu Oct 23 09:29:58 2014

Sample Report:

Sample 1 Vial 1:45 ID SG 005 o/n File Abell_Hclass0008094 Date 23-Oct-2014 Time 09:25:09 Description

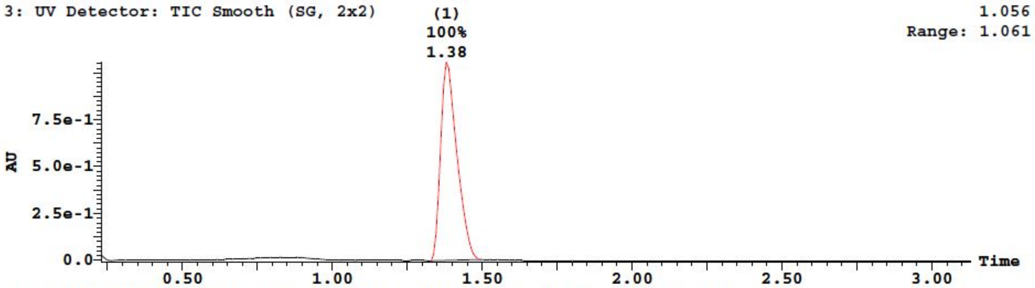

Peak Number	Compound	Time	AreaAbs	Area %Total	Width	Height	Mass Found
1		1.38	7e+004	100.00	0	1e+006	

Peak ID	Compound	Time	Mass Found
1		1.38	

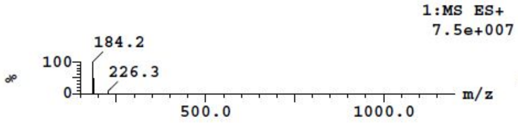

Peak ID	Compound	Time	Mass Found
1		1.38	

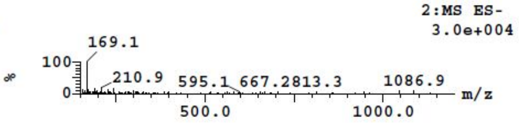

Compound 4b

Openlynx Report - H-Class report M Kavanagh Page 1

ID:mek130 Vial:2:16 JobCode:M Kavanagh1161

File:Abell_Hclass0012331 Date:28-May-2015 Time:16:58:11

Description: Method:C:\MassLynx\4_min_Full_Range.olp

Printed: Thu May 28 17:02:31 2015

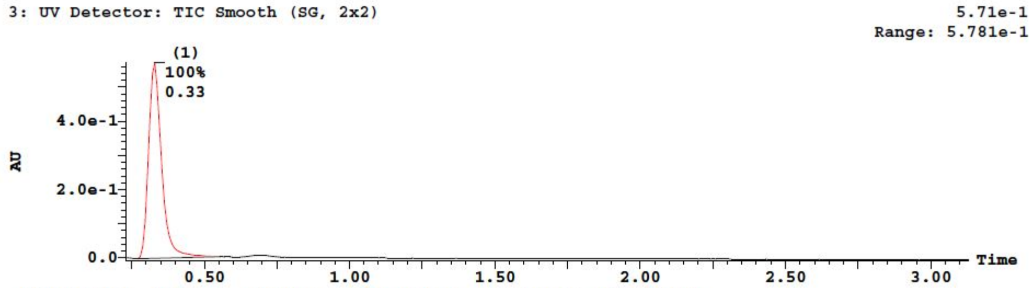

Peak Number	Time	AreaAbs	Area %Total	Height	Mass Found
1	0.33	29957	100.00	572661	

Peak ID	Time	Mass Found
1	0.33	

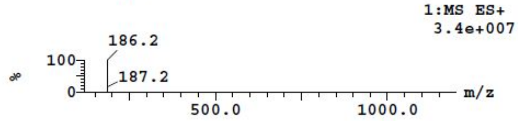

Peak ID	Time	Mass Found
1	0.33	

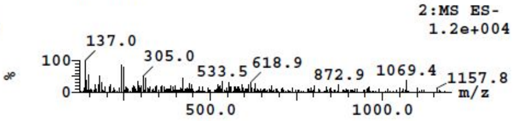

Compound 4c

(methyl ester, LCMS (+ESI) *m/z* 229.0 $[M+H]^+$, retention time 1.38 min, ethyl ester, LCMS (+ESI) *m/z* 243.0 $[M+H]^+$, retention time 1.53 min)

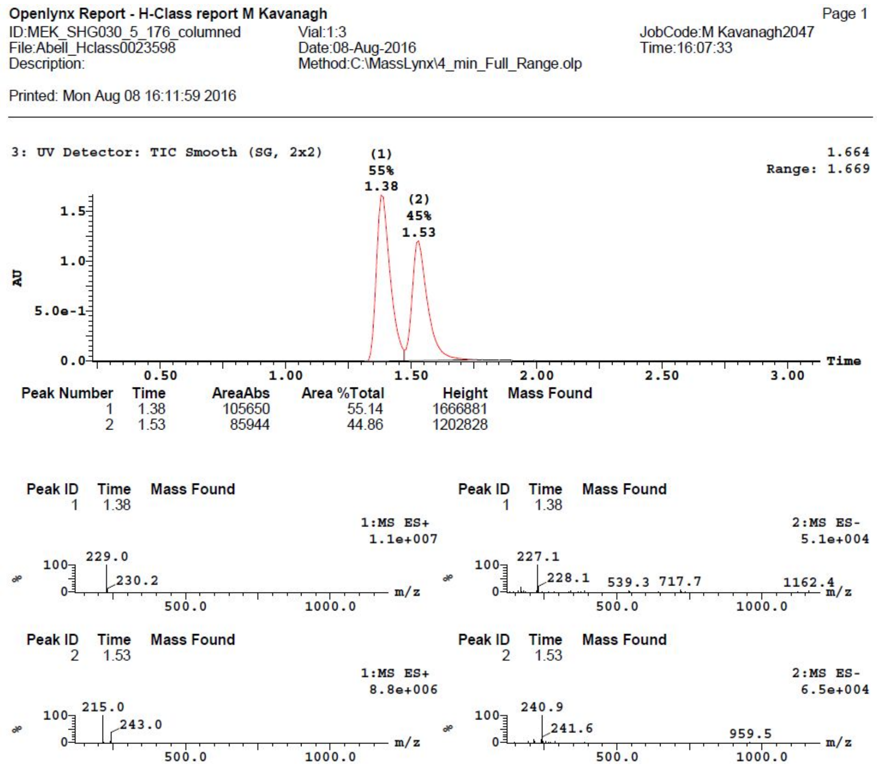

Compound 4d

(LCMS (+ESI) m/z 229.0 [M+H]+, retention time 1.26 min (95%))

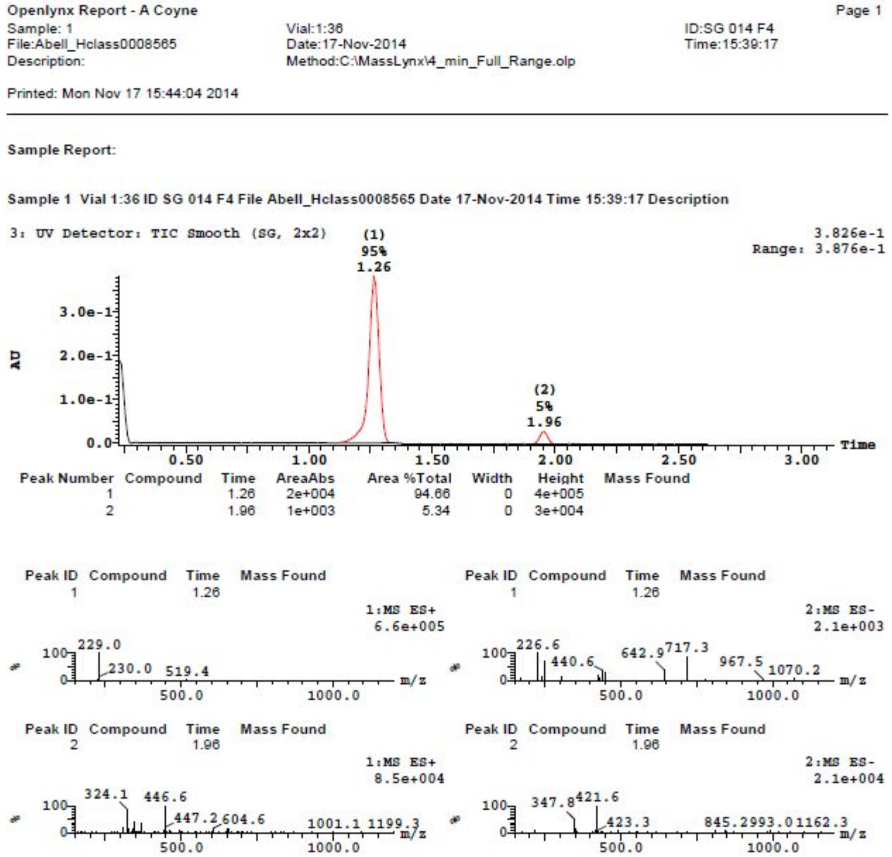

Compound 4e

LCMS (+ESI) m/z 215.0, 215.0 [M+H]+, retention time 0.31, 1.19 min, (17%, 83%)

Openlynx Report - H-Class report M Kavanagh Page 1

ID:mek_shg030_hydrolysis_3 h | Vial:1:19 | JobCode:M Kavanagh2048
File:Abell_Hclass0023614 | Date:09-Aug-2016 | Time:13:50:05
Description: | Method:C:\MassLynx\4_min_Full_Range.olp

Printed: Tue Aug 09 13:54:30 2016

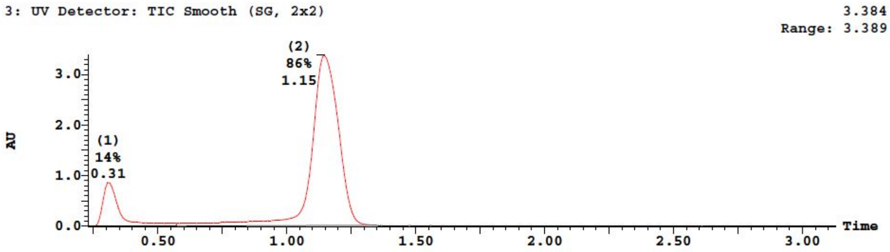

Peak Number	Time	AreaAbs	Area %Total	Height	Mass Found
1	0.31	64903	13.62	863121	
2	1.15	411799	86.38	3372731	

Peak ID	Time	Mass Found
1	0.31	

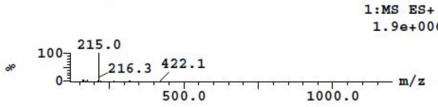

Peak ID	Time	Mass Found
1	0.31	

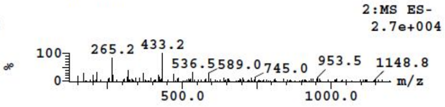

Peak ID	Time	Mass Found
2	1.15	

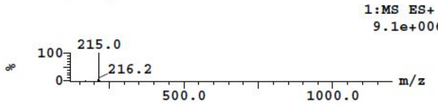

Peak ID	Time	Mass Found
2	1.15	

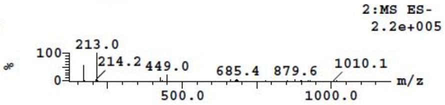

Compound 4f

Openlynx Report - A Coyne Page 1

Sample: 1 | Vial:1:4 | ID:SG 023 FD
File:Abell_Hclass0009013 | Date:05-Dec-2014 | Time:13:32:37
Description: | Method:C:\MassLynx\4_min_Full_Range.olp

Printed: Fri Dec 05 13:38:28 2014

Sample Report:

Sample 1 Vial 1:4 ID SG 023 FD File Abell_Hclass0009013 Date 05-Dec-2014 Time 13:32:37 Description

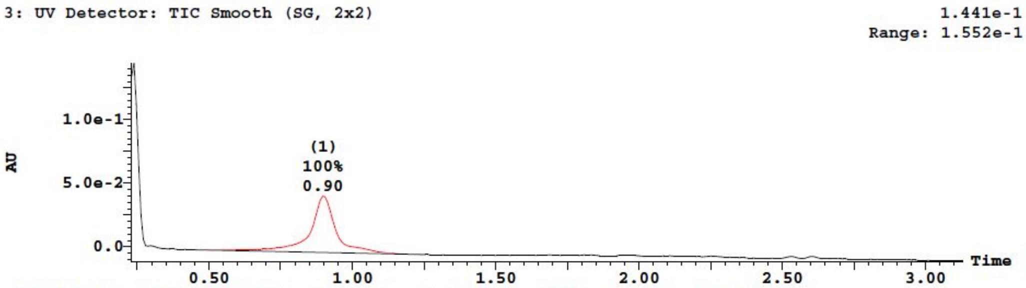

Peak Number	Compound	Time	AreaAbs	Area %Total	Width	Height	Mass Found
1		0.90	5e+003	100.00	1	4e+004	

Peak ID	Compound	Time	Mass Found
1		0.90	

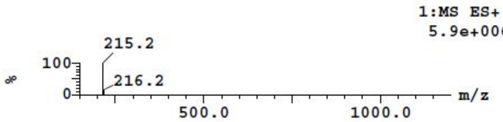

Peak ID	Compound	Time	Mass Found
1		0.90	

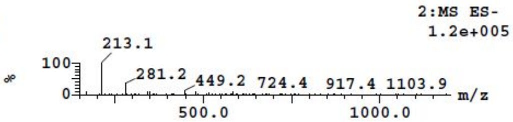

Compound 4i

Openlynx Report - A Coyne Page 1

Sample: 1 Vial:2:19 ID:SG 011 Fr2
File:Abell_Hclass0008404 Date:07-Nov-2014 Time:17:04:45
Description: Method:C:\MassLynx\4_min_Full_Range.olp

Printed: Fri Nov 07 17:11:27 2014

Sample Report:

Sample 1 Vial 2:19 ID SG 011 Fr2 File Abell_Hclass0008404 Date 07-Nov-2014 Time 17:04:45 Description

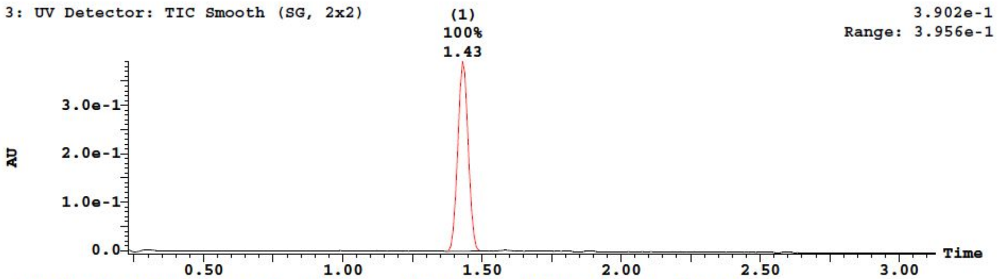

Peak Number	Compound	Time	AreaAbs	Area %Total	Width	Height	Mass Found
1		1.43	2e+004	100.00	0	4e+005	

Peak ID	Compound	Time	Mass Found
1		1.43	

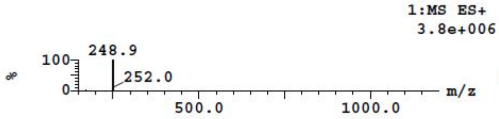

Peak ID	Compound	Time	Mass Found
1		1.43	

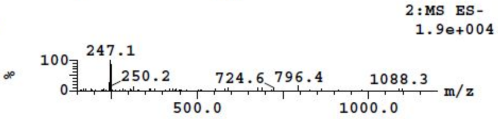

Compound 5a

Openlynx Report - H-Class report M Kavanagh Page 1

ID:MEK_AGC869_5_156_final Vial:2:22 JobCode:M Kavanagh1985
File:Abell_Hclass0022417 Date:25-Jun-2016 Time:18:29:49
Description: Method:C:\MassLynx\4_min_Full_Range.olp

Printed: Sat Jun 25 18:35:09 2016

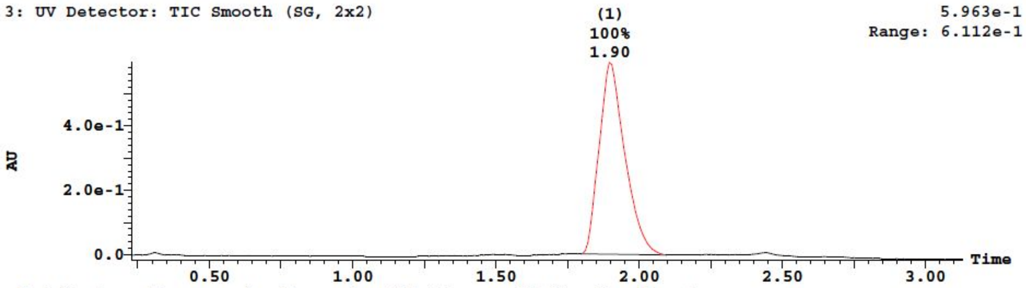

Peak Number	Time	AreaAbs	Area %Total	Height	Mass Found
1	1.90	61959	100.00	594344	

Peak ID	Time	Mass Found
1	1.90	

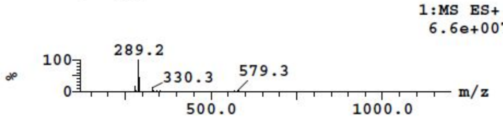

Peak ID	Time	Mass Found
1	1.90	

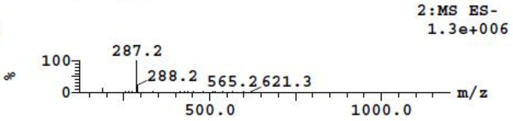

Compound 5b

Openlynx Report - H-Class report M Kavanagh Page 1

ID:mek131	Vial:1:34	JobCode:M Kavanagh1173
File:Abell_Hclass0012397	Date:01-Jun-2015	Time:17:15:40
Description:	Method:C:\MassLynx\4_min_Full_Range.olp	

Printed: Mon Jun 01 17:21:00 2015

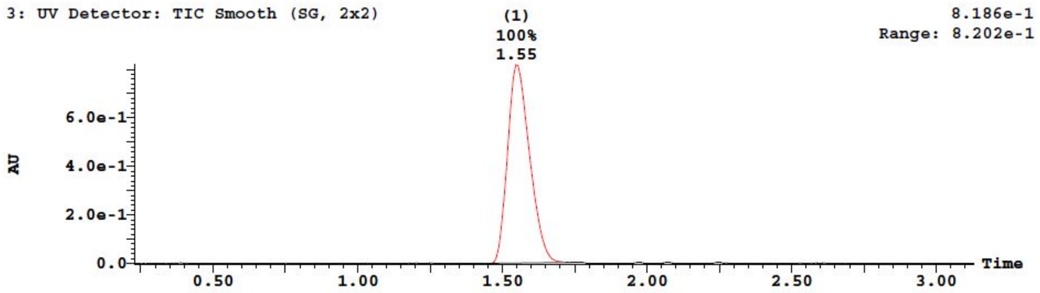

Peak Number	Time	AreaAbs	Area %Total	Height	Mass Found
1	1.55	72211	100.00	816639	

Peak ID	Time	Mass Found
1	1.55	

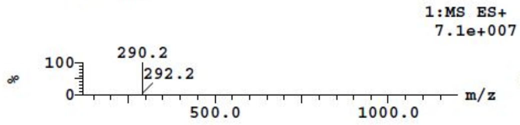

Peak ID	Time	Mass Found
1	1.55	

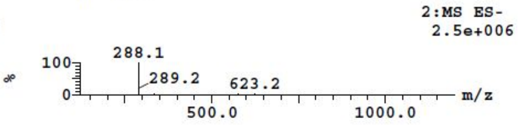

Compound 5c

Openlynx Report - H-Class report A Fanourakis Page 1

ID:AF010 Pure	Vial:1:44	JobCode:A Fanourakis63
File:Abell_Hclass0023927	Date:25-Aug-2016	Time:11:33:03
Description:	Method:C:\MassLynx\4_min_Full_Range.olp	

Printed: Thu Aug 25 11:37:30 2016

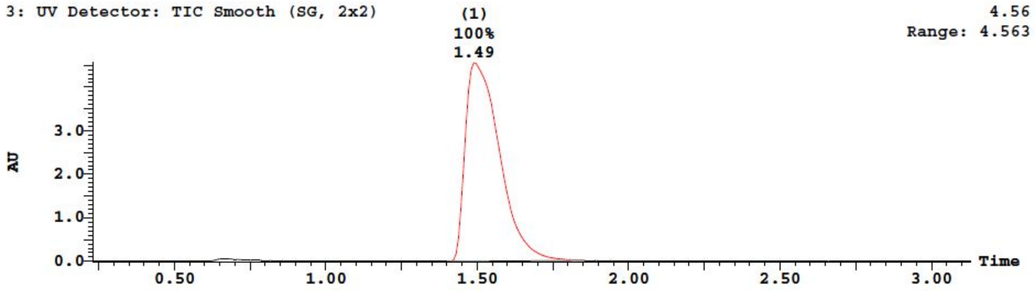

Peak Number	Time	AreaAbs	Area %Total	Height	Mass Found
1	1.49	601146	100.00	4557368	

Peak ID	Time	Mass Found
1	1.49	

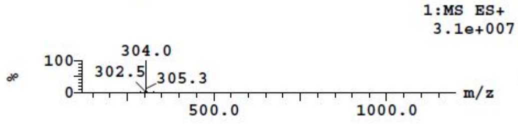

Peak ID	Time	Mass Found
1	1.49	

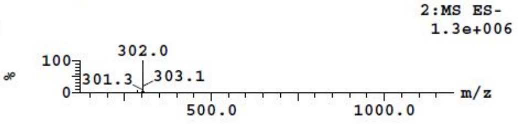

Compound 5d

Openlynx Report - H-Class report M Kavanagh Page 1

ID:mek200_5_74_col1 Vial:2:3 JobCode:M Kavanagh1695
File:Abell_Hclass0017694 Date:25-Jan-2016 Time:13:25:34
Description: Method:C:\MassLynx\4_min_Full_Range.olp

Printed: Mon Jan 25 13:30:42 2016

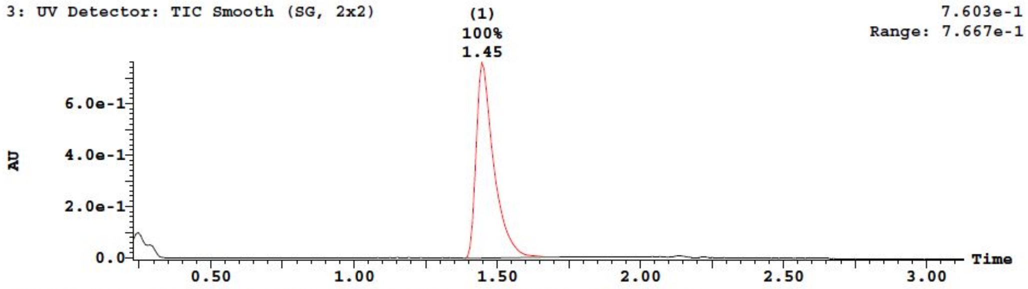

Peak Number	Time	AreaAbs	Area %Total	Height	Mass Found
1	1.45	54464	100.00	761195	

Peak ID	Time	Mass Found
1	1.45	

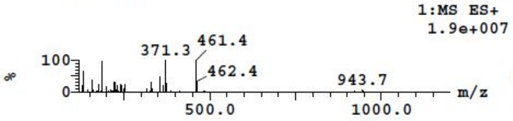

Peak ID	Time	Mass Found
1	1.45	

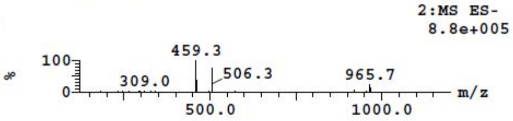

Compound 5e

Openlynx Report - H-Class report M Kavanagh Page 1

ID:mek212 Vial:1:16 JobCode:M Kavanagh2078
File:Abell_Hclass0025819 Date:14-Dec-2016 Time:15:15:16
Description: Method:C:\MassLynx\4_min_Full_Range.olp

Printed: Wed Dec 14 15:19:37 2016

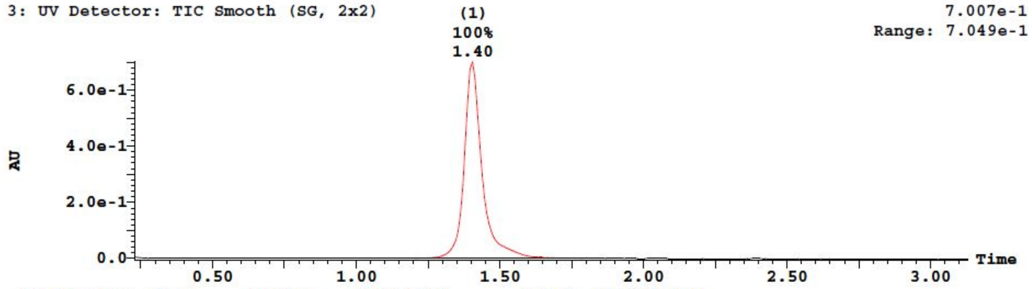

Peak Number	Time	AreaAbs	Area %Total	Height	Mass Found
1	1.40	50974	100.00	700672	

Peak ID	Time	Mass Found
1	1.40	

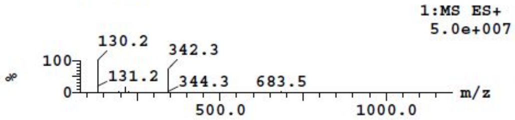

Peak ID	Time	Mass Found
1	1.40	

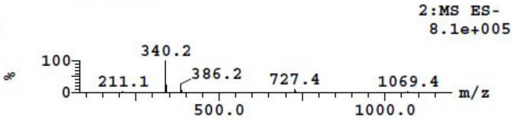

Compound 5f

Openlynx Report - H-Class report M Kavanagh Page 1

ID:mek205_5_88_columned again Vial:2:10 JobCode:M Kavanagh1755
File:Abell_Hclass0018085 Date:05-Feb-2016 Time:13:32:34
Description: Method:C:\MassLynx\4_min_Full_Range.olp

Printed: Fri Feb 05 13:37:55 2016

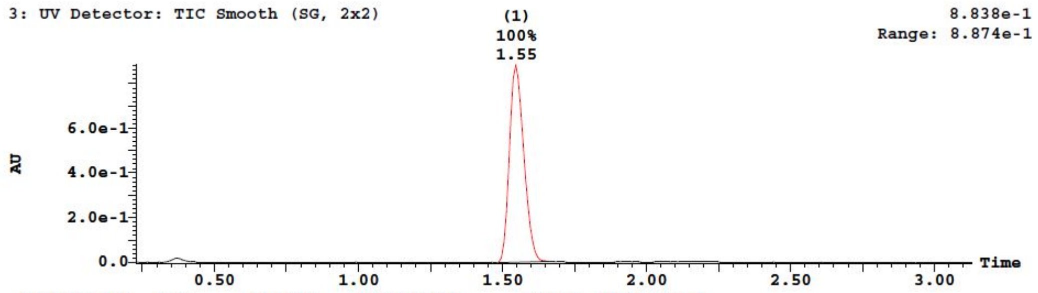

Peak Number	Time	AreaAbs	Area %Total	Height	Mass Found
1	1.55	51344	100.00	883586	

Peak ID	Time	Mass Found
1	1.55	

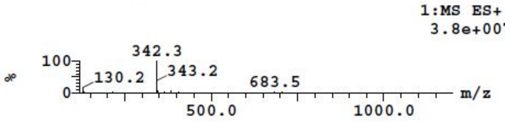

Peak ID	Time	Mass Found
1	1.55	

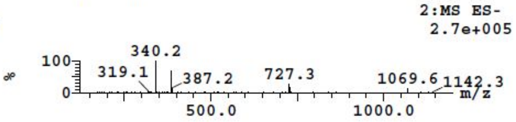

Compound 5i

Openlynx Report - H-Class report M Kavanagh Page 1

ID:mek210_5_116_col1 Vial:1:30 JobCode:M Kavanagh1870
File:Abell_Hclass0019305 Date:07-Mar-2016 Time:10:58:53
Description: Method:C:\MassLynx\4_min_Full_Range.olp

Printed: Mon Mar 07 11:03:13 2016

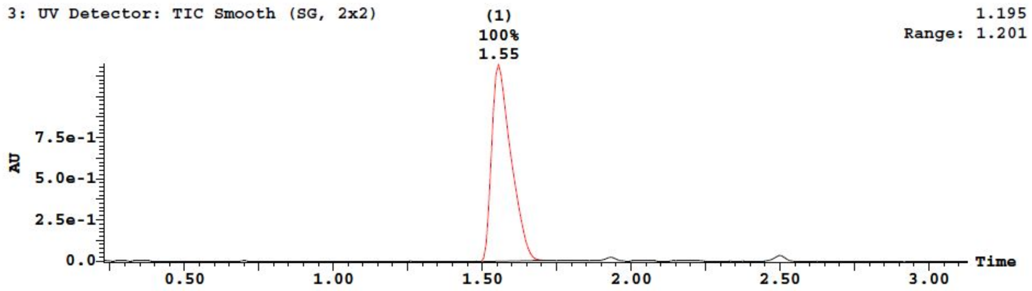

Peak Number	Time	AreaAbs	Area %Total	Height	Mass Found
1	1.55	89652	100.00	1196784	

Peak ID	Time	Mass Found
1	1.55	

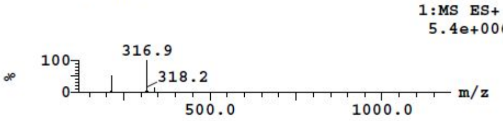

Peak ID	Time	Mass Found
1	1.55	

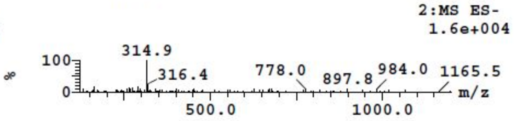

Compound 5j

LCMS (+ESI) m/z 325.2 [M+H]+, retention time 1.40 min, (95%)

Openlynx Report - H-Class report M Kavanagh Page 1

ID:mek_AGC871 Vial:1:17 JobCode:M Kavanagh2079
File:Abell_Hclass0025820 Date:14-Dec-2016 Time:15:19:37
Description: Method:C:\MassLynx\4_min_Full_Range.olp

Printed: Wed Dec 14 15:24:00 2016

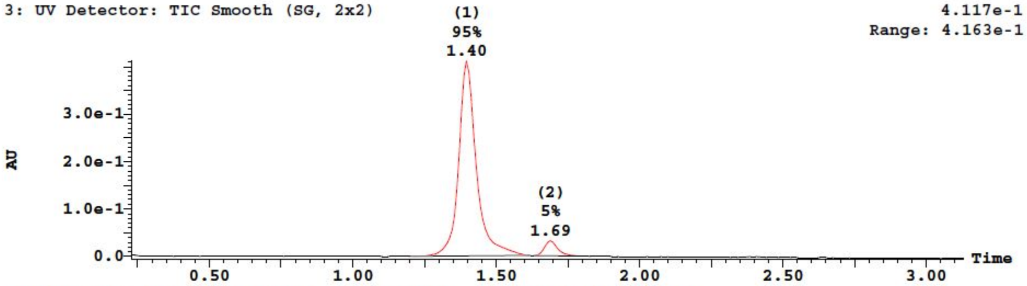

Peak Number	Time	AreaAbs	Area %Total	Height	Mass Found
1	1.40	29382	94.58	410463	
2	1.69	1683	5.42	31596	

Peak ID 1 Time 1.40 Mass Found

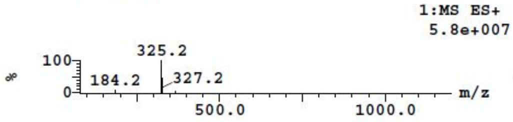

Peak ID 1 Time 1.40 Mass Found

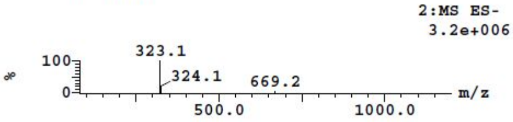

Peak ID 2 Time 1.69 Mass Found

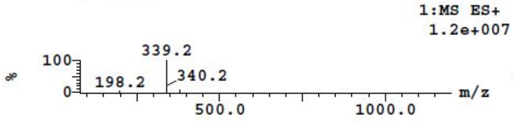

Peak ID 2 Time 1.69 Mass Found

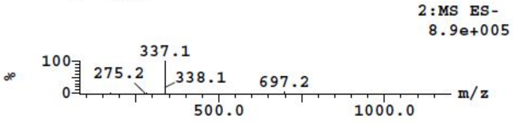

Compound 5k

Openlynx Report - H-Class report M Kavanagh Page 1

ID:mek197_5_64_columned	Vial:1:35	JobCode:M Kavanagh1679
File:Abell_Hclass0017582	Date:21-Jan-2016	Time:14:19:30
Description:	Method:C:\MassLynx\4_min_Full_Range.olp	

Printed: Thu Jan 21 14:23:54 2016

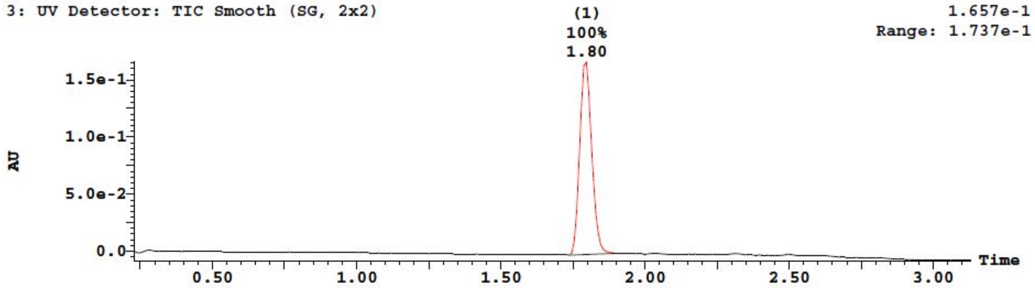

Peak Number	Time	AreaAbs	Area %Total	Height	Mass Found
1	1.80	8206	100.00	168360	

Peak ID	Time	Mass Found
1	1.80	

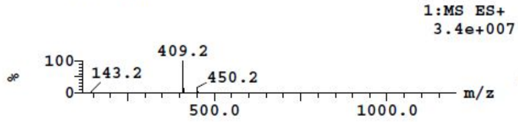

Peak ID	Time	Mass Found
1	1.80	

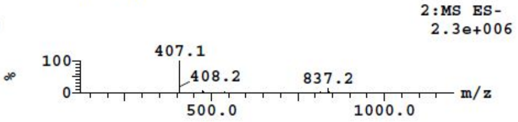

Compound 5l

Openlynx Report - H-Class report M Kavanagh Page 1

ID:AF001 Pure	Vial:2:21	JobCode:M Kavanagh2001
File:Abell_Hclass0022896	Date:13-Jul-2016	Time:13:34:03
Description:	Method:C:\MassLynx\4_min_Full_Range.olp	

Printed: Wed Jul 13 13:39:25 2016

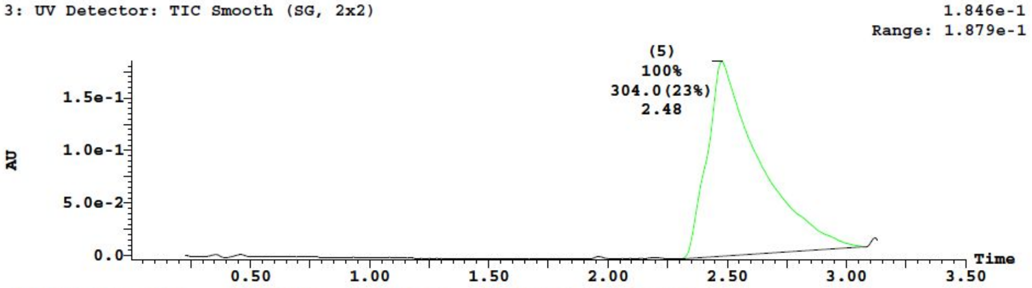

Peak Number	Time	AreaAbs	Area %Total	Height	Mass Found
5	2.48	47337	100.00	184948	304.00

Peak ID	Time	Mass Found
5	2.45	305.00

Peak ID	Time	Mass Found
5	2.45	

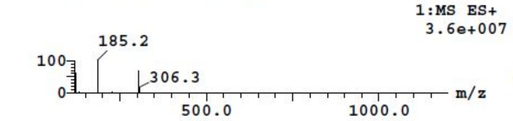

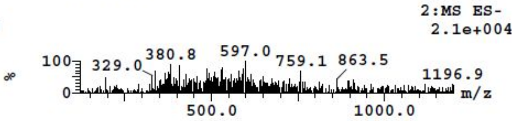

Compound 5m

Openlynx Report - H-Class report M Kavanagh Page 1

ID:MEK216_5_122_columned	Vial:2:16	JobCode:M Kavanagh1894
File:Abell_Hclass0019723	Date:21-Mar-2016	Time:15:49:46
Description:	Method:C:\MassLynx\4_min_Full_Range.olp	

Printed: Mon Mar 21 15:54:27 2016

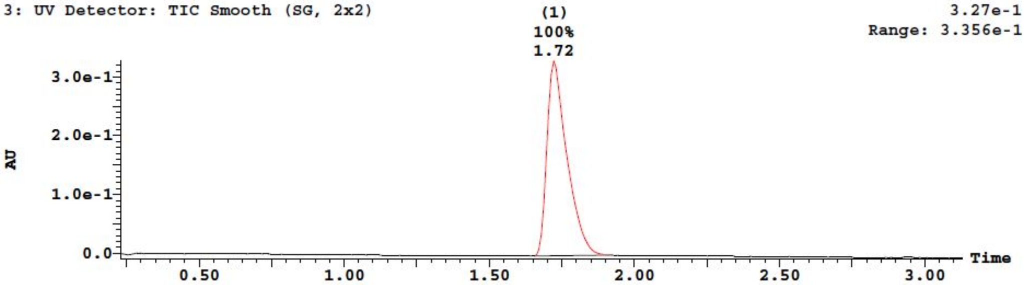

Peak Number	Time	AreaAbs	Area %Total	Height	Mass Found
1	1.72	26482	100.00	331192	

Peak ID	Time	Mass Found
1	1.72	

Peak ID	Time	Mass Found
1	1.72	

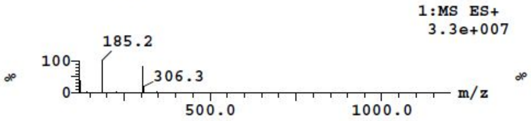

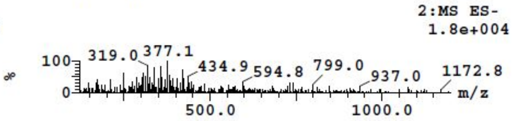

Compound 5n

Openlynx Report - H-Class report A Fanourakis Page 1

ID:AF008 F12 Vial:2:47 JobCode:A Fanourakis50
File:Abell_Hclass0023786 Date:17-Aug-2016 Time:14:20:32
Description: Method:C:\MassLynx\4_min_Full_Range.olp

Printed: Wed Aug 17 14:25:53 2016

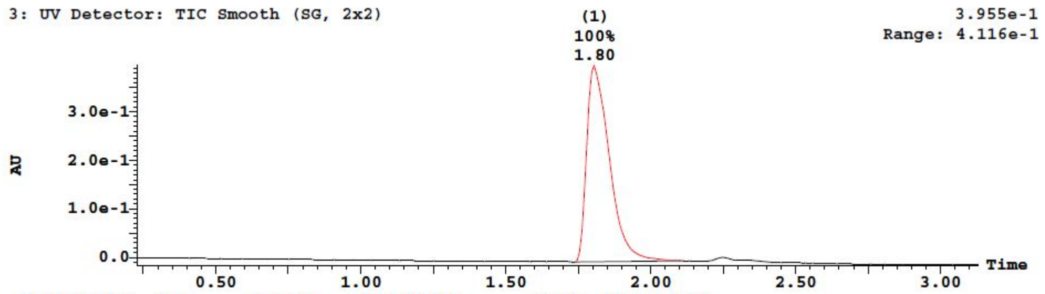

Peak Number	Time	AreaAbs	Area %Total	Height	Mass Found
1	1.80	36825	100.00	404809	

Peak ID	Time	Mass Found
1	1.80	

Peak ID	Time	Mass Found
1	1.80	

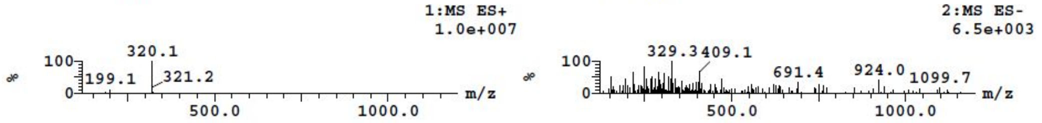

Compound 5o

Openlynx Report - H-Class report M Kavanagh Page 1

ID:AF003 Vial:1:18 JobCode:M Kavanagh2080
File:Abell_Hclass0025821 Date:14-Dec-2016 Time:15:23:58
Description: Method:C:\MassLynx\4_min_Full_Range.olp

Printed: Wed Dec 14 15:28:22 2016

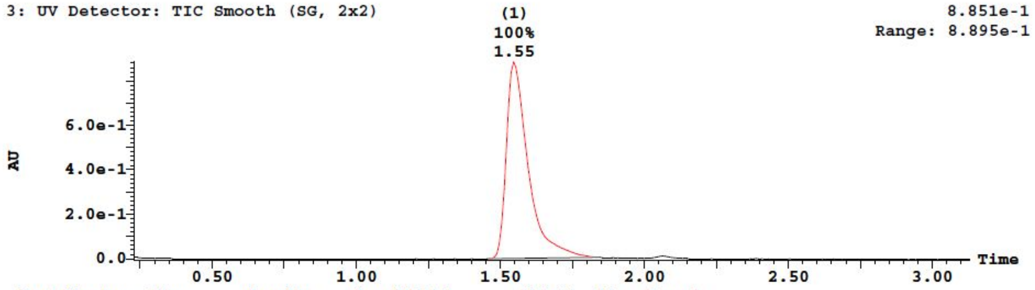

Peak Number	Time	AreaAbs	Area %Total	Height	Mass Found
1	1.55	77954	100.00	883540	

Peak ID	Time	Mass Found
1	1.55	

Peak ID	Time	Mass Found
1	1.55	

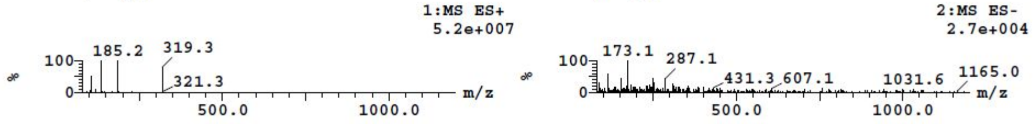

Compound 5p

Openlynx Report - H-Class report M Kavanagh Page 1

ID:MEK217_5_124_columned Vial:2:17 JobCode:M Kavanagh1895
File:Abell_Hclass0019724 Date:21-Mar-2016 Time:15:54:07
Description: Method:C:\MassLynx\4_min_Full_Range.olp

Printed: Mon Mar 21 15:58:25 2016

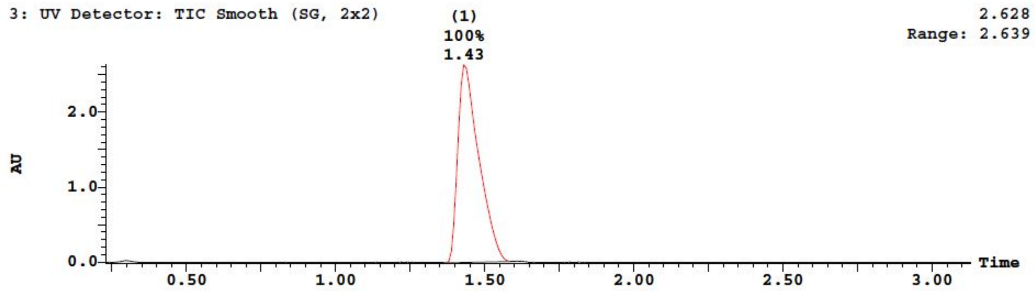

Peak Number	Time	AreaAbs	Area %Total	Height	Mass Found
1	1.43	215810	100.00	2633743	

Peak ID	Time	Mass Found
1	1.43	

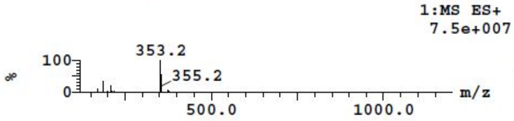

Peak ID	Time	Mass Found
1	1.43	

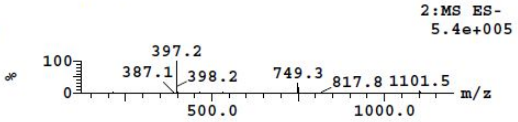

Supplement: Supplementary file 1 [file jm5c00478_si_001.pdf]
